# Sustainable Polymers as System-Dependent Technologies: A Critical Engineering Assessment of LCA Evidence, Circular Compatibility, and Scalability Constraints

**DOI:** 10.3390/polym18202467

**Published:** 2026-10-09

**Authors:** Sergiu Lazăr, Dan Dobrotă, Ionela Magdalena Rotaru, Gabriela Dobrotă

**Affiliations:** 1 Department of Industrial Engineering and Management, Faculty of Engineering, Lucian Blaga University of Sibiu, 550024 Sibiu, Romania; sergiu.lazar@ulbsibiu.ro; 2 S.C. Fusion România S.R.L., Ștefan Cel Mare Street, No. 152-154, 550321 Sibiu, Romania; ionela.rotaru@fusion.ro; 3 Faculty of Economics, “Constantin Brâncuși” University of Targu Jiu, 210185 Târgu Jiu, Romania; gabriela.dobrota@e-ucb.ro

**Keywords:** sustainable polymers, bioplastics, circular economy, life cycle assessment, polymers engineering, renewable polymers, green plastics

## Abstract

The transition toward sustainable polymer systems is frequently framed as a material substitution challenge. However, the environmental performance of bio-based, biodegradable, and recyclable polymers is not an intrinsic property of feedstock origin but a system-dependent outcome shaped by energy inputs, infrastructure compatibility, economic feasibility, and governance alignment. This review provides a critical engineering assessment of sustainable polymer technologies based on systematic evidence screening (*n* = 193), bibliometric analysis, life cycle assessment (LCA) synthesis, techno-economic indicators, end-of-life scenario interpretation, and a multi-criteria evidence synthesis based on source-linked quantitative indicators and categorical evidence. To avoid treating these components as parallel but disconnected analyses, the synthesis explicitly links bibliometric clusters with LCA, TEA, and infrastructure evidence categories through an evidence-gap mapping matrix and confidence grading scheme. The findings indicate that bio-based polymers, such as PLA, PHA, and PBS, may achieve reduced greenhouse gas emissions under optimized production conditions; however, these advantages are highly sensitive to electricity carbon intensity, land-use assumptions, and end-of-life infrastructure. Quantitative LCA evidence indicates that polylactic acid (PLA) may achieve greenhouse gas emission reductions in the range of approximately 25–55% compared to conventional PET under low-carbon energy scenarios (1.3–3.0 vs. 2.0–3.5 kg CO_2_e/kg), while polyhydroxyalkanoates (PHA) show reductions of 10–40%, depending on fermentation efficiency and feedstock selection. However, these benefits can diminish or reverse under fossil-intensive energy mixes or when land-use change impacts are included, reinforcing the system-dependent nature of sustainability performance. Recyclability within established material streams frequently provides more stable long-term performance than biodegradable alternatives deployed without controlled composting systems. Evidence-strength analysis reveals moderate confidence in carbon footprint comparisons, weak-to-moderate confidence in techno-economic scalability claims, and conditional confidence in infrastructure-dependent outcomes. The results demonstrate that sustainable polymers are system-dependent technologies rather than inherently sustainable materials. Future engineering research must prioritize integrated LCA–TEA modeling, infrastructure-aligned material design, scenario-based end-of-life assessment, and harmonized sustainability reporting standards to enable scalable and verifiable circular polymer systems.

## 1. Introduction

### 1.1. The Plastic Dilemma

**Figure 1:**
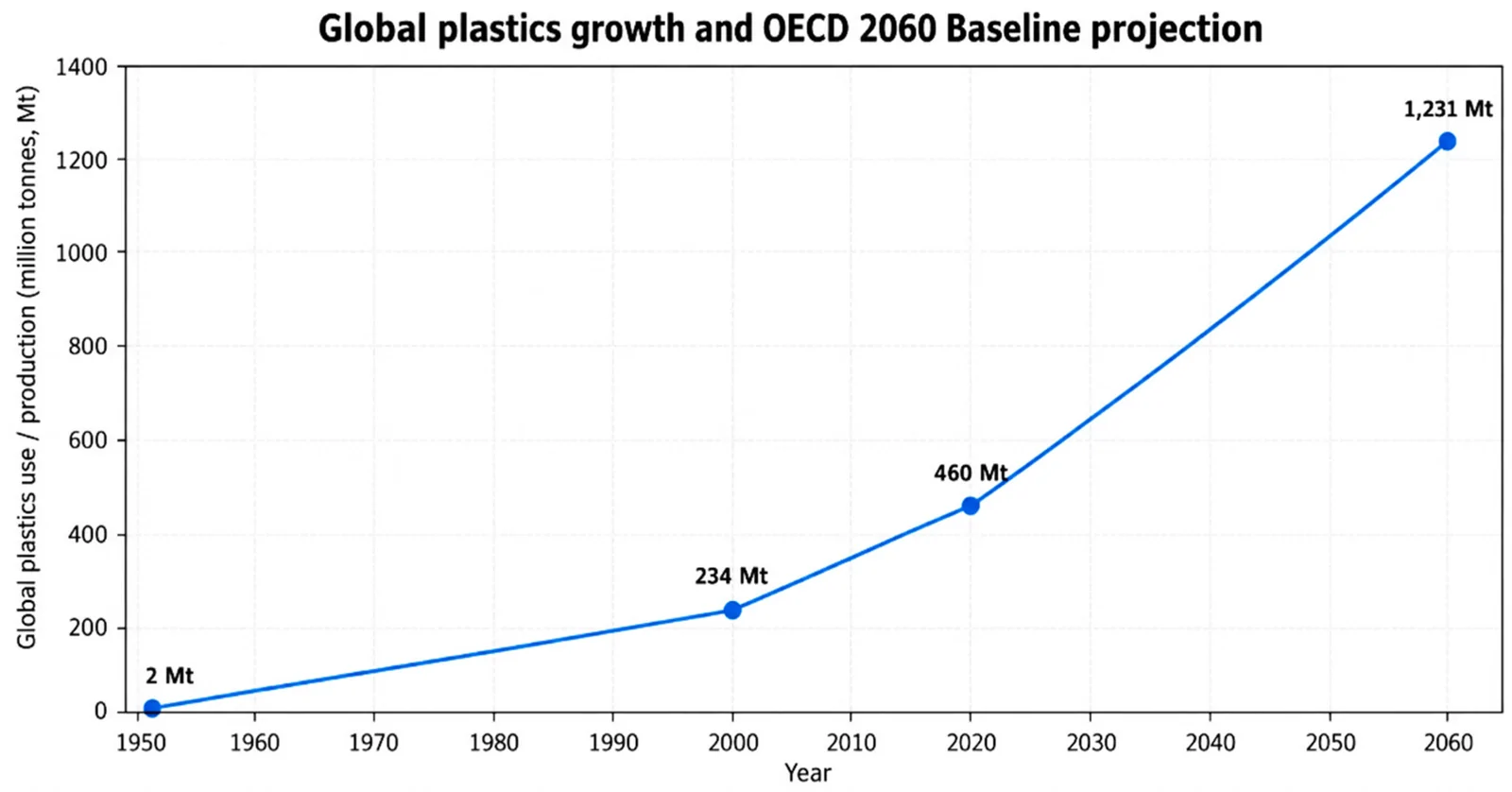
Global plastics production/use growth and OECD 2060 Baseline projection. Values are expressed in million tons (Mt): 2 Mt (1950), 234 Mt (2000), 460 Mt (2019), and 1231 Mt (2060 Baseline) [2].

Since the mid-20th century, plastics have become indispensable materials because of their durability, versatility, low density, and cost efficiency. Global plastics production/use increased from approximately 2 million tons (Mt) in 1950 to 460 Mt in 2019. Under the OECD Baseline scenario, global plastics use is projected to reach approximately 1231 Mt per year by 2060, if additional policies are not implemented [1,2]. Figure 1 summarizes these values using a single unit (Mt).

Short-lived applications are a major source of plastic waste. OECD data indicate that packaging accounts for approximately 40% of global plastic waste, followed by consumer products and textiles, among other applications [2]. These short service lives accelerate the transfer of material from use to the waste-management system and increase the importance of collection, sorting, recycling, and leakage prevention.

The environmental implications of this production model are substantial. In 2019, global plastic waste generation reached approximately 353 Mt; about 82 Mt was mismanaged or littered and an estimated 22 Mt of plastics leaked into the environment [2]. Fragmentation of leaked plastics contributes to persistent macroplastic and microplastic pollution, while the magnitude and pathway of exposure vary strongly by region and waste-management conditions [2,3]. The persistence of polystyrene microplastics in food-chain pathways and their reported toxicological relevance further illustrate why leakage prevention is a system-level priority [4].

**Figure 2:**
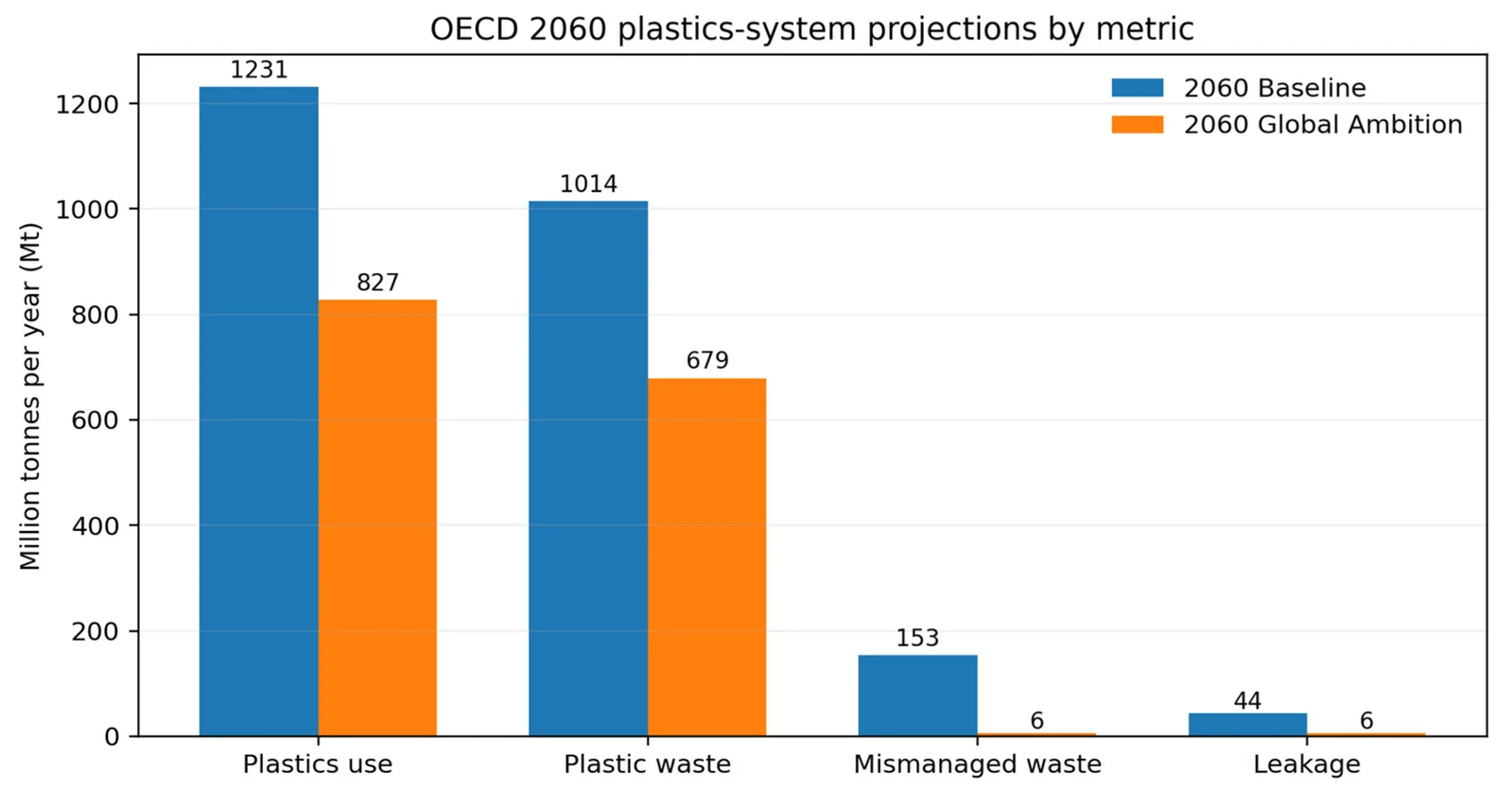
OECD 2060 plastics-system projections shown as separate, non-additive metrics. Baseline: plastics use 1231 Mt, plastic waste 1014 Mt, mismanaged waste 153 Mt, and leakage 44 Mt per year. Global Ambition: plastics use 827 Mt, plastic waste 679 Mt, mismanaged waste 6 Mt, and leakage 6 Mt per year [2]. The metrics are displayed side-by-side because waste, mismanaged waste, and leakage are downstream subsets/flows and must not be summed with plastics use.

Landfilling and incineration remain major end-of-life pathways globally. In 2019, approximately 174 Mt of plastic waste was landfilled and 67 Mt was incinerated, while only a small fraction was ultimately recycled after process losses [2]. The climate and resource implications of plastics, therefore, depend not only on polymer chemistry but also on production energy and end-of-life infrastructure (Figure 2).

Despite these environmental challenges, plastics can also provide functional efficiencies, including lightweighting, durability, and barrier performance. These benefits can reduce burdens in selected applications; however, they must be evaluated against production impacts, service life, and end-of-life outcomes rather than assumed from material choice alone [5,6].

Global recycling remains limited. OECD estimates indicate that only 9% of the 353 Mt of plastic waste generated in 2019 was ultimately recycled after accounting for process losses [2]. This gap motivates coordinated material innovation, improved collection and sorting, better recycling technology, and policy alignment. Previous reviews have examined circular economy recycling systems [7], sustainable-plastics technologies [6], and plastic-waste LCA and management [8], providing the basis for the integrated system-level assessment developed here.

Beyond environmental considerations, the engineering performance of polymer systems must also be evaluated through tribological behavior, including friction, wear resistance, and surface degradation under operational loads. In mechanical systems, material durability is strongly influenced by tribological interactions, which directly affect service life, maintenance requirements, and failure risk. Consequently, sustainability cannot be assessed solely through environmental indicators but must incorporate durability and reliability metrics, as premature material failure may offset environmental gains achieved during production.

### 1.2. Transition Toward Sustainable Polymer Systems

**Figure 3:**
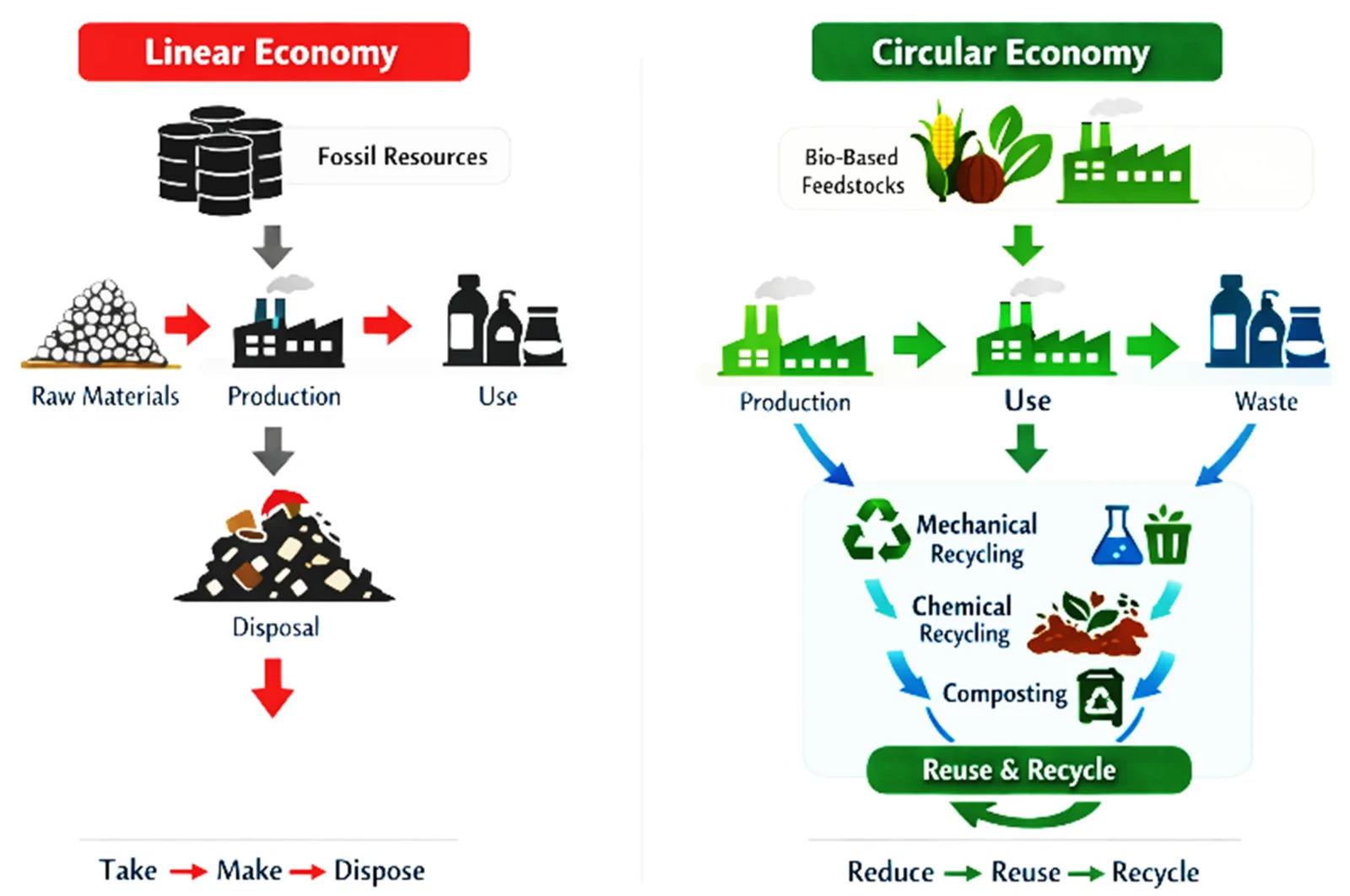
Transition from linear plastic economy to circular sustainable polymer systems. This figure is an original illustration created by the authors for this study.

In response to the environmental pressures associated with conventional plastics, sustainable polymer systems have emerged as a central research and policy priority. Sustainable polymers include bio-based materials derived from renewable feedstocks, recyclable thermoplastics designed for improved circularity, and biodegradable polymers engineered for controlled degradation under specific environmental conditions. These systems aim to reduce fossil resource dependency, lower life cycle emissions, and enhance end-of-life recovery options (Figure 3).

Bio-based polymers, such as polylactic acid (PLA), polyhydroxyalkanoates (PHA), polybutylene succinate (PBS), and polyethylene furanoate (PEF), have attracted significant attention due to their potential to reduce greenhouse gas emissions under optimized production scenarios [9,10]. Advances in green chemistry, renewable feedstock utilization, and process efficiency improvements have accelerated technological development. Simultaneously, mechanical, chemical, and enzymatic recycling technologies are expanding opportunities to reintegrate polymer materials into production cycles.

Using natural waste and incorporating it into composite materials can reduce waste streams while providing sustainable alternatives to conventional materials, resulting in environmentally friendly and cost-effective products. Recent studies demonstrate that agricultural residues, lignocellulosic biomass, and industrial by-products can be effectively valorized as reinforcement phases in polymer composites, improving both mechanical performance and environmental footprint. This approach supports circular economy principles by transforming low-value waste into high-performance engineering materials [11,12]. Animal fibers offer a comparable route: coarse sheep wool, a low-value by-product of sheep farming, has been used at 10 wt% as reinforcement in rigid polyurethane composites, where APTES-treated fibers gave the best tensile performance among the treated variants (30.4 MPa), although none of the reinforced variants fully recovered the strength of the unreinforced matrix [13].

However, sustainability performance remains highly context-dependent. Life cycle assessment (LCA) studies often report reduced carbon footprints for certain bio-based polymers relative to fossil-based counterparts; nevertheless, trade-offs may arise from land-use change, agricultural inputs, energy intensity, infrastructure availability, and system boundary assumptions. Furthermore, global regulatory fragmentation, economic competitiveness challenges, and limited industrial scalability continue to constrain widespread adoption.

A methodological framework for sustainability assessment must integrate both environmental (LCA) and technical performance criteria. A systems approach requires analyzing the entire life cycle, where production energy efficiency and end-of-life recovery options must align with the actual capacities of industrial infrastructure. Without this alignment, material substitution remains only a partial solution. Studies in tribology and materials engineering, such as the review by Gajević et al. on the mechanical properties of aluminum-based metal matrix nanocomposites [14], show that the in-service performance of a substitute material depends strongly on reinforcement type, reinforcement content, and processing route. Sustainability assessment must, therefore, incorporate durability, wear resistance, and failure mechanisms alongside environmental indicators, so that material selection leads to both a reduced environmental impact and reliable long-term performance.

### 1.3. Purpose and Scope of This Review

This study aims to provide a comprehensive and methodologically rigorous evaluation of sustainable polymer systems by integrating:

- A PRISMA-based systematic literature review;
- Bibliometric analysis to identify research trends and thematic clusters;
- Comparative sustainability assessment, including life cycle considerations;
- Analysis of technological, economic, and regulatory barriers;
- Identification of future research and policy directions aligned with circular economy principles.

By synthesizing material innovation, environmental performance data, and industrial implementation challenges, this review seeks to clarify the pathways through which sustainable polymers can transition from emerging alternatives to scalable solutions. The findings are positioned within the broader context of circular economy strategies and global sustainability objectives, including Sustainable Development Goals 12 (Responsible Consumption and Production), 13 (Climate Action), and 14 (Life Below Water).

Recent reviews provide important but differently bounded perspectives. Lazăr et al. [15] address eco-design of polymer-matrix composites; Shamsuyeva and Endres [7] focus on circular economy recycling systems; Mushtaq et al. [6] review technological developments and applications; Rosenboom et al. [16] discuss bioplastics in a circular economy context; Ali et al. [17] synthesize bioplastic production and LCA; Jiao et al. [8] review plastic-waste LCA and management; and Velasquez et al. [18] integrate chemistry, societal perspectives, environmental implications, and end-of-life considerations. Table 1, therefore, compares the present review with the most closely related review scopes rather than asserting novelty from topic breadth alone.

The contribution of the present review is methodological integration. It combines a PRISMA-screened dataset (*n* = 193) with (i) a bibliometric evidence-gap diagnostic that compares thematic salience with implementation-relevant evidence coverage, (ii) a source-linked and boundary-stratified LCA synthesis, (iii) a polymer-by-end-of-life infrastructure matrix that shows how performance changes across recovery and leakage archetypes, (iv) semi-quantitative TEA/maturity interpretation, and (v) conclusion-level confidence grading. The novelty claim is, therefore, restricted to this linked evidence architecture and to its use as a system-dependence diagnostic; it is not a claim that earlier reviews failed to address individual components such as LCA, recycling, or biopolymer chemistry.

## 2. Materials and Methods

**Figure 4:**
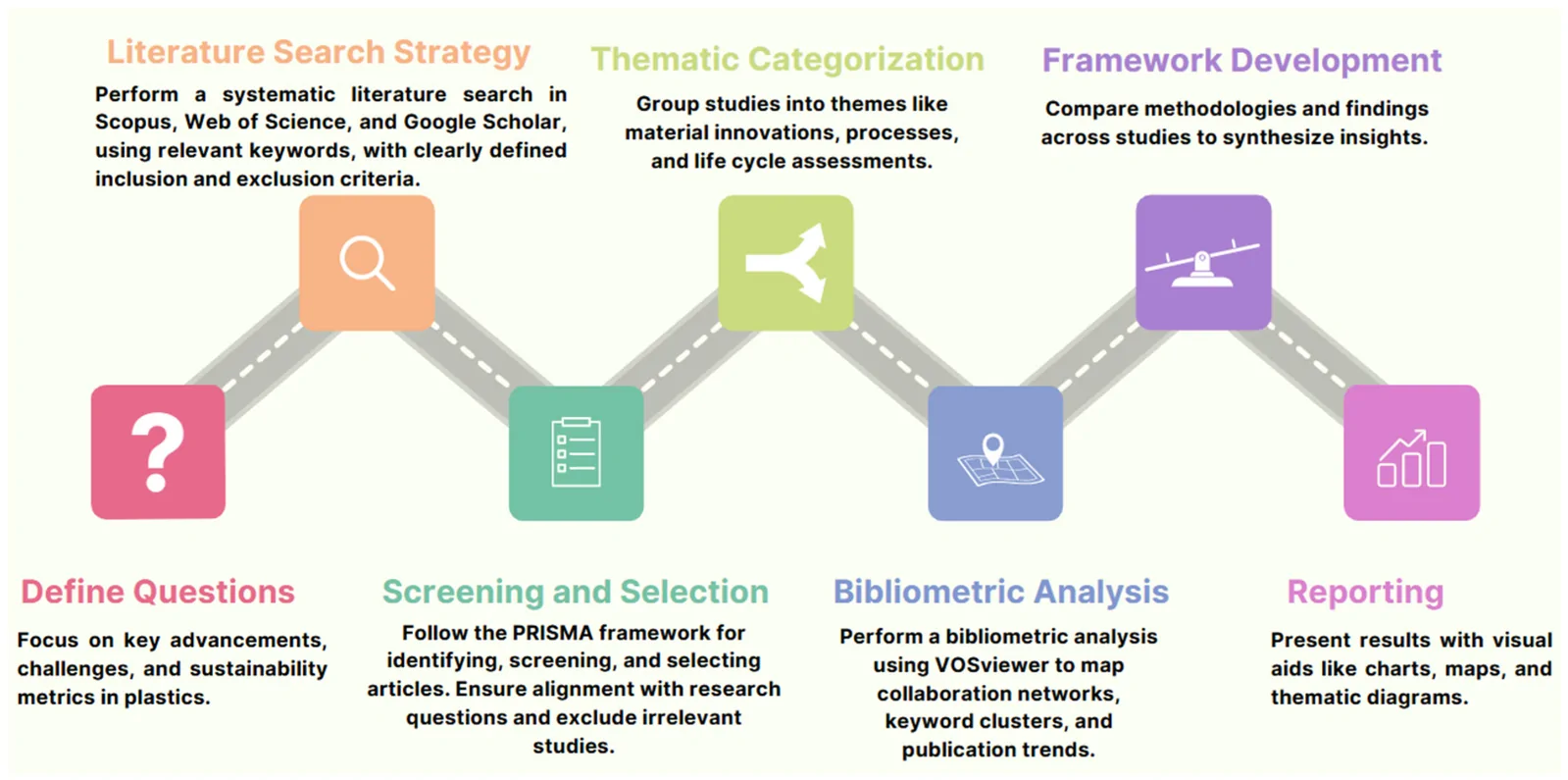
Seven-point step roadmap for materials and methods in sustainable plastics review. This figure is an original illustration created by the authors for this study.

This section describes the methodological framework used to identify, screen, code, and synthesize evidence on sustainable polymer systems. Figure 4 summarizes the seven-stage workflow, from research-question definition and database searching to eligibility screening, bibliometric mapping, evidence extraction, LCA/TEA interpretation, and system-level synthesis.

Each of the seven methodological steps presented in Figure 4 represents a critical stage in ensuring analytical rigor: definition of research questions guiding scope and criteria; systematic database selection ensuring coverage and quality; keyword and search string development for reproducibility; application of inclusion/exclusion criteria; PRISMA-based screening and validation; bibliometric mapping of thematic structures; and synthesis through source-linked quantitative comparison and explicit evidence coding.

This structured workflow ensures traceability, reduces selection bias, and enables reproducible comparison across heterogeneous sustainability studies.

### 2.1. Research Questions

The review was structured around seven research questions spanning terminology and classification, material innovation, manufacturing, life cycle performance, scalability, limitations, and future system-level pathways:

- Background and Definitions:

How are sustainable plastics, bioplastics, biodegradable polymers, and recyclable polymers currently defined, and which criteria are used to assess their sustainability (e.g., carbon footprint, circularity, biodegradation conditions, and recyclability)?

- Material Innovations:

What are the main recent developments in biopolymers, renewable-feedstock plastics (including PLA and PHA), and hybrid or composite polymer systems?

- Manufacturing Processes:

Which manufacturing technologies and process adaptations can reduce energy use, material losses, solvent demand, and emissions, while preserving functional performance?

- Life Cycle Assessment (LCA):

How do LCA results for sustainable polymer systems compare with conventional alternatives, and how sensitive are those comparisons to functional-unit selection, system boundaries, allocation methods, energy assumptions, and end-of-life scenarios?

- Scalability and Adoption:

Which technical, industrial, economic, consumer, and regulatory factors determine whether promising sustainable-polymer technologies can progress from laboratory feasibility to scalable deployment?

- Challenges and Limitations:

What technical, economic, and environmental constraints limit progress in sustainable polymer systems, and which mitigation strategies are supported by the reviewed evidence?

- Future Directions:

Which research, engineering, infrastructure, and policy priorities are most important for accelerating the transition toward circular and verifiable sustainable-polymer systems?

Together, these questions define the analytical scope of the review and ensure that material chemistry is interpreted together with processing, functional performance, end-of-life pathways, infrastructure, and economic feasibility, rather than as an isolated sustainability attribute.

### 2.2. Literature Search Methodology

The literature-search protocol comprised database selection, controlled keyword development, Boolean query construction, duplicate removal, eligibility screening, and evidence coding. The search logic was kept consistent across databases while database-specific field syntax was used where applicable.

#### 2.2.1. Databases and Sources

A structured search was conducted in four complementary databases:

- Scopus: Journals and proceedings of peer-reviewed nature.
- Web of Science: for high-impact factor journals and citation analysis;
- Google Scholar was used as a supplementary search tool; records were retrieved with a dedicated retrieval tool that overcomes the 1000-result display limit of the web interface (38,713 records), and only peer-reviewed studies meeting inclusion criteria were retained;
- PubMed: To obtain research on bioplastics, focusing on environmental health impacts.

#### 2.2.2. Search Strings and Keywords

The thematic search blocks were constructed with explicit Boolean grouping to avoid ambiguity in operator precedence. The core expressions were:

- “sustainable plastics” OR “sustainable polymers”

(Section: Background and Definitions);

- (“biopolymers” OR “green polymers” OR “natural-based plastics”) AND “renewable resources”

(Section: Material Innovations);

- (“green manufacturing” OR “sustainable manufacturing”) AND (“polymers” OR “plastics”)

(Section: Manufacturing Processes);

- (“life cycle assessment” OR “LCA”) AND (“environmental impact” OR “economic viability”) AND “sustainable polymers”

(Section: Life Cycle Assessment);

- (“scalability” OR “adoption barriers”) AND “industrial integration” AND “sustainable plastics”

(Section: Scalability and Adoption);

- (“challenges” OR “limitations”) AND “sustainable plastics”

(Section: Challenges and Limitations);

- “circular economy” AND “innovations” AND “sustainable polymers”

(Section: Future Directions).

For reproducibility, the search protocol is reported at database level. The systematic search window was 1998–2024 and the final systematic search was completed in July 2025. The exact calendar day was not preserved in the original export log; this limitation is stated explicitly rather than reconstructed retrospectively. Scopus was searched in TITLE-ABS-KEY fields, Web of Science Core Collection in Topic (TS), PubMed in Title/Abstract, and Google Scholar as a supplementary full-text discovery source using the same seven thematic concept blocks. The database-specific strings, fields, retrieved-record counts, and deduplication rules are reproduced in Appendix A (Table A1). The per-database counts are Scopus *n* = 39,672; Web of Science *n* = 34,116; Google Scholar *n* = 38,713; and PubMed *n* = 8947, giving the reported identification total of *n* = 121,448.

Because the systematic search window was defined as 1998–2024, publications appearing after the cutoff were not retroactively added to the PRISMA count, bibliometric dataset, or quantitative synthesis dataset. Three post-cutoff sources—Koçak Mutlu et al. (2025) [19], Karahan and Iftekhar (2026) [20], and Velasquez et al. (2025) [18]—were incorporated as targeted contextual updates and are explicitly treated as supplementary evidence outside the *n* = 193 systematic dataset. Two further post-cutoff studies, on sheep-wool-fiber-reinforced rigid polyurethane composites [13] and on interlocking geometric interfaces for localized reinforcement [21], were included using the same approach.

#### 2.2.3. Inclusion and Exclusion Criteria

Eligibility criteria were defined before full-text screening. The review retained English-language peer-reviewed studies published between 1998 and 2024 when they reported transparent methods and evidence relevant to environmental performance, circularity, biodegradation, recyclability, manufacturing, techno-economics, or infrastructure. Studies outside the time window, non-peer-reviewed opinion material, and studies lacking methodological transparency or transferable sustainability evidence were excluded (Table 2).

Because system dependence is partly regional, region-specific studies were not treated as inherently non-generalizable. They were retained when they reported transferable variables such as electricity mix, recycling or composting availability, collection rates, feedstock assumptions, or industrial scale conditions. Such studies were coded as context-specific evidence rather than removed from the review. Full peer-reviewed conference proceedings were also retained when methodological transparency was sufficient, because engineering and industrial scale-up evidence is frequently reported first in applied venues. The exclusion criterion was, therefore, applied to studies lacking transferable parameters or methodological detail, not to regional evidence per se.

To evaluate potential selection bias, each included record was assigned a qualitative context flag: global/general, region-specific transferable, or region-specific non-transferable. Only the first two categories were included in the qualitative synthesis, while quantitative aggregation was restricted to studies with comparable functional units and transparent assumptions. This procedure reduces the risk of biasing the dataset toward idealized global studies while preserving methodological comparability.

#### 2.2.4. Screening Process

PRISMA 2020 was used as the reporting framework for identification, screening, eligibility, and inclusion; it was not treated as a substitute for the review methodology. Duplicate removal was performed before title–abstract screening in two stages: (i) exact matching of DOI, PMID or other persistent identifiers when available and (ii) normalized matching of title, first author and publication year after lower-casing, punctuation removal and whitespace normalization. Potential near duplicates were manually adjudicated. Two reviewers independently screened titles/abstracts and full texts. Disagreements were discussed until consensus was reached; unresolved cases were retained for the next screening stage to minimize false exclusion. A numerical inter-reviewer agreement coefficient (e.g., Cohen’s κ) was not prospectively recorded and is therefore not reported retrospectively. Bibliometric analysis was subsequently performed using Bibliometrix (version 5.3.0, stable release) and VOSviewer (version 1.6.20), and was interpreted as evidence of research attention and gaps, rather than as evidence of technological superiority.

A citation-to-claim audit was performed to verify the correspondence between in-text claims and the cited sources. Each in-text citation was rechecked against the title, abstract, and, where available, the full text of the cited source. Quantitative claims were retained only when the cited work reported the corresponding value, range, or directly relevant methodological result. Citations that were only tangentially related were removed from the claim or the statement was rewritten to match the actual scope of the source. This audit was applied particularly to global plastics-flow data, cost claims, biodegradation statements, policy statements, and quantitative LCA comparisons.

The selection process comprised four reporting stages—identification, screening, eligibility, and inclusion—while methodological quality and comparability were assessed separately through the criteria in Table 2. The completed PRISMA 2020 checklist is supplied as For transparency and bibliographic traceability, Supplementary Table S1 provides an inventory of the 103 references cited in the manuscript, distinguishing publications within the systematic search period from post-cutoff contextual sources. Eligibility based on publication year is not considered evidence of inclusion in the final dataset of 193 studies.

**Figure 5:**
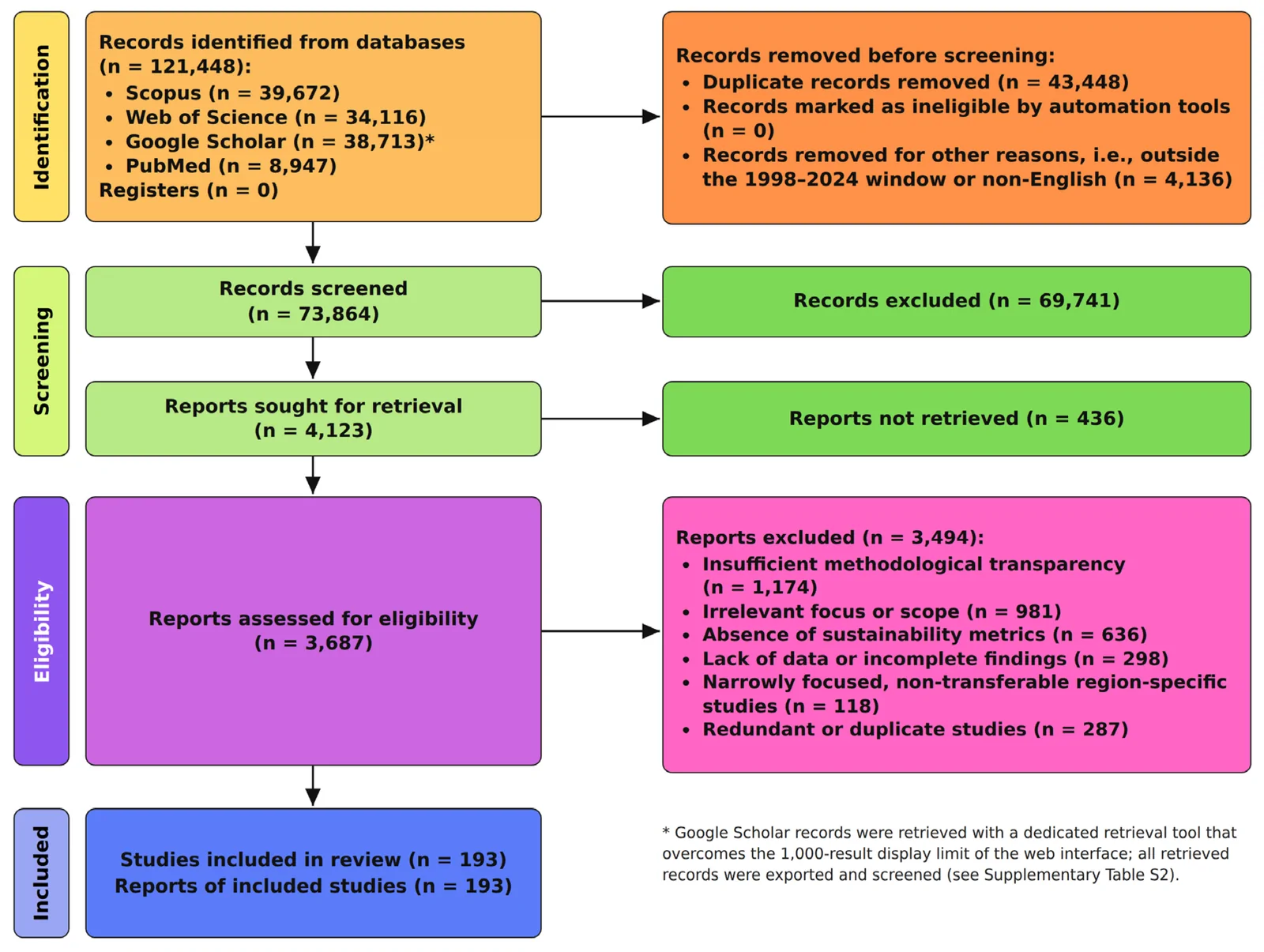
PRISMA flow diagram for the systematic article selection process.

Figure 5 reports the flow of records through the review, including exclusions at each stage and the final set of 193 included studies.

To address variability across studies with different system boundaries (e.g., cradle-to-gate vs. cradle-to-grave), data were harmonized to a common functional unit (1 kg polymer at factory gate). Outliers were identified based on interquartile range (IQR) criteria and retained only if supported by methodological transparency; otherwise, they were excluded from aggregation.

Uncertainty of aggregated indicators was estimated using the coefficient of variation:

(1) $${\mathrm{CV}}_{k}=\frac{\sigma_{k}}{{µ}_{k}}$$

where CV_k_ represents variability for criterion k, σ_k_ is the standard deviation, and μ_k_ is the mean value. This metric provides a normalized measure of dispersion across heterogeneous datasets.

Because the extracted LCA values were often skewed and methodologically heterogeneous, median values were used as descriptive central estimates within comparable methodological strata, whereas the coefficient of variation was applied only to describe variability across studies.

To complement the LCA synthesis, techno-economic information reported in the eligible literature was extracted whenever available, including indicative production cost ranges, relative cost multiples versus conventional polymers, capital intensity constraints, and scale-up limitations. Because the available TEA evidence was heterogeneous and reported using non-uniform assumptions, these data were not pooled through formal meta-analysis. Instead, they were used as contextual comparative indicators to interpret whether environmentally favorable polymers are also compatible with current industrial and market conditions.

The diagram provides a visual summary of the study-selection flow reported according to PRISMA 2020. Methodological quality, evidence comparability, and quantitative synthesis are evaluated separately through the procedures described in Section 2.2 and Section 2.4.

The key stages in the selection process are outlined below:

- Identification: A total of 121,448 records were retrieved from four primary databases (Scopus, *n* = 39,672; Web of Science, *n* = 34,116; Google Scholar, *n* = 38,713; PubMed, *n* = 8947) using carefully curated search strings. Records were removed prior to screening due to duplication (*n* = 43,448) or for not meeting predefined temporal or language criteria (*n* = 4136);
- Screening: Following deduplication, 73,864 records were screened based on titles and abstracts. At this stage, 69,741 records were excluded due to irrelevance or failure to meet inclusion criteria;
- Eligibility: Of the 4123 reports sought for full-text retrieval, 436 reports were not retrieved. Therefore, 3687 full-text reports were assessed for eligibility (4123 − 436 = 3687). A total of 3494 reports were excluded at this stage: insufficient methodological transparency (*n* = 1174), irrelevant focus or scope (*n* = 981), absence of sustainability metrics (*n* = 636), lack of data or incomplete findings (*n* = 298), narrowly focused non-transferable region-specific studies (*n* = 118), and redundant or duplicate studies identified during full-text assessment (*n* = 287);
- Inclusion: The final set comprised 193 studies (3687 assessed minus 3494 excluded), which were included in the thematic analysis and bibliometric evaluation.

Figure 5 documents PRISMA-based reporting of the study flow. Methodological rigor is supported separately by the pre-defined eligibility criteria, duplicate removal, independent screening, transparent harmonization rules, and explicit treatment of uncertainty and evidence strength.

The checklist details the correspondence between each PRISMA reporting item and the sections of this manuscript. Although no formal protocol registration (e.g., PROSPERO) was performed due to the engineering-focused scope of this review, the methodology followed predefined eligibility criteria, structured screening procedures, and reproducible data extraction steps, in accordance with PRISMA recommendations.

### 2.3. Bibliometric Analysis

The bibliometric analysis aimed to explore the evolution of research in sustainable plastics, identify influential contributions, and map key thematic areas and collaborations. This analysis offers insights into the state of the field and emerging trends, with a focus on advancing sustainability in polymer science.

Bibliometrix and VOSviewer were used to characterize publication trends, citation structure, keyword co-occurrence, and collaboration patterns in the 1998–2024 dataset. Bibliometric outputs were used to identify thematic concentration and evidence gaps; citation frequency or cluster prominence was not interpreted as a measure of technological maturity or environmental performance.

**Figure 6:**
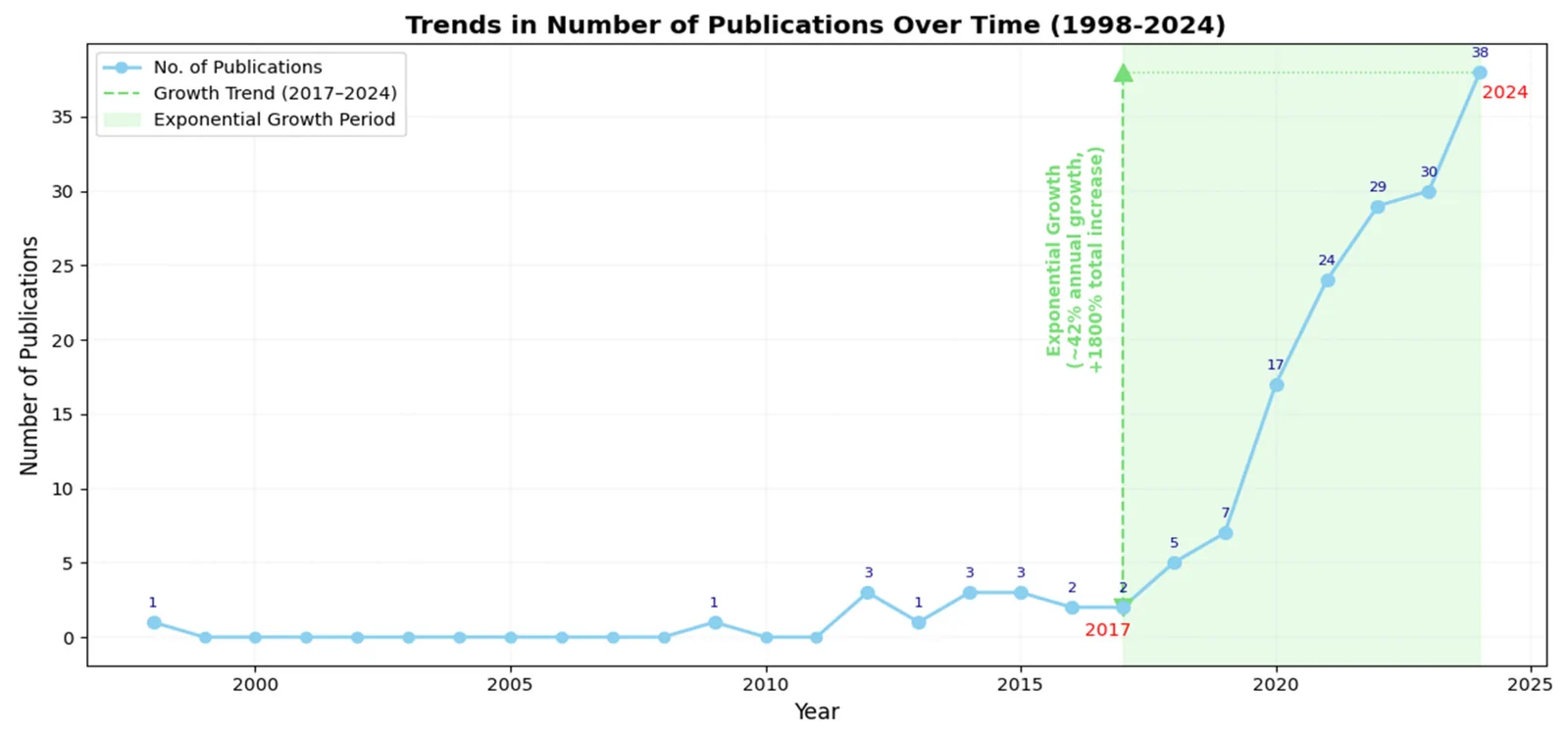
Trends in the annual number of publications related to sustainable plastics (1998–2024).

Figure 6 below illustrates the rapid growth in publications on sustainable plastics, particularly after 2017, reflecting heightened global interest.

Publication activity was limited before 2012 and increased markedly after 2017. This growth indicates rapidly expanding research attention to sustainable polymers, but it does not by itself demonstrate technological maturity or environmental superiority. The engineering relevance of the trend is, therefore, evaluated through the evidence-mapping procedure in Section 2.4.

**Figure 7:**
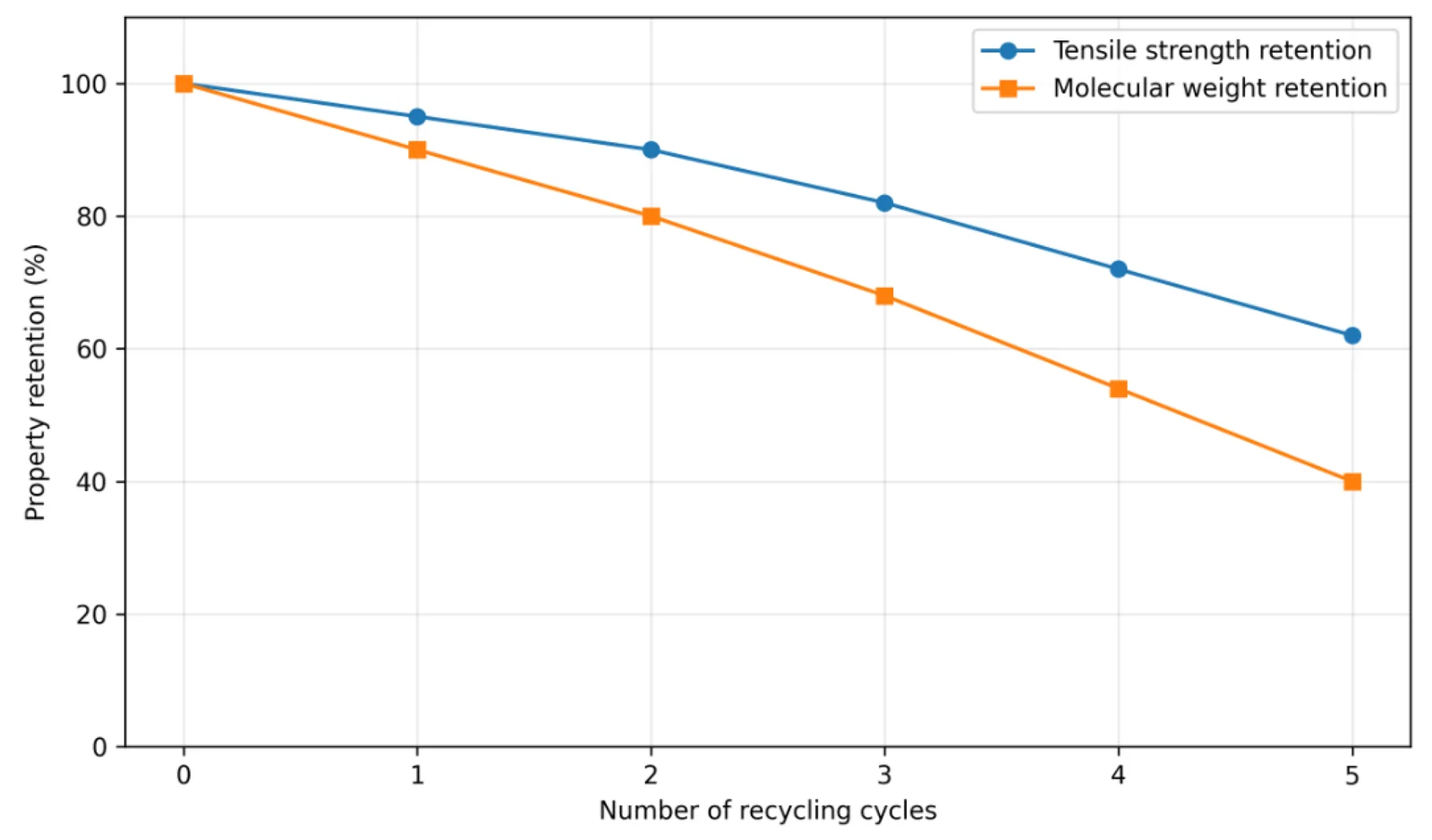
Conceptual schematic of degradation mechanisms associated with repeated mechanical recycling. The figure summarizes general mechanisms reported in the mechanical-recycling literature [7,8,22] and is not a fitted or universal quantitative curve for a specific polymer.

Figure 7 is a conceptual schematic of degradation mechanisms associated with repeated mechanical reprocessing of thermoplastics. The literature reports polymer- and process-dependent changes arising from thermo-mechanical history, including chain scission, oxidation, molecular-weight changes, and deterioration or redistribution of tensile and elongation properties. Because the magnitude and direction of these changes depend on polymer grade, additives, processing temperature, residence time, and the number of reprocessing cycles, the figure is not intended as a universal quantitative degradation curve for a specific polymer. It summarizes the general engineering mechanisms discussed in the mechanical-recycling literature [7,8,22]; polymer-specific quantitative conclusions should be taken from the primary studies underlying those reviews.

**Figure 8:**
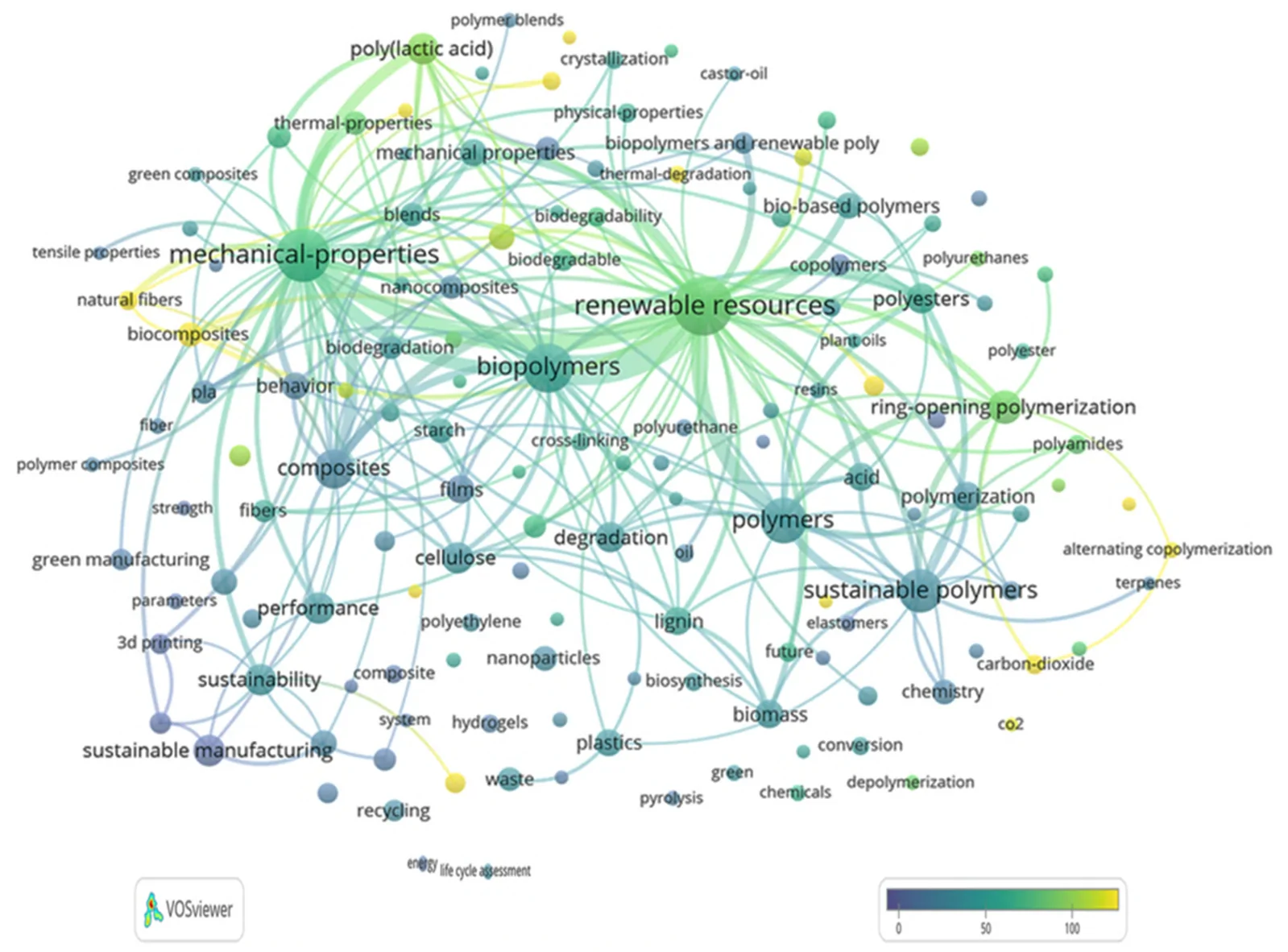
Keyword co-occurrence network based on citation scores in sustainable plastics research. This figure is an original illustration created by the authors for this study.

The image below, in Figure 8, entitled “Keyword co-occurrence network based on citation scores in sustainable plastics research”, gives a clear picture of the weighted significance of research issues through citation scores.

Created with VOSviewer, the network employs settings including “LinLog/Modularity” normalization, “Occurrences” as the weighting factor, and “Average Citation Score” to determine node size and color. The clusters reveal the ranking of key themes: renewable resources, mechanical properties, biopolymers, and sustainable polymers. The “renewable resources” cluster, identified by its large green nodes, reflects the emphasis on bio-based materials like PLA, lignocellulosic biomass, and biopolymers. The “mechanical properties” cluster includes topics such as strength and durability improvements in sustainable plastics, while the “biopolymers” and “sustainable polymers” clusters highlight materials and processes central to the field. Node sizes correspond to average citation scores, indicating the relative impact of topics within the network. The inclusion of citation scores highlights not only the thematic importance but also the influence and recognition these topics have garnered in the academic community. The dense connections in the network emphasize the interdisciplinary nature of the field, integrating advances in materials science, green chemistry, and manufacturing technologies.

Mechanical recycling leads to the progressive degradation of polymer chains due to thermo-mechanical stress, resulting in molecular-weight reduction and the deterioration of mechanical properties. Typically, tensile strength and elongation at break decrease significantly after 3–5 recycling cycles, depending on polymer type and processing conditions. This relationship highlights the importance of stabilizers and controlled processing to extend material lifespan.

**Figure 9:**
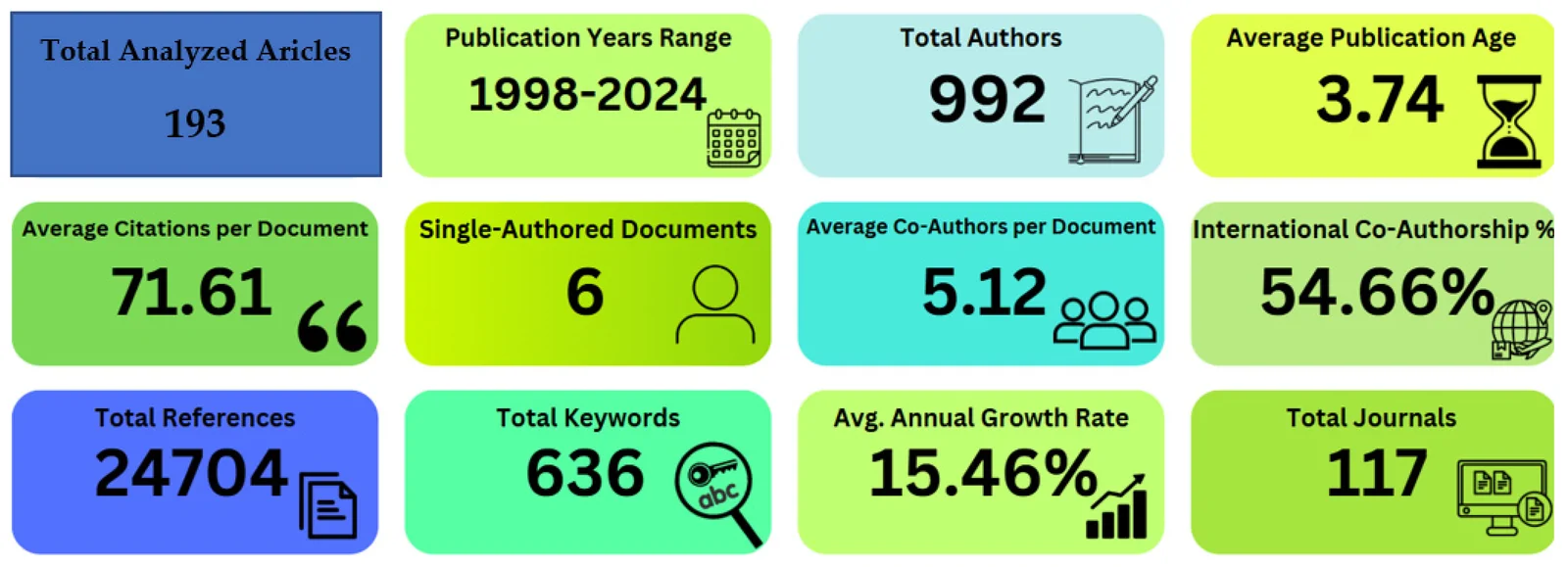
Overview of bibliometric analysis metrics in sustainable plastics research.

Figure 9, entitled “Overview of bibliometric analysis metrics in sustainable plastics research”, provides a comprehensive summary of the key quantitative metrics derived from the bibliometric study.

The research covers 193 articles published between 1998 and 2024, written by 992 authors, with an average of 5.12 co-authors per document. The dataset shows an average of 71.61 citations per document and a total of 24,704 references. The study finds that more than half (54.66%) of the publications have international co-authorship, indicating global collaboration in this field. The data also reveal an annual growth rate of 15.02%, highlighting the rapidly expanding interest in sustainable plastics research. These figures give a quantitative basis for understanding the evolution and collaborative dynamics for this rapidly growing field.

The bibliometric results show rapid growth in research output and strong visibility of renewable resources, biopolymers, and mechanical-property optimization. To avoid conflating scientific attention with implementation readiness, these clusters were cross-referenced with quantitative LCA, techno-economic, infrastructure, end-of-life, and engineering-performance evidence in Section 2.4. Bibliometrics, therefore, functions here as a diagnostic tool for research concentration and gaps, not as direct evidence of sustainability performance.

To enhance interpretative value, bibliometric findings were cross-referenced with engineering, LCA, TEA, and end-of-life evidence categories using the evidence mapping approach described in Section 2.4. The dominant clusters related to renewable resources, mechanical properties, biopolymers, and sustainable polymers were compared with the availability of quantitative LCA data, explicit infrastructure modeling, and economic feasibility indicators. This enabled the bibliometric analysis to function as a diagnostic tool for evidence imbalance rather than only as a descriptive map of publication activity.

Research misalignment was, therefore, defined as high bibliometric visibility of a topic combined with low implementation-relevant evidence coverage. Using this definition, infrastructure compatibility, end-of-life scenario modeling, and techno-economic scalability appear underrepresented relative to material innovation and mechanical-property optimization. This result provides a structured basis for the subsequent conclusion that polymer sustainability is system-dependent.

### 2.4. Integrated Evidence Mapping and Confidence Grading

To strengthen methodological integration, the bibliometric, LCA, techno-economic, and engineering evidence streams were not interpreted as independent outputs. Instead, they were linked through an evidence mapping procedure. First, keyword co-occurrence clusters were grouped into system-level evidence categories: material innovation; production and processing; LCA impact assessment; end-of-life infrastructure; techno-economic feasibility; and policy or governance. Second, each of the 193 included studies was coded according to whether it provided quantitative LCA data, explicit end-of-life modeling, techno-economic indicators, infrastructure or regional implementation evidence, and mechanical or engineering performance data.

Research-system misalignment was operationally defined as a condition in which a bibliometric cluster showed high thematic salience but low corresponding coverage in applied evidence categories required for implementation. In practical terms, a theme was classified as under-supported when it was highly visible in the keyword network but was associated with limited quantitative LCA harmonization, limited TEA evidence, or limited scenario-based end-of-life modeling. This approach links bibliometric structure directly to evidence gaps rather than using bibliometrics only as a descriptive background analysis.

The evidence gap interpretation followed three levels: low gap, when bibliometric salience and applied evidence coverage were broadly aligned; moderate gap, when evidence existed but was fragmented across methods or regions; and high gap, when a theme was bibliometrically prominent but weakly supported by quantitative LCA, TEA, or infrastructure data (Table 3). Confidence levels reported in the Discussion were then assigned based on data consistency, methodological comparability, number of studies, and the sensitivity of conclusions to regional assumptions.

Quantitative coding of the 193 included records confirms the bibliometric-evidence gap: approximately 64% addressed material innovation or mechanical/processing performance, 47% reported quantitative LCA indicators (kg CO_2_e/kg or equivalent), only 19% included explicit techno-economic data (production cost, CAPEX, learning rate), and only 14% provided scenario-based end-of-life modeling with infrastructure assumptions. This asymmetry, whereby high research salience for material innovation versus low coverage of implementation-relevant evidence, operationalizes the misalignment claim and provides the structural basis for the system-dependent conclusion.

This evidence-linkage matrix provides the structural bridge between the bibliometric mapping and the subsequent LCA, TEA, and end-of-life synthesis. It shows that the paper’s system-dependent conclusion is supported not only by individual LCA ranges but also by the mismatch between highly visible research themes and the evidence required for implementation.

**Figure 10:**
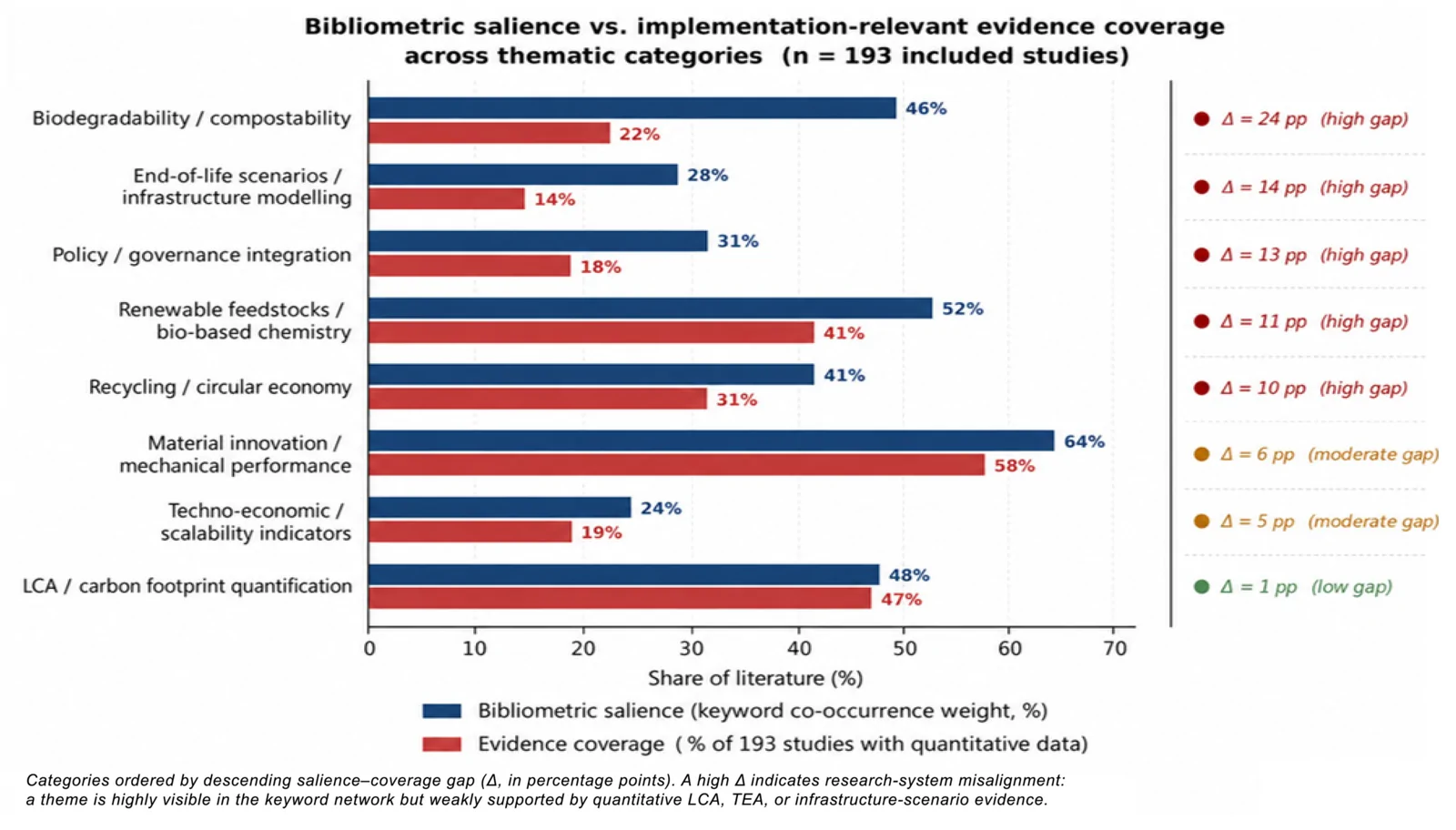
Quantitative comparison between bibliometric salience and implementation-relevant evidence coverage across thematic categories (*n* = 193 included studies).

Figure 10 extends this quantification across all eight thematic categories identified in the keyword co-occurrence network and visualizes the salience–coverage differential (Δ, in percentage points) for each. Three tiers of misalignment can be distinguished. A first tier of high gaps (Δ ≥ 8 pp) is observed for biodegradability and compostability (Δ = 24 pp), end-of-life infrastructure modelling (Δ = 14 pp), policy and governance integration (Δ = 13 pp), renewable feedstocks (Δ = 11 pp), and recycling within circular economy framings (Δ = 10 pp). In each of these categories, thematic visibility in the literature substantially exceeds the share of studies that report quantitative, implementation-relevant data. A second, moderate tier (Δ = 4–7 pp) covers material innovation and mechanical performance, as well as techno-economic indicators, where coverage is closer to thematic salience but remains incomplete. A third tier of substantive alignment is observed only for LCA and carbon-footprint quantification (Δ = 1 pp), where bibliometric weight and quantitative coverage are essentially balanced. This non-uniform distribution is methodologically important: the largest gaps are concentrated precisely in the dimensions that determine real-world sustainability outcomes, like composting and recycling infrastructure, regional policy alignment, and economic scalability, while a category that does not directly drive implementation decisions (LCA reporting itself) is well covered. The system-dependent conclusion is, therefore, not an interpretive overlay imposed on the data, but a structural feature of the evidence base, made visible once bibliometric salience and quantitative coverage are placed on the same scale.

## 3. Results

### 3.1. Background and Definitions

- What Are Sustainable Plastics?

In this review, “sustainable plastics” is used as a systems-oriented umbrella term rather than as a single material class. It encompasses polymer systems designed to reduce life cycle burdens through renewable feedstocks, longer functional life, reduced-impact manufacturing, effective reuse or recycling, or controlled biodegradation under specified conditions. Bio-based origin, recyclability, and biodegradability are treated as distinct attributes rather than interchangeable definitions of sustainability [16,23,24].

#### 3.1.1. Bioplastics

Bioplastics include materials that are bio-based, biodegradable, or both; these attributes must be distinguished because renewable feedstock origin does not automatically imply biodegradability or lower life cycle impact. Common renewable feedstocks include starch, sugar-derived intermediates, and cellulose [10,16]:

Bio-based non-biodegradable plastics, such as bio-polyethylene (bio-PE), bio-polypropylene (bio-PP), and bio-polyethylene terephthalate (bio-PET), which exhibit similar physical and mechanical properties to their fossil-based counterparts but are produced from renewable sources [16,17,23,25];

Bio-based biodegradable plastics, including polylactic acid (PLA) and polyhydroxyalkanoates (PHA), which are designed to decompose under specific environmental conditions, such as industrial composting or enzymatic degradation [17].

Despite their advantages, the sustainability of bioplastics is contingent on factors such as agricultural land use, potential food crop competition, and the effectiveness of waste management systems in processing these materials [9,10].

#### 3.1.2. Recyclable Polymers

Recyclable polymers are materials that can be recovered and reprocessed through technically and economically viable pathways while retaining sufficient value for subsequent use. Recyclability depends not only on polymer chemistry but also on collection, sorting, contamination, additive formulation, reprocessing history, and market demand for the recovered resin.

- Conventional thermoplastics such as polyethylene terephthalate (PET), high-density polyethylene (HDPE), and polypropylene (PP) benefit from mature processing and recovery infrastructure in many markets, although actual recycling performance remains strongly dependent on collection and sorting quality [7,22].
- Advanced recyclable materials, such as polyethylene furanoate (PEF), a bio-based alternative to PET, are characterized with better gas barrier and improved recyclability.
- Emerging reprocessable thermoset systems such as vitrimers use dynamic covalent networks to enable reshaping, repair, or reprocessing. Their sustainability potential therefore derives from possible lifetime extension and material recovery rather than from feedstock origin alone [23,26].

Mechanical recycling remains the dominant recovery route for thermoplastics, while chemical recycling can recover monomers or intermediates from streams that are difficult to recycle mechanically. Both routes are constrained by contamination, sorting efficiency, yield losses, and energy demand; consequently, the preferred pathway must be evaluated through material-specific and region-specific life cycle scenarios [7,8,22].

#### 3.1.3. Biodegradable Plastics

Biodegradable plastics are polymers for which microbial conversion can occur under specified environmental conditions and within a defined time frame. Depending on the standard and environment, degradation products may include carbon dioxide (or methane under anaerobic conditions), water, biomass, and mineral products. Biodegradability is independent of whether the carbon feedstock is bio-based or fossil-derived [27,28]. The degradation of natural polymers is, likewise, conditioned by their chemical environment; in archaeological wood treated with alum, for example, the conservation treatment itself was found to influence the degradation of the cellulose and lignin fractions [29].

- Petroleum-based biodegradable plastics, for example, polybutylene adipate terephthalate (PBAT), are usually mixed with bio-based parts (like PLA) to get better flexibility and faster decomposition.
- Bio-based biodegradable plastics, as polybutylene succinate (PBS) and starch-based blends, are applied in such areas as agricultural mulch films and disposable food ware.
- Certain PHA-family materials, including PHB, have shown biodegradation under selected marine test conditions; however, marine degradability must not be interpreted as permission for environmental release or as a substitute for waste prevention and collection. Rates depend on material formulation, specimen geometry, temperature, microbial community, and test method [27,28,30].

Biodegradation is explicitly environment-dependent. Temperature, moisture, oxygen availability, microbial activity, specimen thickness, and residence time can substantially change the degradation rate. Materials certified for industrial composting may not degrade adequately in home compost, soil, freshwater, marine environments, or landfill; incomplete degradation can therefore remain a persistence or microplastic concern [27,28,31].

A critical distinction must be made between industrial compostability and home compostability. Industrial composting requires controlled conditions (temperatures of 50–70 °C, regulated humidity, and microbial activity), under which materials such as PLA can degrade efficiently. In contrast, home composting occurs at lower and less stable temperatures, where many industrially compostable plastics fail to fully degrade. This distinction is essential for avoiding consumer misinterpretation and improper disposal.

#### 3.1.4. Metrics for Sustainability

The classification of sustainable plastics is further assessed through key sustainability metrics, including [8,24,32], as follows:

- Carbon footprint: Life cycle emissions associated with production, use, and disposal;
- Biodegradability: The rate and conditions under which a plastic material decomposes;
- Recyclability: The ability to recover and reprocess materials without significant loss of functionality [33];
- Resource efficiency: The extent to which raw materials, energy, and water are optimized in production and disposal processes [34].

#### 3.1.5. Determination of Sustainability Metrics

To avoid conceptual ambiguity, materials are evaluated according to their primary functional role within specific application contexts. Bio-based, biodegradable, and recyclable polymers represent overlapping but distinct categories, and their sustainability performance is assessed relative to intended use (e.g., short-life packaging vs. durable goods).

This application-specific perspective is essential to avoid misleading comparisons, as materials optimized for closed-loop recycling systems may not be suitable for environments characterized by leakage or mismanagement, where biodegradability may offer advantages.

The determination of sustainability metrics involves a combination of experimental testing, life cycle assessment (LCA) methodologies, and regulatory compliance standards:

- Carbon footprint is quantified using LCA methodologies, which evaluate the total greenhouse gas emissions (CO_2_-equivalent) across the entire life cycle of a plastic material, from raw material extraction to end-of-life disposal.
- Biodegradability is determined through standardized testing methods such as ASTM D5338 (compostability under aerobic conditions) [35], ISO 14855 (biodegradation in controlled environments) [36], and marine degradation assessments to evaluate the decomposition of plastics in aquatic ecosystems [26,32].
- Recyclability is assessed based on the ability of a polymer to undergo mechanical or chemical recycling without significant loss of material properties, measured through techniques like melt flow index analysis, thermal stability tests, and polymer-chain integrity assessments.
- Resource efficiency is evaluated through material flow analysis (MFA) and process optimization studies, examining raw material utilization rates, energy consumption, and water footprint per unit of plastic produced [37].

To ensure reproducibility, heterogeneous sustainability indicators were not converted into a common 0–100 normalization framework. The underlying literature combines continuous LCA indicators with categorical evidence on biodegradation, recyclability, and resource efficiency; forcing these heterogeneous dimensions onto a single numerical scale created apparent precision that could not be reproduced independently.

Quantitative indicators are, therefore, retained in their reported units when methodological comparability is adequate, while non-continuous attributes are presented as explicit evidence categories linked to their source literature.

No min–max transformation is applied. Quantitative LCA values are interpreted within comparable functional-unit and system-boundary strata, while categorical attributes are retained as categorical evidence. This preserves the information contained in the literature without implying a universal cross-material score:

(2) $$S_{i,k}=100\cdot \frac{x_{k}^{max}-x_{i,k}}{x_{k}^{max}-x_{k}^{min}}$$

Accordingly, the analysis does not calculate an overall normalized sustainability score, use dataset minima, or maxima to generate a material ranking, or arithmetically aggregate ordinal and continuous indicators. For non-continuous attributes, the coding rule is descriptive: industrial compostability, broader validated biodegradation, established recycling-stream compatibility, limited or dedicated recycling, and feedstock/resource characteristics are reported in the form supported by the cited studies.

**Figure 11:**
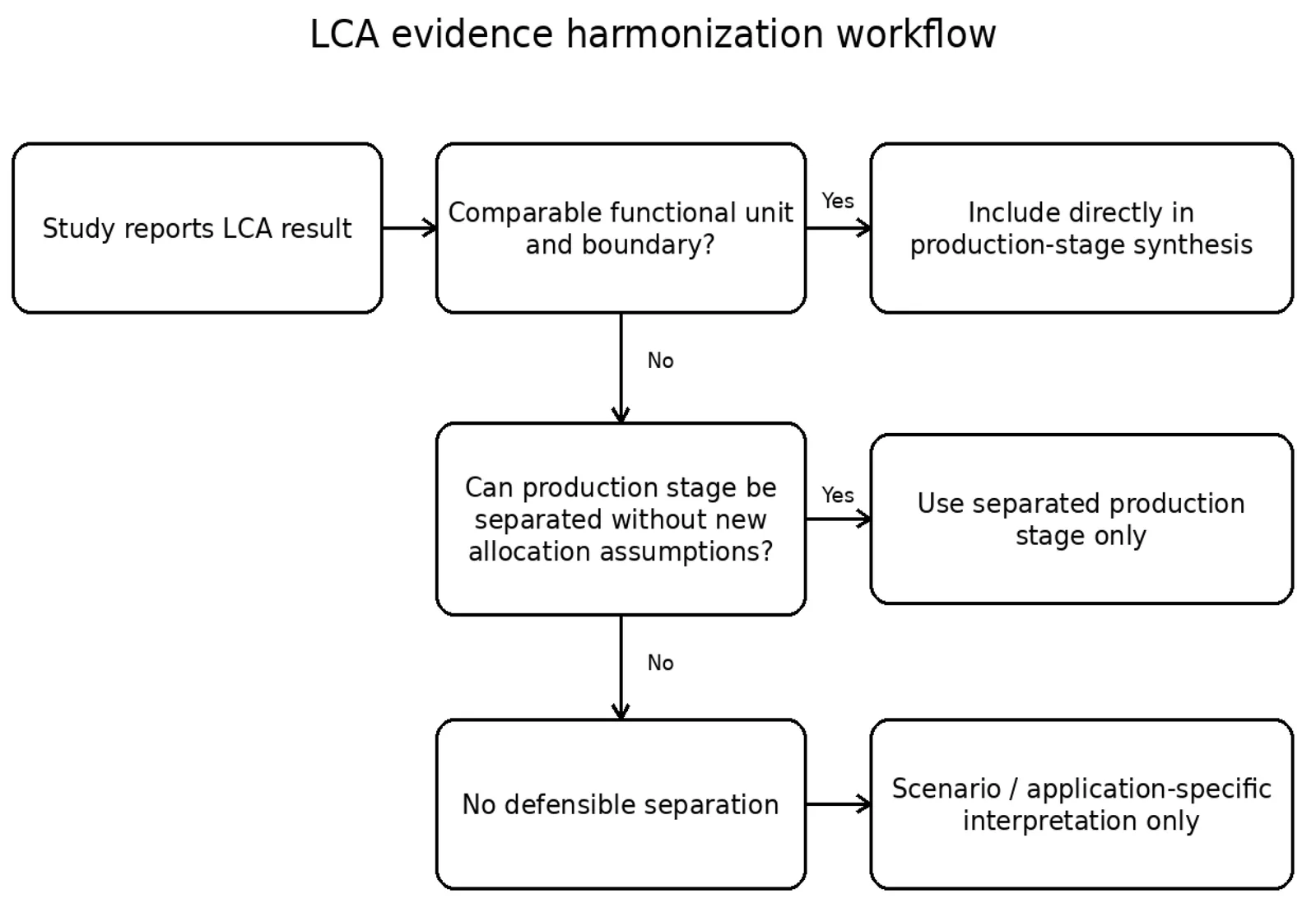
Evidence-handling workflow used to determine whether a study contributes to the production-stage quantitative synthesis or remains in scenario/categorical interpretation.

The analytical workflow does not apply a higher-is-better normalization equation. Figure 11 documents the evidence-handling decision process used to distinguish quantitatively comparable LCA evidence from scenario-specific or categorical evidence.

(3) $$S_{i,k}=100\cdot \frac{x_{i,k}-x_{k}^{min}}{x_{k}^{max}-x_{k}^{min}}$$

where multiple directly comparable quantitative values were available, medians were used only as descriptive central estimates within a defined methodological stratum; the study-level values and source references are listed in the reproducibility appendix.

This restricted use of medians reduces sensitivity to extreme scenarios without converting heterogeneous evidence into an overall sustainability score.

No overall numerical sustainability score or universal material ranking is reported.

Figure 11 summarizes the reproducibility workflow applied to LCA evidence. Studies contribute to production-stage quantitative synthesis only when their functional unit and system boundary are directly comparable or can be separated without introducing a new allocation assumption. Otherwise, they are retained for application-specific or scenario interpretation rather than converted into a synthetic score or pooled range.

The evidence-handling and harmonization rules underlying this workflow are further specified in Supplementary Table S3, which distinguishes quantitatively comparable LCA studies from evidence retained exclusively for categorical or scenario-based interpretation.

Table 4 summarizes environmental-performance evidence without converting heterogeneous attributes into a common numerical scale. Quantitative carbon-footprint evidence is reported in original units when a comparable production-stage basis is available; biodegradability, recyclability, and resource efficiency are described categorically and linked to relevant studies in the literature.

Recyclability is interpreted as a system property that depends on collection, sorting, contamination control, reprocessing yield, and market demand for recovered material; no numerical recyclability score is assigned within this framework.

This source-linked representation deliberately separates measured quantitative indicators from categorical evidence. Carbon-footprint values are retained in kg CO_2_e/kg polymer; biodegradation and recyclability statements specify the relevant environmental or infrastructure condition. This prevents cross-criterion arithmetic from being mistaken for an independently validated sustainability index [8,16,17,24].

From an application-specific perspective, biodegradable polymers may be more relevant for short-life products exposed to high leakage risk or organic-waste co-collection systems, provided that appropriate composting infrastructure exists. By contrast, for durable goods, automotive components, and closed-loop packaging systems, material longevity, mechanical reliability, and recyclability within established recovery streams are generally more decisive sustainability criteria than biodegradability alone.

#### 3.1.6. Reproducibility and Interpretation of the Comparative Evidence

Robustness is evaluated directly by comparing reported quantitative ranges within methodological strata and by assessing whether qualitative conclusions persist across the explicit end-of-life and energy-system scenarios described in Section 3.4.4.

This approach improves interpretability by avoiding an aggregate ordering that may change solely because of arbitrary scaling or weighting assumptions. The manuscript, therefore, reports trade-offs at the criterion level and reserves comparative conclusions for cases supported by source-linked LCA, engineering, infrastructure, or techno-economic evidence.

### 3.2. Material Innovations

Research on sustainable plastics has been one of the main causes of breakthroughs in the area of biopolymers, new synthesis methods, and hybrid composite materials. Such developments have the goal of minimizing the environmental footprint, enhancing mechanical features, and finding suitable substitutes for petroleum-based plastics. Below, we consider the most pertinent biopolymers, synthetic approaches, and hybrid materials that are changing the sustainable plastics world [51,52].

#### 3.2.1. Biopolymers and Natural-Based Plastics

**Figure 12:**
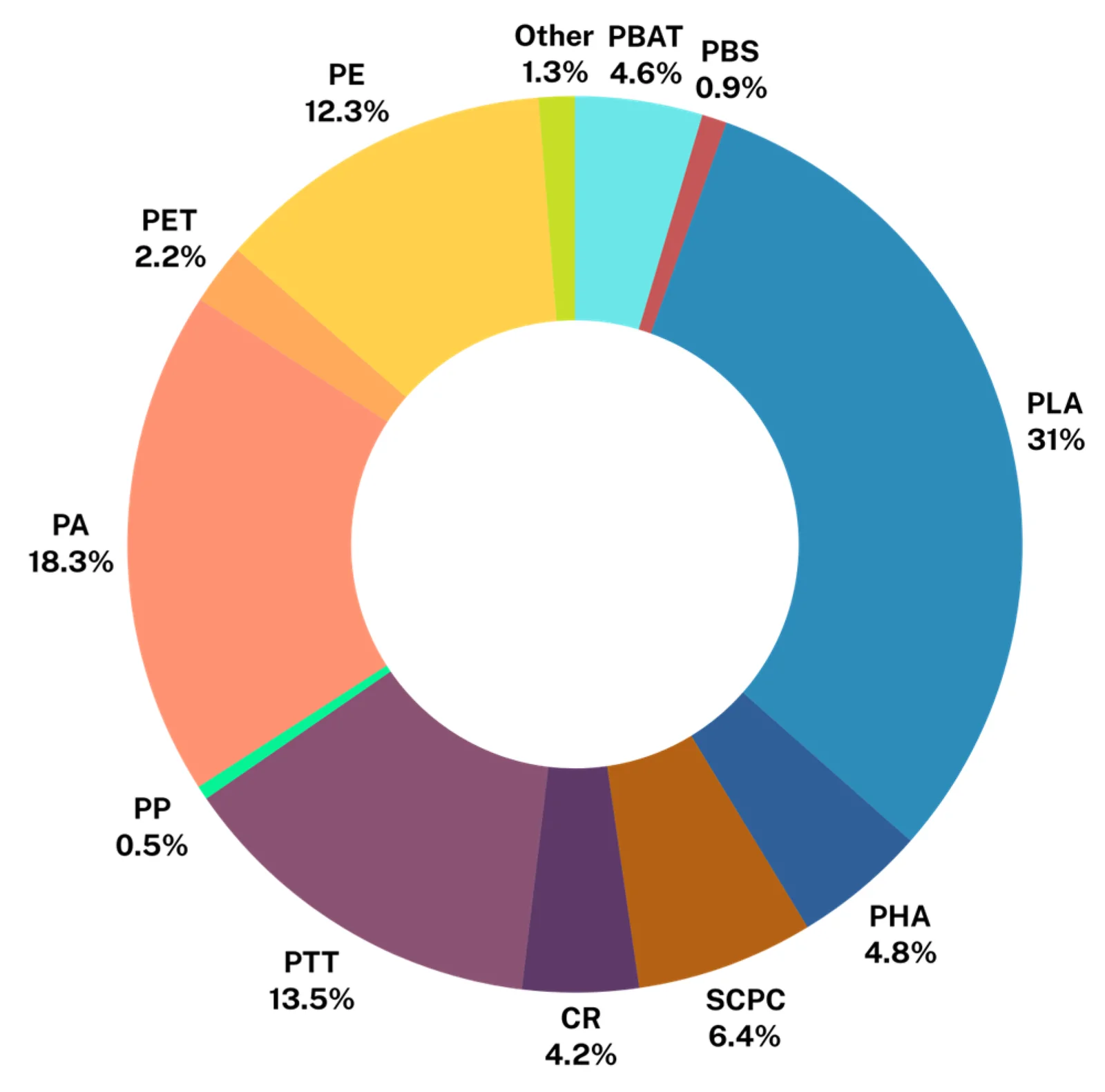
Relative distribution of major biopolymer types. The figure was created by the authors based on data and market trends reported in the bioplastics literature [16,17].

Bio-polymers, which are obtained from renewable sources, provide a solution to the problem of fossil plastic dependence. Figure 12 is a graphical representation of the relative market share of various biopolymer species, which depicts the leading position of PLA (31%) in sustainable plastic applications, followed by PA (18.3%) and PTT (13.5%). The distribution shown in Figure 12 illustrates the uneven industrial maturity of different biopolymer families, with PLA occupying a substantially larger share than emerging materials such as PBS and PEF [16,17].

Recent research has increasingly focused on improving processability, mechanical performance, barrier behavior, and controlled end-of-life characteristics [10,31,37,42].

Polylactic Acid (PLA): PLA is commonly produced from fermentable sugars and is widely used in packaging, biomedical devices, and additive manufacturing. Its principal limitations include brittleness, limited heat resistance, and sensitivity to hydrolytic degradation during processing. Blending, plasticization, nucleation, and reinforcement are used to tune flexibility, crystallinity, and thermal performance [6,51]. PLA blends are also extensively investigated for biomedical applications, where blending is used to tune degradation, drug-release behavior, and mechanical performance [53].

Polyhydroxyalkanoates (PHA): PHAs are produced through microbial biosynthesis and can biodegrade under several biologically active environments, but degradation rates and completeness depend on polymer composition, geometry, temperature, and microbial conditions. Their principal scale-up constraints remain fermentation productivity, downstream recovery, feedstock cost, and product consistency; waste-derived feedstocks and process intensification are active research directions [30,40,41].

Polybutylene Succinate (PBS): PBS is a biodegradable aliphatic polyester with useful processability, flexibility, and thermal characteristics. Its performance can be modified through blending, copolymerization, fillers, and reinforcement, while its environmental profile depends on whether succinic acid and other precursors are bio-based and on the available end-of-life pathway [25,31].

Polyethylene Furanoate (PEF): PEF is a bio-based polyester produced from furandicarboxylic acid (FDCA) and has attracted attention because of its gas-barrier and mechanical properties. These functional advantages may enable material reduction or shelf-life benefits in selected packaging applications, but such benefits must be demonstrated at the product level and balanced against the energy and capital intensity of scale-up [42,43].

Chitosan-Based Polymers: Chitosan, a deacetylated derivative of chitin, is attractive for films, coatings, adsorption media, and biomedical materials because of its film-forming ability, functional groups, and reported antimicrobial activity. Reinforcement with nanocellulose or blending with other polymers can improve strength and moisture resistance [42,45,46,54]. More broadly, polymer-based biomaterials span structural, drug-delivery, wound-care, and related biomedical applications [55]. Recent biopolymer-based wound-healing formulations further illustrate the application-specific importance of polymer chemistry, degradation behavior, and biological compatibility [56].

Beyond packaging and biomedical uses, chitosan, cellulose, starch, and related biopolymer derivatives have been investigated as low-cost adsorbents for heavy metals and nitrogen-containing pollutants in water treatment [44,47,57,58]. Agricultural residues can, likewise, be valorized into functional carbonaceous materials; for example, hay has been converted by microwave activation into bio-activated carbons for adsorption applications [59]. Adjacent biomaterials research also illustrates the value of quantitative delivery modeling: physiologically based pharmacokinetic modeling has been used to optimize a site-specific light-activatable paclitaxel prodrug system [60]. These examples are retained as application-specific evidence of polymer/biomass valorization and engineering modeling, not as direct evidence for cross-polymer LCA superiority.

Starch-Based Bioplastics: Thermoplastic starch (TPS) is used alone or in blends with polymers, such as PLA or PCL, for films, bags, and agricultural applications. Because starch is hydrophilic, plasticization, blending, and cross-linking strategies are commonly used to improve moisture resistance and dimensional stability.

Cellulose-Based Bioplastics: Cellulose derivatives and nanocellulose-containing composites are used to improve stiffness, barrier behavior, and renewable content in films and coatings. Their actual biodegradation and recycling behavior depends on chemical substitution, additives, composite architecture, and treatment conditions [28].

#### 3.2.2. Novel Synthetic Approaches

To reduce fossil dependence and process burdens, research has increasingly examined alternative feedstocks and polymerization routes, including CO_2_ utilization, lignin valorization, bio-based monomers, non-isocyanate polyurethane chemistry, dynamic covalent networks, and solvent-reduced processing [49,50,61,62,63,64,65].

- CO_2_-Based Polymers: The use of carbon capture technology is now extended to the process of transforming carbon dioxide into the polymer precursors. Covestro is the company that makes polyols from CO_2_ for the production of polyurethane foams, whose main purpose is to reduce the use of fossil fuels. Novomer’s carbon dioxide-polypropylene carbonate (PPC) is being utilized for coatings and packaging besides other uses [9,48,66].
- Lignin-Based Polymers: Lignin, which is the waste from the paper industry, is getting a new life as it is being used to make bio-based polyurethanes and thermoplastics. Lignin-reinforced polyurethane foams not only give off less fire hazard as they provide flame resistance but also become better biodeteriorable thanks to the mixture of these substances with lignin, hence constituting the most renewable sources for energy-saving purposes.
- Bio-Based Polyurethanes (Bio-PU): Since traditional PU is made from toxic isocyanates, new non-isocyanate polyurethanes (NIPU) produced from vegetable-derived polyols are a better alternative. BASF is working on bio-PU foam for environmentally friendly building materials as well as for the automotive interiors [34,51]. In construction, polymer systems, such as polyurea, are valued mainly for durability and protective performance [67]; durability in aggressive environments is, likewise, the decisive criterion when alternative binders, such as geopolymer concrete, are compared with Portland cement [68]; in both cases, service life rather than material origin governs the environmental outcome.
- Self-Healing Bioplastics: Just as nature uses self-repair to maintain life, vitrimers are the dynamic covalent parts that allow for continuous reshaping and recycling of the materials, thus making them very suitable for aerospace and flexible electronic applications [69].

#### 3.2.3. Hybrid and Composite Materials

Hybrid materials improve the mechanical, thermal, and environmental properties of bioplastics, enabling their use in high-performance sectors.

Waste-derived polymer composites also demonstrate that sustainability-oriented design can target functions beyond conventional structural reinforcement. Lyu et al. converted waste leather-derived carbon/collagen-fiber architectures into multifunctional electromagnetic-interference shielding materials, including a low-reflection nanofiber film with solar-harvesting and self-cleaning functions [70] and an ultralight collagen-fiber/PVA/Ag-nanowire aerogel with high shielding effectiveness [71]. These studies illustrate functional upcycling of waste-derived polymer/composite feedstocks; they are not used as evidence for LCA or techno-economic superiority.

- Natural Fiber-Reinforced Bioplastics: Fibers from plants such as flax, hemp, and kenaf are blended with PLA and PHA composites, which raises tensile strength and at the same time reduces weight. Automakers, such as Ford and BMW, use natural fiber-reinforced plastics for making interior panels that lead to lower emissions of the vehicle [72]. Bio-fiber reinforcement of PLA has been reviewed specifically with respect to mechanical performance, interfacial effects, and processing constraints [73].
- Nano-Enhanced Bioplastics: These incorporate nanocellulose and graphene oxide into PLA and PHA to be able to increase strength, moisture resistance, and conductivity. Nanocellulose-enhanced PLA films are oxygen barriers with a very high level which makes them perfect for food packaging [16,40,52].
- Protein-Based Bioplastics: Proteins, like casein and soy protein isolate, have been used in biodegradable films, with very high gas barrier properties. Casein-based plastics, which are mixed with glycerol, will be used for edible packaging and agricultural mulch films [5,74,75]. Protein-based sustainable-plastics alternatives have also been reviewed as a distinct design route, with performance controlled by protein source, plasticization, moisture sensitivity, and processing [76].
- Bio-Silica Composites: Rice husk silica is a filler used as a reinforcement in bioplastics, thus making the plastics more heat-resistant and also more mechanically durable. These composites are appropriate for biopolymer-based electronic casings and automotive parts [12].
- Recyclable Thermosetting Bioplastics: While traditional thermosetting plastics are almost impossible to recycle, bio-based alternatives with dynamic covalent bonds offer reshaping and reprocessing possibilities. For sustainable coatings and adhesives, researchers have created new bio-epoxy resins derived from plant oils [16,40,77,78].

### 3.3. Manufacturing Processes

#### 3.3.1. Conventional Manufacturing Processes

The transition toward sustainable polymer systems requires adaptation of manufacturing technologies to accommodate the distinct thermal, rheological, and degradation characteristics of bio-based and biodegradable materials. While conventional processing methods remain dominant in industrial practice, their optimization is essential to ensure compatibility with sustainable polymers.

Conventional techniques, such as extrusion, injection molding, blow molding, compression molding, and thermoforming, continue to be widely applied in polymer production. However, many biopolymers—including PLA, PHA, and PBS—exhibit narrower thermal processing windows and increased sensitivity to hydrolysis compared to conventional petrochemical polymers. Elevated temperatures or prolonged residence times may induce molecular-weight reduction and degradation. Therefore, energy-efficient twin-screw extrusion, optimized shear control, and precise moisture management are increasingly applied to preserve polymer integrity and mechanical performance [61,79].

Injection molding of bio-based polymers requires control of melt temperature, residence time, moisture content, injection pressure, and cooling time because hydrolysis and thermal degradation can narrow the processing window. For engineering comparison, specific energy consumption, reject rate, cycle time, and property retention should be reported together with the material formulation [37,61,66].

Blow molding processes are being adapted for bio-based alternatives, such as PEF, which offer enhanced barrier properties relative to PET but require modified cooling profiles and processing conditions to maintain structural stability [43,80,81].

Compression molding is increasingly applied to bio-based composites reinforced with natural fibers. Techniques, such as supercritical CO_2_-assisted molding, have been proposed to reduce solvent usage and improve interfacial bonding, while lowering environmental impacts [62]. Secondary operations are also important for fiber-reinforced thermoplastic composites: machining can introduce delamination, fiber pull-out and thermal damage; thus, near net-shape processing reduces both scrap and energy demand [82].

Thermoforming of biodegradable polymers requires precise temperature control to mitigate brittleness and deformation. Emerging microwave-assisted thermoforming techniques have demonstrated potential for improved heating efficiency and reduced energy consumption [32,41].

In parallel with adaptation of conventional processing routes, novel sustainable manufacturing technologies are emerging. Reactive extrusion enables in-situ polymerization without solvent use, reducing emissions and process waste [83]. Enzymatic polymerization offers a lower-temperature alternative to conventional catalysis, particularly relevant for polyester synthesis [39,57]. CO_2_-based polymerization integrates captured carbon dioxide into polymer precursors, partially substituting fossil-based feedstocks [25,32,48,66].

Additive manufacturing (3D printing) facilitates material-efficient production of customized components using PLA, PHA, and starch-based polymers, particularly in biomedical and prototyping applications. Multi-material photopolymer jetting extends this capability: blends of stiff and compliant photopolymer resins have been tuned to reproduce the mechanical behavior of cortical and cancellous bone in anatomical models used for the biomechanical testing of fracture-fixation methods [84,85]. Electrospinning technologies enable the fabrication of biodegradable nanofiber membranes suitable for filtration and packaging applications [32,41].

Overall, manufacturing adaptation and technological innovation are critical to ensuring that environmental benefits achieved at the material level are not offset by energy-intensive or inefficient production processes.

**Figure 13:**
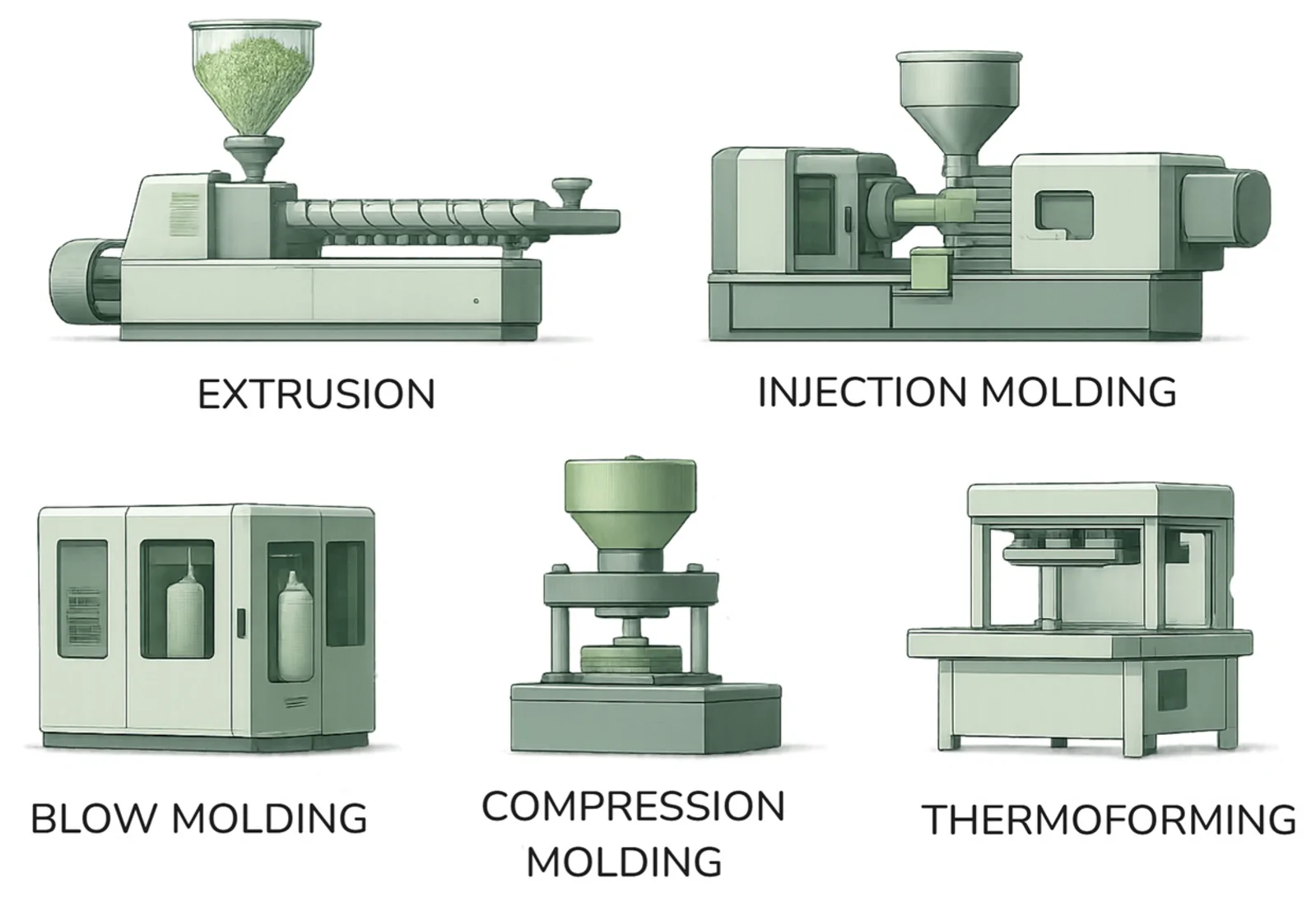
Conventional manufacturing methods in the plastics industry. This figure is an original illustration created by the authors for this study.

Despite the emergence of new routes, extrusion, injection molding, blow molding, compression molding, and thermoforming remain central to industrial polymer processing. Figure 13 summarizes these conventional technologies; their sustainability performance depends on measurable process variables such as energy per unit output, yield, scrap, cycle time, and property retention rather than on the process name alone.

#### 3.3.2. The Rise of Green Manufacturing Technologies

**Figure 14:**
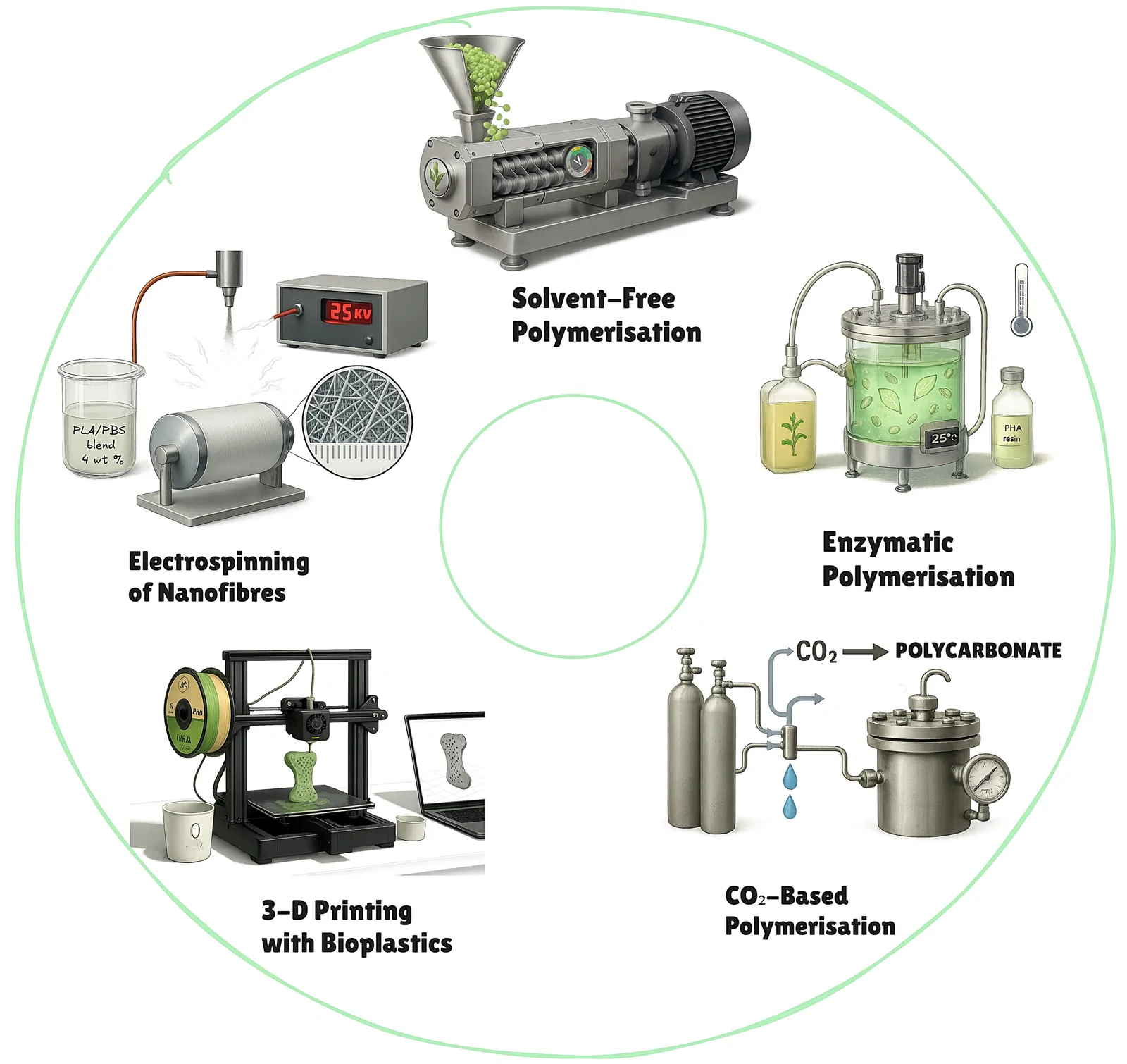
Green manufacturing technologies for sustainable polymers. This figure is an original illustration created by the authors for this study.

Beyond adapting conventional melt-processing routes, sustainable manufacturing research includes reactive extrusion, biocatalytic synthesis, CO_2_-utilization routes, additive manufacturing, and electrospinning. Their environmental value must be assessed from measurable engineering variables rather than from process labels alone. Figure 14 summarizes the principal routes, while Table 5 identifies the engineering parameters required for comparative assessment.

Reactive extrusion can combine mixing, reaction, and shaping in a continuous process and can reduce or eliminate solvent use for selected chemistries. Its environmental advantage is not automatic: conversion, residence time, specific mechanical energy, devolatilization, catalyst requirements, and off-specification material must be considered when comparing it with batch synthesis [61,66].

Enzymatic and other biocatalytic polymerization routes can operate at lower temperatures than many conventional syntheses, but lower reaction temperature does not necessarily imply lower life cycle impact. Reaction time, enzyme production and loading, substrate conversion, purification, solvent/water use, and product recovery must be included in the comparison [41,66].

CO_2_-based polymerization can substitute a fraction of fossil-derived carbon in selected polycarbonates or polyurethane intermediates. The relevant engineering variables include CO_2_ compression and purification, reaction pressure and temperature, catalyst loading, conversion, downstream separation, and the carbon intensity of process energy; captured CO_2_ content alone is, therefore, insufficient to establish a climate benefit [43,66].

Additive manufacturing can reduce tooling and enable geometry-specific material use; however, sustainability depends on part-level energy demand, print time, support or failed-print waste, feedstock preparation, anisotropic mechanical performance, and production volume. For PLA and related feedstocks, functional performance per printed part should, therefore, be evaluated together with electricity consumption rather than inferred from low material waste alone [9,77,80].

Electrospinning enables high surface-area biodegradable or bio-based fiber structures; however, process sustainability is sensitive to solvent selection and recovery, solution concentration, flow rate, applied voltage, climate control, and low-throughput operation. Its environmental profile should, therefore, be reported using solvent mass balance, energy per functional membrane area, yield, and product-performance metrics [86,87,88].

#### 3.3.3. Textile-Reinforced Composites: Manufacturing, Performance, and Circularity

Textile-reinforced polymer composites require a broader process boundary than bulk thermoplastics because sustainability is influenced by yarn preparation, weaving/knitting/braiding, preforming, impregnation, consolidation or curing, trimming, defect rates, and the fiber–matrix interface. Fiber architecture can reduce mass or improve damage tolerance, but these benefits must be balanced against textile-forming energy, resin uptake, offcuts, cure time, and end-of-life separation. Karahan and Iftekhar [20] emphasize that the manufacturing route is, therefore, a central circularity variable for textile composites rather than a neutral intermediate step.

For engineering assessment, textile composite studies should report, at minimum, textile areal density and architecture, resin/fiber fraction, impregnation yield, consolidation temperature/pressure/time, trim and defective-part losses, mechanical-property retention, and the intended repair or end-of-life route. A heavier or more energy-intensive laminate can still be preferable on a service basis if it delivers substantially longer life or lower structural mass; conversely, high mechanical performance does not guarantee circularity when thermoset matrices, mixed fibers, or complex architectures prevent recovery.

### 3.4. Life Cycle Assessment (LCA)

To improve transparency and comparability across the heterogeneous body of the LCA literature, the reviewed studies were systematically stratified according to key methodological dimensions. These include system boundaries, functional units, allocation methods, impact assessment approaches, end-of-life modeling assumptions, and geographic context. The stratification presented in Table 6 provides a structured basis for interpreting LCA results within comparable methodological groupings, rather than as aggregated averages.

This approach enables identification of the conditions under which the reported environmental advantages of specific polymers (e.g., PLA, PHA, PBS) are maintained or diminished. For instance, differences in system boundaries (cradle-to-gate vs. cradle-to-grave), allocation rules, and energy mix assumptions can significantly influence reported greenhouse gas emissions and overall sustainability rankings.

By explicitly accounting for these methodological dimensions, the analysis reduces the risk of inconsistent comparisons and supports a more robust, context-dependent interpretation of polymer sustainability performance.

To improve methodological consistency across heterogeneous LCA studies, the reviewed literature was systematically stratified according to key methodological dimensions, including:

- system boundaries (cradle-to-gate vs. cradle-to-grave);
- functional units (mass-based vs. product-based);
- allocation methods (mass, economic, system expansion);
- impact assessment methods (e.g., ReCiPe, CML, IPCC);
- end-of-life modeling assumptions.

Rather than fully harmonizing all studies—which would introduce additional modeling uncertainty—the synthesis adopts a structured comparative approach. Results are interpreted within these methodological categories; median-based aggregation is applied only within comparable groupings.

To reflect system dependence across geographic contexts, LCA results were interpreted in relation to regional energy mixes and waste management infrastructure. For instance, bio-based polymers demonstrate stronger climate benefits in low-carbon electricity systems, whereas advantages diminish in fossil-intensive grids. Similarly, biodegradable polymers require access to industrial composting infrastructure to achieve intended environmental benefits, which is unevenly distributed across regions.

Although a fully regionalized meta-analysis was not feasible due to data heterogeneity, comparative interpretation was conducted across representative system archetypes (low-carbon vs. high-carbon energy systems; advanced vs. limited waste infrastructure).

Regional variability further reinforces system dependence: in low-carbon, infrastructure-rich regions (e.g., EU), recyclable polymers such as PET and PEF tend to outperform biodegradable alternatives due to efficient material recovery, whereas in regions with fossil-intensive energy systems or limited waste management infrastructure, biodegradable polymers may offer relative advantages in leakage-prone environments.

A sensitivity-oriented interpretation is employed, whereby reported ranges are discussed in relation to underlying assumptions (e.g., electricity mix, land-use change inclusion), allowing identification of conditions under which comparative rankings between polymers (e.g., PLA, PHA, PBS) may shift. Life cycle assessment (LCA) is the primary quantitative framework for evaluating environmental burdens across the full value chain of polymer materials. In the context of sustainable polymers, LCA enables consistent comparison between bio-based, recyclable, biodegradable, and fossil-based plastics by accounting for resource use and emissions from feedstock acquisition through manufacturing, use, and end-of-life (EoL) treatment. Importantly, LCA outcomes are highly sensitive to methodological choices, including functional unit definition, system boundaries, allocation rules, energy mix assumptions, land-use change modeling, and EoL scenario selection. Consequently, interpretation should emphasize relative trends and uncertainty ranges rather than single-point values [89].

The methodological criteria applied to harmonize LCA studies, including functional-unit equivalence, system-boundary compatibility, and the treatment of non-comparable data, are detailed in Appendix A (Table A2).

For production-stage comparison, quantitative environmental indicators were harmonized only when a defensible cradle-to-gate contribution could be identified. The target functional unit was 1 kg of polymer resin at the factory gate. Cradle-to-gate studies reporting this unit directly were retained without conversion. Cradle-to-grave studies were eligible for production-stage pooling only when the publication separately reported the production/resin stage or provided inventory/contribution data from which the pre-use, pre-end-of-life production contribution could be isolated without introducing a new allocation assumption. End-of-life credits, use-stage burdens, avoided-product credits, and disposal emissions were not algebraically subtracted from a single cradle-to-grave total when stage-resolved data were unavailable. Such studies were retained for scenario interpretation but excluded from the production-stage quantitative range. Product-based functional units were not converted to 1 kg polymer unless the study explicitly reported product mass and equivalent functional performance; otherwise, they were analyzed within their application-specific stratum. Studies with incompatible allocation rules, unreported biogenic-carbon treatment, or non-separable system boundaries were therefore not forced into the pooled mass-based comparison. Appendix A (Table A2) summarizes these inclusion/conversion rules.

The study by Koçak Mutlu et al. [19] illustrates why this distinction matters. Virgin and recycled LDPE films with ZnO modification were produced by extrusion, characterized by FTIR, SEM, TGA, DSC, and tensile testing, and evaluated by cradle-to-gate LCA using a 1 kg film functional unit. Because formulation, recycled content, processing, mechanical/thermal performance, and environmental burden were evaluated within the same product system, the study demonstrates that environmental performance cannot be separated from formulation and delivered function. The most favorable option emerged from a specific combination of recycled content and additive level rather than from polymer identity alone.

For durable engineering products, the concept can be expressed as impact per delivered functional service: I_service = (I_material + I_manufacturing + I_maintenance + I_EOL)/S, where S represents the verified service delivered (e.g., load cycles, component-years, or barrier-area-years). The review does not calculate this metric across all materials because the primary literature does not report a common service variable; however, the framework prevents a lower kg CO_2_e/kg value from being interpreted as preferable when substantially more material, replacement frequency, or shorter service life is required.

Current LCA practices for polymer systems are guided by ISO 14040 and ISO 14044 frameworks; however, significant variability persists in their application, particularly regarding system boundary selection, allocation procedures, and end-of-life modeling. Inconsistent reporting of functional units and impact categories further limits comparability across studies. Progress toward harmonization requires standardized reporting protocols, including: consistent functional units (e.g., mass-based and application-based dual reporting), transparent declaration of system boundaries and allocation methods, inclusion of sensitivity analyses reflecting key system variables such as energy mix and end-of-life pathways.

To improve comparability and reproducibility of polymer LCA studies, several practical standardization pathways are recommended:

- Dual functional unit reporting, including both mass-based (e.g., kg polymer) and application-based units (e.g., packaging system), to enable consistent cross-study and real-world comparisons;
- Standardized end-of-life scenario definitions, including reference cases for recycling, landfill, incineration, and industrial composting;
- Explicit reporting of energy mix assumptions, particularly electricity carbon intensity, given its strong influence on comparative results;
- Mandatory sensitivity analysis across key system variables (e.g., energy mix, land-use change, end-of-life pathways) to assess robustness of conclusions.

#### 3.4.1. Key Methodological Aspects for Polymer LCAs

Polymer LCAs typically evaluate impacts across four interdependent stages:

- Feedstock and raw material sourcing;
- polymer synthesis and processing;
- use phase;
- EoL management.

For fossil-based polymers, feedstock extraction and refining contribute substantially to cumulative energy demand and greenhouse gas (GHG) emissions. For bio-based polymers, upstream impacts may shift toward agricultural inputs (fertilizers, irrigation, land occupation) and potential indirect land-use change, which can offset carbon benefits, depending on regional practices and modeling choices. In addition, biogenic carbon accounting requires careful treatment to avoid double counting or misinterpretation when comparing bio-based and fossil-based systems [31,86,90].

Allocation procedures across the reviewed LCAs varied between mass-based allocation, economic allocation, and system expansion approaches, particularly in bio-refinery contexts where co-products (e.g., animal feed, bioenergy) are generated. As allocation choice significantly influences reported greenhouse gas emissions, the present synthesis retained values as reported in the original studies and emphasized median ranges to mitigate methodological dispersion.

#### 3.4.2. Comparative Evidence: Bioplastics Versus Conventional Plastics

Comparative LCA studies show that some bio-based polymers can have lower production-stage greenhouse gas burdens than fossil-based counterparts under specific assumptions; however, the magnitude, and even the direction of the difference, depend on system boundary, electricity mix, biogenic-carbon accounting, land-use treatment, and allocation [8,16,17]. Table 7 identifies representative supporting sources and the relevant boundary limitation for each polymer. The values are treated as literature synthesis envelopes rather than as constants or outputs of a new meta-model. Appendix A (Table A3) provides the study-level extraction fields required to audit each quantitative entry, including source, functional unit, boundary, geographic context, GHG value, energy value, and inclusion status.

The source-linked evidence supporting the comparative LCA interpretation presented in Table 4 and Table 7 is further documented in Supplementary Table S2, which identifies the relevant source groups and methodological boundaries associated with the reported polymer-specific environmental performance.

It should be noted that underlying inventory databases (e.g., Ecoinvent, GaBi, regional LCI datasets) differ across studies in terms of background electricity mixes, agricultural emission factors, and end-of-life modeling. Consequently, reported emission ranges reflect both technological and database-related variability.

Citation-to-claim audit: all quantitative statements in Section 3.4, Section 3.5 and Section 3.6 were rechecked against the cited source type. Review articles are used primarily for synthesis and contextual interpretation, whereas numerical ranges are accompanied by primary LCA/TEA studies or original inventory datasets where available. Post-cutoff references are treated as contextual updates and are not counted within the *n* = 193 systematic dataset.

Table 8 integrates representative techno-economic indicators with LCA results in order to assess the combined environmental and economic feasibility of selected polymer systems. While LCA results provide insight into potential environmental advantages, Table 8 highlights that these benefits are often constrained by economic and scalability factors.

Bio-based polymers, such as PLA, PHA, and PBS, may achieve lower greenhouse gas emissions under optimized conditions; however, their production costs remain significantly higher than those of conventional polymers, primarily due to feedstock requirements, process complexity, and limited economies of scale.

In contrast, fossil-based polymers, such as PET and PP, although not inherently low-carbon, benefit from mature industrial infrastructure, optimized supply chains, and well-established recycling systems, resulting in lower production costs and higher system integration.

The combined analysis demonstrates that environmental performance alone is insufficient to determine material sustainability. Instead, viable solutions must balance environmental impact with economic feasibility and infrastructure compatibility. This finding reinforces the concept that polymer sustainability is fundamentally system-dependent, requiring integrated LCA–TEA evaluation for robust decision-making.

#### 3.4.3. End-of-Life (EoL) Scenarios and Circularity Implications

End-of-life management is a dominant driver of polymer LCA outcomes. Mechanical recycling generally offers strong climate benefits by displacing virgin polymer production; however, performance depends on collection rates, contamination, downcycling, and regional energy mixes. Chemical and enzymatic recycling pathways may improve circularity for difficult-to-recycle streams; however, their net benefits depend on process yields, solvent/energy demand, and scale maturity. For compostable plastics, environmental performance is contingent on access to industrial composting infrastructure and correct waste sorting. When compostable polymers enter conventional recycling streams, they may reduce recovery efficiency through contamination; conversely, when landfilled, many biodegradable polymers degrade slowly, limiting expected benefits.

A recurring concern in the literature is that biodegradability does not automatically guarantee environmental advantage under real-world mismanagement. Degradation depends on polymer chemistry, formulation, specimen geometry, temperature, moisture, oxygen availability, and microbial activity; incomplete degradation or fragmentation can, therefore, remain an environmental concern [8,27,91]. For mixed or contaminated streams that cannot be recycled mechanically, thermochemical routes, such as microwave pyrolysis [92] and conversion into construction products such as bricks made from waste HDPE and PP [93], are being investigated, although their net benefits depend on energy input, emissions, and the displaced product.

#### 3.4.4. Scenario-Based Robustness Analysis of System-Level Assumptions

The scenario analysis operationalizes the engineering chain proposed in this review: material → formulation → manufacturing route → functional performance → service life → collection → recycling/composting or disposal → regional energy system → economic feasibility. It is a scenario-based robustness analysis, not a parametric sensitivity analysis derived from a newly constructed process model. The purpose is to test whether the direction of the synthesis changes across evidence-supported system archetypes rather than to estimate derivatives, elasticities, or a universal break-even threshold. To strengthen interpretation without implying an unreported numerical model, a qualitative scenario discussion grounded in the literature is used. No new parametric sensitivity coefficients, break-even values, or material-ranking calculations are claimed.

Three literature-grounded system archetypes are discussed:

Scenario 1: Low-carbon energy system (renewable-dominant electricity mix)

Under lower-carbon electricity conditions, production-stage LCA results for PLA and other bio-based polymers can be lower than fossil counterparts in some studies, but the magnitude of the difference varies with electricity mix, feedstock treatment, allocation, biogenic-carbon accounting, and plant assumptions [16,17,38]. PHA is particularly route-dependent because fermentation substrate, productivity, aeration, downstream recovery, and co-product treatment vary substantially [40,41]. This scenario is, therefore, interpreted directionally rather than as a recalculated material ranking;

Scenario 2: High-carbon energy system (fossil-intensive electricity mix)

Under fossil-intensive electricity and heat supply, the production-stage advantage reported for some bio-based polymers can narrow or disappear because fermentation, purification, drying, polymerization, and conversion are energy-sensitive. The reviewed LCA literature, therefore, supports a conditional conclusion rather than a universal ordering: energy-system assumptions must be reported together with the polymer result [16,17,38,42];

Scenario 3: End-of-life pathway variation (recycling vs. composting vs. landfill)

End-of-life assumptions strongly influence comparative outcomes. Mechanical recycling of PET, HDPE, or PP can reduce burdens when recovered resin displaces virgin production, but benefits depend on collection, sorting, contamination, yield, reprocessing degradation, and substitution assumptions [7,8,22,39]. Compostable polymers, such as PLA or PBS, require compatible industrial composting and correct sorting; landfill conditions may not provide the temperature, oxygen, moisture, or residence-time conditions assumed by compostability tests [28,31]. In leakage-prone systems, validated biodegradation behavior may become relevant, but leakage and microplastic consequences are incompletely represented in many conventional LCA models [4,8,27,94]. These are qualitative, literature-grounded scenario interpretations rather than newly calculated rankings.

The end-of-life interpretation was expanded into an operational scenario matrix rather than a generic statement about recycling or composting. The archetypes are defined by observable system conditions—availability and effectiveness of collection/sorting, access to industrial composting, dominance of landfill/incineration, leakage risk, and organic-waste co-collection—without imposing universal numerical cutoffs that are not consistently reported across regions. Quantitative global evidence is used only to anchor the plausibility of these contrasting archetypes: Geyer et al. [1] estimated that, by 2015, approximately 9% of plastic waste had been recycled, 12% incinerated, and 79% accumulated in landfills or the natural environment; OECD [2] similarly reported that in 2019, only 9% of plastic waste was recycled after losses and 22% was mismanaged or leaked. Jambeck et al. [94] further identified land-based mismanaged waste as a major driver of marine leakage.

**Figure 15:**
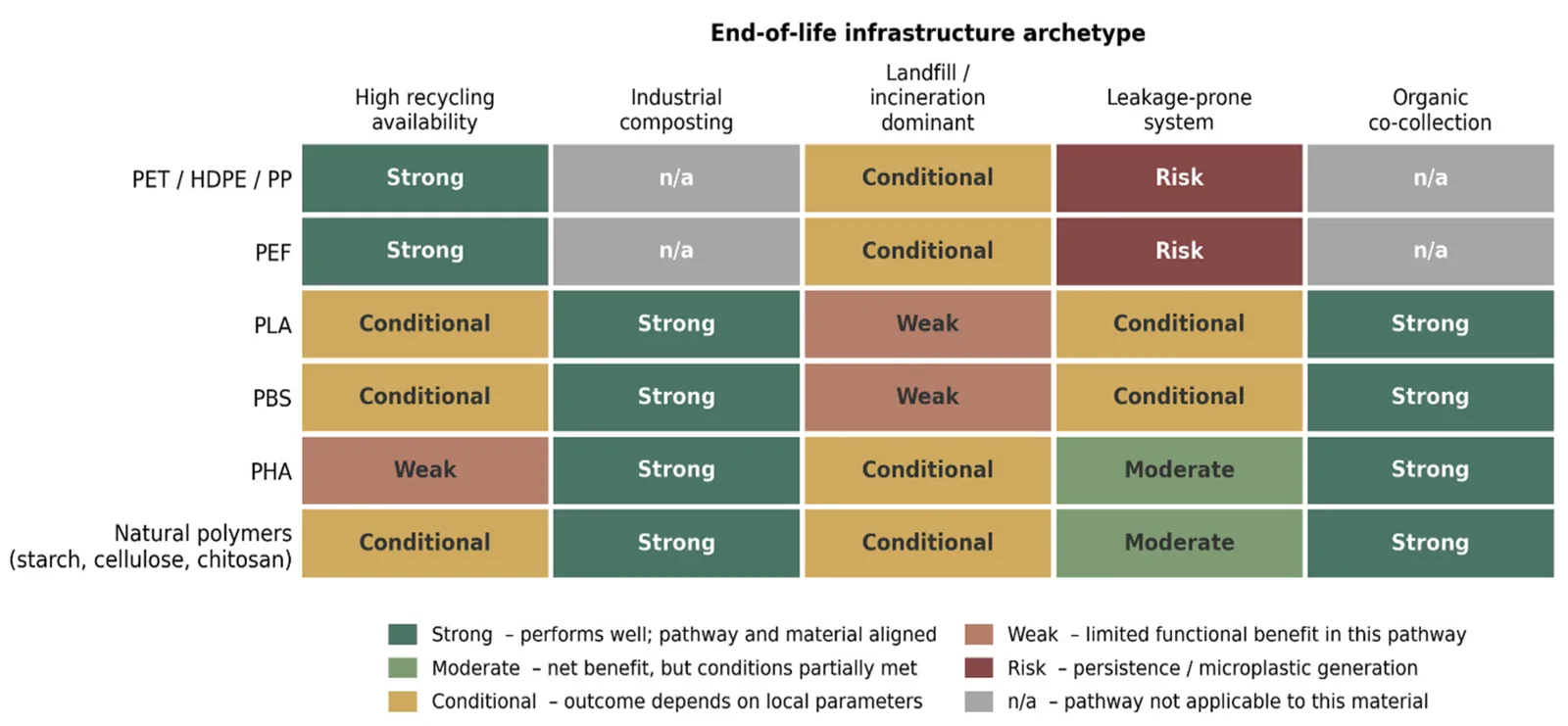
Polymer–archetype performance matrix synthesizing system-dependent end-of-life outcomes for the polymer systems reviewed in this manuscript.

The interaction between polymer chemistry and infrastructure archetype is summarized in Figure 15, which positions each polymer system on the ordinal performance scale across the five archetypes defined in Table 9.

The visualization makes the system-dependent claim explicit at the row level: PET-family polymers transition from a “Strong” classification under high-recycling conditions to “Risk” classification under leakage-prone systems, while PHA shows the opposite trajectory—weak performance where mechanical recycling dominates, but strong performance precisely in the leakage and organic-stream pathways where conventional recyclables fail. PLA and PBS occupy intermediate positions whose realized sustainability depends on the availability of certified industrial composting or organic-waste co-collection. No polymer dominates across all archetypes, which directly contradicts intrinsic-material narratives of sustainability and reinforces the conclusion that material selection must be co-specified with the end-of-life infrastructure that will receive the product.

Performance is expressed on a six-level ordinal scale (Strong, Moderate, Conditional, Weak, Risk, n/a) rather than as numerical scores, in order to avoid implying meta-analytic precision beyond what the heterogeneous LCA, biodegradation, and infrastructure literature supports. Cell assignments are evidence-informed classifications consistent with the scenario conditions in Table 9, the LCA evidence ranges reported in Section 3.4, and the recycling and biodegradation literature reviewed throughout. Reading any row horizontally illustrates the central thesis of this review: a polymer’s environmental performance is not an intrinsic material property but a function of the end-of-life infrastructure available, with the same material shifting from favorable to unfavorable classifications across infrastructure conditions.

The matrix clarifies that recyclability and biodegradability are not competing intrinsic virtues. They are pathway-dependent properties whose environmental value depends on collection, sorting, treatment capacity, and the avoided burdens credited in the LCA model. Therefore, the strongest sustainability claims are supported only when material design and end-of-life infrastructure are jointly specified.

#### 3.4.5. Economic Viability and System-Level Trade-Offs

Environmental performance must be interpreted alongside techno-economic feasibility and scale readiness. The available TEA evidence is substantially more heterogeneous than the LCA evidence, particularly for PHA, PBS, and early-scale PEF. For this reason, the revised synthesis reports source-specific economic evidence and maturity constraints rather than generic cost multipliers. This distinction prevents favorable LCA results from being interpreted as proof of near-term industrial competitiveness.

To make the economic component explicit, the techno-economic evidence was interpreted through six semi-quantitative criteria: the reported production-cost multiple relative to a conventional-polymer baseline; cost-competitiveness class; capital and scale intensity; supply-chain maturity; learning potential; and industrial maturity stage (Table 10). No de novo production-cost model is constructed in this review. Cost multipliers are retained only where they were reported or could be expressed on a directly comparable basis in the literature; heterogeneous TEA results are not pooled as a formal meta-analysis. Where a relative cost ratio is reported, its generic interpretation is R_cost = C_polymer/C_reference, with the reference polymer stated explicitly. Because feedstock basis, plant scale, energy price, allocation, and capacity utilization differ among studies, the resulting ranges are treated as contextual classes rather than predictive prices.

The source-linked techno-economic evidence supporting the cost and industrial maturity assessments presented in Table 8 and Table 10 is summarized in Supplementary Table S4, with particular emphasis on production constraints, scale-related limitations, and the comparability of economic indicators.

Reported cost multiples are presented as ranges rather than point estimates because the underlying TEA studies adopt heterogeneous system boundaries—including feedstock cost basis, plant scale, energy price assumptions, allocation methods, and capacity-utilization rates. The clustering is, therefore, semi-quantitative by design: it preserves the comparative structure required for system-level interpretation while avoiding the false precision that single-point cost values would imply. The reported ranges are consistent with the techno-economic literature synthesized in this review and are intended to support relative positioning of polymer systems rather than absolute cost forecasting.

Scalability is classified separately from technical feasibility. Laboratory feasibility indicates successful synthesis or processing at the research scale; pilot feasibility demonstrates repeatable operation on integrated pilot equipment; demonstration scale provides sustained operation under industrially relevant conditions; commercial scale requires reliable supply, quality control, throughput, and market delivery; economic competitiveness additionally requires costs and the risk to approach the incumbent alternative for the target function. A material or process is, therefore, not described as “industrially scalable” solely because laboratory production has been demonstrated.

Three broad economic regimes can be distinguished without imposing unsupported universal cost multiples. Mature commodity polymers (PET, PP, HDPE) benefit from established production and recovery infrastructure. PLA is commercially established but remains sensitive to feedstock, fermentation efficiency, energy price, and plant scale. PHA remains strongly constrained by fermentation productivity and downstream recovery, as emphasized by the pilot-scale review in [38,40,41]. For PBS, Rajendran and Han reported a study-specific MSP of 3.5 USD/kg for a food-waste-based process [17,38]. PEF cost evidence remains dominated by modeled and early commercial assumptions [16,38,42]. Accordingly, Table 8 and Table 10 report source-specific cost evidence rather than generic cross-polymer multipliers.

The combined LCA–TEA interpretation shows that environmental advantage and implementation readiness are separate questions. A favorable production-stage carbon footprint may coexist with high capital intensity, uncertain feedstock supply, limited scale, or incompatible recovery infrastructure; conversely, an incumbent polymer may retain strong system performance because collection, sorting, and reprocessing are already established.

### 3.5. Scalability and Adoption

A successful move from traditional plastics to sustainable alternatives is dependent upon governmental policies, industrial readiness, and consumer demand. Biodegradable plastics together with recyclable polymers are environmentally friendly, however, the big challenges to such large-scale adoption remain high prices, technical difficulties, and supply-chain adaptation. The automotive industry, a major consumer of plastics, is also incorporating bio-based materials, light composites, and recyclable alternatives to accomplish sustainability goals [11].

Governments are expanding plastics-related regulation through instruments such as extended producer responsibility, recycled-content requirements, restrictions on selected single-use products, and waste-management targets. However, policy design and implementation differ substantially among jurisdictions, creating uncertainty for multinational supply chains and recovery systems [2,7,8].

The large-scale deployment of sustainable polymer systems depends on regulatory alignment, industrial readiness, economic feasibility, and societal acceptance. Despite the environmental advantages demonstrated under specific conditions, widespread adoption remains constrained by cost differentials, infrastructure limitations, and supply-chain uncertainties [11].

**Figure 16:**
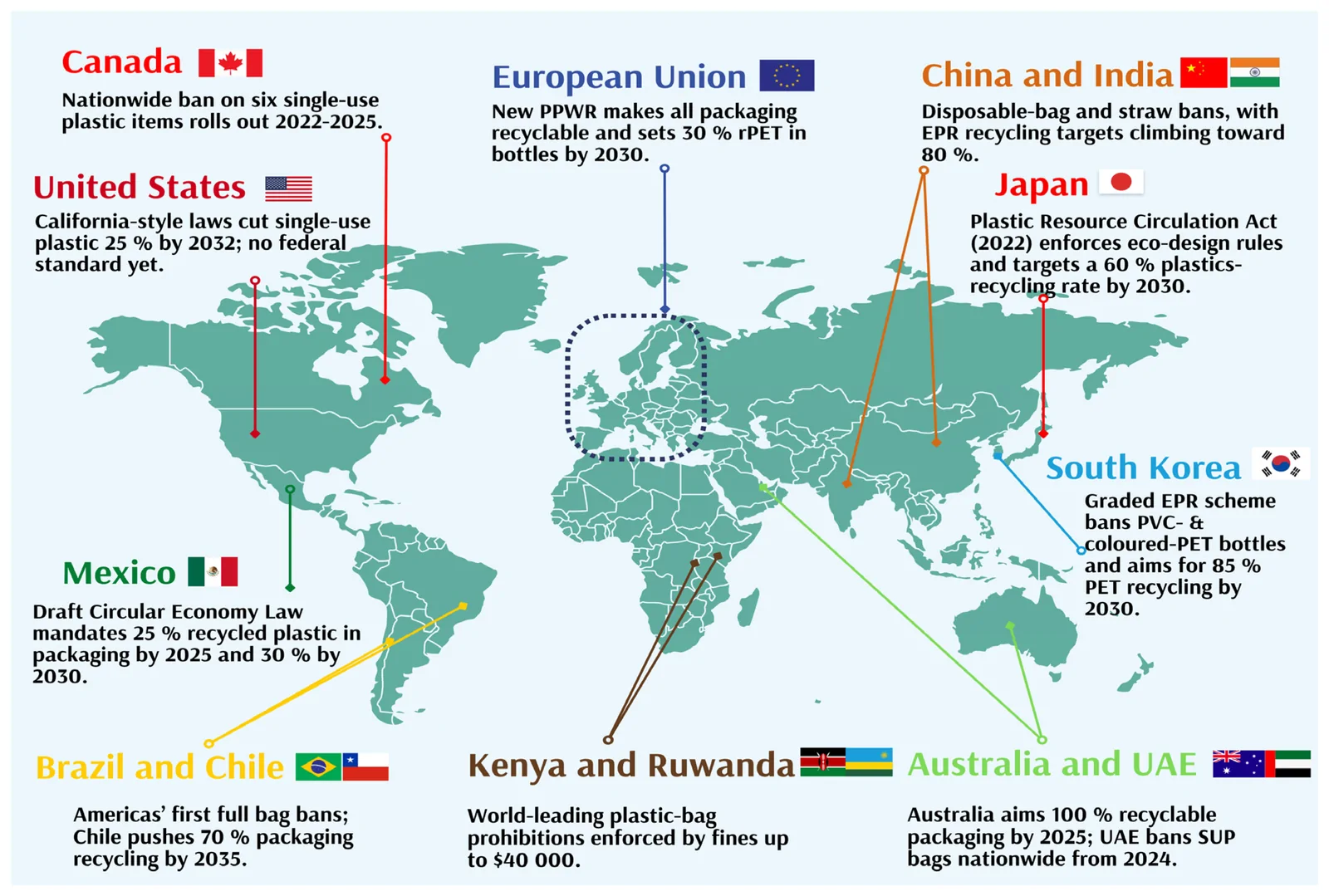
Global regulatory hotspots for sustainable plastics: headline measures in major jurisdictions. This figure is an original illustration created by the authors for this study.

Although a formal techno-economic meta-analysis was beyond the scope of this review, source-specific cost and scale evidence was used to contextualize LCA results. Because plant capacity, feedstock price, energy price, currency year, downstream recovery, and allocation assumptions differ substantially, no single generic cost premium is reported for bio-based polymers. Instead, study-specific values are cited directly, where available, and otherwise interpreted qualitatively, Figure 16.

#### 3.5.1. Regulatory Frameworks

Regulatory measures play a significant role in shaping demand for sustainable polymers. The European Green Deal and the Single-Use Plastics Directive promote recyclable and bio-based materials by setting ambitious circular economy targets. The End-of-Life Vehicle (ELV) Directive requires high recyclability rates in automotive components, thereby stimulating interest in bio-composites and recyclable thermoplastics.

Regulatory fragmentation across regions can create uncertainty for producers operating in multiple markets because definitions, labeling requirements, recycling targets, compostability rules, and producer-responsibility obligations are not fully harmonized [2,7,8]. The engineering implication is that material design should be evaluated against the specific regulatory and waste-management context in which the product will be marketed.

#### 3.5.2. Industrial Integration

Scaling sustainable polymers requires alignment between material properties, manufacturing systems, supply chains, and recovery infrastructure. PHA commercialization remains limited by fermentation productivity and downstream recovery costs [38,40,41], while early PEF deployment is influenced by capital intensity and supply-chain maturation [16,38,42]. The manuscript, therefore, avoids unsupported universal cost multiples.

Manufacturing infrastructure is often optimized for fossil-based polymers, requiring process adjustments for bio-based materials. Adaptation of extrusion temperatures, moisture control, and molding parameters is necessary to prevent degradation and maintain mechanical performance.

Recycling infrastructure presents additional challenges. Existing systems are primarily designed for PET, HDPE, and PP streams. The introduction of biodegradable polymers into conventional recycling pathways may reduce material purity if not properly segregated [41].

Supply-chain stability also influences scalability. Agricultural feedstocks are subject to seasonal variability, land-use competition, and price fluctuations. Waste-derived or CO_2_-based feedstocks may reduce such dependency but remain limited in commercial deployment [1,94].

#### 3.5.3. Market and Societal Perspectives

Consumer behavior affects realized end-of-life performance because incorrect sorting can contaminate recycling or organic-waste streams. Clear labeling and collection instructions are, therefore, important complements to material design, especially for products marketed as recyclable, compostable, or biodegradable [7,8].

Retail and automotive sectors are increasingly incorporating bio-based materials in packaging and interior components to meet sustainability targets. Nevertheless, large-scale implementation depends on cost competitiveness, performance reliability, and regulatory certainty. Therefore, successful scaling requires coordinated policy frameworks, infrastructure development, cost reduction through technological maturation, and improved consumer education.

### 3.6. Challenges and Limitations

Despite substantial progress in sustainable polymer development and recycling technologies, the large-scale transition from fossil-based plastics to bio-based and biodegradable alternatives remains constrained by technical, economic, and systemic barriers. While sustainable polymers offer potential reductions in fossil resource dependency and greenhouse gas emissions, their performance limitations, production costs, and environmental trade-offs continue to restrict widespread industrial adoption.

While chemical recycling is often presented as a solution for complex plastic waste streams, its high energy demand and process intensity must be critically considered. In several cases, the energy required for depolymerization and purification may offset environmental benefits, particularly when powered by fossil-based energy sources. This highlights the risk of greenwashing, where technological potential is emphasized without full life cycle validation.

#### 3.6.1. Technical Challenges: Performance Trade-Offs and Processing Constraints

One of the primary technical challenges associated with sustainable polymers is achieving a balance between biodegradability and high mechanical performance. Conventional polymers, such as PET, HDPE, and PP, are widely used due to their tensile strength, thermal stability, and chemical resistance. In contrast, many biodegradable polymers—including PLA, PHA, and starch-based materials—exhibit reduced impact resistance, lower heat deflection temperatures, and increased susceptibility to hydrolytic and UV-induced degradation [96].

These performance trade-offs become particularly critical in high-demand applications, such as automotive components, where materials must withstand elevated temperatures, mechanical stress, and long service lifetimes. Bio-based polyamides (e.g., nylon-11 and nylon-12) and flax-reinforced PLA composites have been explored in interior automotive applications, including door panels and dashboard elements; however, improvements in thermal resistance and impact strength remain necessary to match conventional polymer performance standards [27,58,80]. Structured ecodesign methods can help to resolve these contradictions at the design stage; for example, TRIZ-based analysis has been used to select combinations of synthetic and natural fibers that improve the performance of polymeric resins while reducing their environmental burden [97]. Material efficiency can also be increased by placing reinforcement only where it is needed: in polyurethane composites, dovetail-type interlocking interfaces between reinforced and unreinforced regions increased the load-bearing capacity per unit fiber volume by 33.2% relative to uniformly reinforced parts, while reducing fiber consumption by about 19% [21].

Processing constraints further complicate implementation. Many biopolymers exhibit narrow thermal processing windows, leading to degradation prior to reaching optimal molding temperatures. For instance, PLA and PBS require precise control of temperature and moisture during extrusion and injection molding to minimize defects and material waste. Advanced solutions, such as low-energy twin-screw extrusion and AI-assisted process monitoring, have been proposed to improve process stability and reduce degradation; nevertheless, large-scale industrial adoption of these technologies remains limited [25,62,98].

Additionally, compatibility with existing production infrastructure poses challenges. Bioplastics often require modified molding temperatures, drying protocols, and tooling configurations compared to conventional petrochemical polymers. Retrofitting high-volume manufacturing lines—particularly in packaging and automotive sectors—can involve significant capital expenditure, thereby slowing industrial transition [15]. The same applies to joining and assembly: for ultrasonically welded assemblies, such as glass-fiber-reinforced PBT housings with e-PTFE membranes, weld quality depends closely on the matrix, reinforcement content, and joint geometry; thus, any material substitution requires requalification of the downstream joining process [99].

#### 3.6.2. Economic Barriers: Cost Structure and Feedstock Dependence

Economic competitiveness remains a major barrier to sustainable polymer adoption; however, the magnitude of the premium is polymer- and route-specific. In the revised synthesis, cost evidence is reported only when the underlying source provides a traceable value or a clearly defined commercialization constraint. For example, a food waste-based PBS process reported an MSP of 3.5 USD/kg [17,38], while the pilot-scale PHA literature emphasizes feedstock, fermentation productivity, downstream recovery, and scale as dominant cost drivers [38,40,41].

Conventional polymers benefit from highly optimized global supply chains and mature petrochemical infrastructure. In contrast, bio-based polymer production frequently depends on agricultural feedstocks, such as corn, sugarcane, or cassava, or on microbial fermentation processes that require significant resource input. Variability in biomass availability, land-use pressures, and energy intensity further influences production costs and scalability [23,38].

Without sustained policy incentives, technological improvements, and an expansion of industrial capacity, cost disparities may continue to limit the substitution of conventional plastics in price-sensitive markets.

#### 3.6.3. Systemic and Environmental Trade-Offs

Beyond technical and economic constraints, systemic challenges also affect sustainable polymer deployment. Infrastructure for composting and advanced recycling remains unevenly distributed globally, and the improper sorting of biodegradable plastics can compromise existing recycling streams.

Furthermore, while sustainable polymers may reduce greenhouse gas emissions under optimized conditions, environmental trade-offs may arise in other impact categories, including land use, water consumption, and agricultural inputs. Consequently, sustainability assessments must consider full life cycle implications rather than isolated performance metrics.

The challenges associated with sustainable polymers are multidimensional and interrelated. Performance limitations, processing constraints, infrastructure gaps, and economic barriers collectively influence adoption trajectories. Addressing these limitations requires coordinated advancement in materials engineering, process optimization, infrastructure development, and policy alignment to ensure that environmental benefits demonstrated at the laboratory scale translate into credible, large-scale sustainability outcomes.

### 3.7. Toward Harmonized Sustainability Assessment Frameworks

Despite the widespread use of ISO 14040 and ISO 14044 standards, significant variability persists in the application and reporting of LCA studies for polymer systems. This lack of harmonization limits comparability across studies and reduces the reliability of cross-material assessments.

To address these challenges, several practical standardization pathways are proposed:

- Dual functional unit reporting:

Studies should report results both on a mass basis (e.g., kg polymer) and an application basis (e.g., packaging unit), enabling both material-level and system-level comparisons;

- Transparent system boundary definition:

All studies should explicitly state whether results are cradle-to-gate or cradle-to-grave, with clear separation of production and end-of-life contributions;

- Standardized end-of-life scenarios:

A minimum set of reference scenarios (e.g., recycling, landfill, incineration, industrial composting) should be consistently applied to enable cross-study comparison;

- Explicit allocation method reporting:

Allocation choices (mass, economic, system expansion) should be clearly stated and, where possible, sensitivity-tested;

- Mandatory sensitivity analysis:

Key variables, such as electricity mix, land-use assumptions, and end-of-life pathways, should be systematically varied to assess robustness of conclusions;

- Multi-indicator reporting:

In addition to climate impact, studies should include indicators, such as energy demand, water use, and eutrophication, to avoid single-metric bias. Implementation of these recommendations would significantly improve the reproducibility, comparability, and decision-making relevance of LCA studies in sustainable polymer research. Harmonized methodologies are essential to support evidence-based policy development and to enable the reliable identification of system-compatible materials.

Therefore, chemical recycling should not be interpreted as an inherently sustainable solution. Its environmental justification depends on high process yields, low-carbon energy supply, and transparent life cycle validation; otherwise, claims of circularity may overstate the actual benefits and contribute to greenwashing.

### 3.8. Future Directions

Although renewable and recyclable polymer systems have progressed significantly, unresolved challenges related to performance optimization, cost competitiveness, and waste management infrastructure continue to limit large-scale substitution of fossil-based plastics. Future research and implementation strategies must, therefore, adopt an integrated approach combining materials innovation, technological development, regulatory alignment, and systemic circularity design.

#### 3.8.1. Industrial Scalability and Technological Maturation

Scaling sustainable polymer production requires technological advancements that reduce energy intensity, improve process efficiency, and enhance material performance under real-world conditions. In sectors, such as automotive and packaging, lightweight biofiber-reinforced composites and CO_2_-based polymers are increasingly investigated as alternatives to petroleum-derived plastics, particularly where weight reduction and recyclability are strategic priorities [90,100].

However, broader industrial adoption depends on cost stabilization, consistent material quality, and compatibility with existing manufacturing infrastructure. Continued research into low-energy polymerization pathways, advanced compounding strategies, and automation-driven production systems is essential to achieve competitive parity with conventional polymers.

#### 3.8.2. Policy Harmonization and Regulatory Frameworks

Regulatory intervention plays a decisive role in accelerating sustainable polymer adoption. Policy initiatives, such as the European Green Deal, the United States Plastic Pollution Reduction Act, and Japan’s Plastic Resource Circulation Strategy establish precedents for restricting single-use plastics, mandating recycled content targets, and incentivizing bio-based material development [42].

Nevertheless, global regulatory fragmentation remains a structural barrier. Divergent certification standards, inconsistent labeling schemes, and region-specific compliance requirements complicate cross-border production and commercialization. Future progress requires harmonized sustainability metrics, standardized certification frameworks, and coordinated investments in recycling and composting infrastructure to ensure consistent implementation across markets [42].

#### 3.8.3. Circular Economy Integration and Advanced Recycling Systems

Beyond primary material substitution, sustainable polymer systems must be embedded within circular economy models emphasizing closed-loop recycling, resource recovery, and material traceability. Emerging research highlights the importance of integrating advanced chemical recycling, enzymatic depolymerization, and digital monitoring systems to enhance polymer recovery efficiency and maintain material value across multiple life cycles [38,101,102].

In addition, interdisciplinary approaches combining synthetic biology, AI-assisted material design, and renewable feedstock engineering are expected to further optimize polymer functionality while reducing environmental burdens. Such developments may improve scalability and enable the design of polymers specifically tailored for recyclability or controlled biodegradation pathways [38,102].

#### 3.8.4. Societal and Market Transition

Consumer perception, corporate sustainability commitments, and transparent communication of material properties will also influence transition dynamics. Misinterpretation of biodegradability claims or improper disposal practices may undermine environmental performance. Consequently, standardized labeling systems and evidence-based communication strategies are required to align consumer behavior with material design intent. Long-term transition requires coordinated progress across scientific innovation, industrial scaling, infrastructure development, and policy alignment. While material science advancements provide foundational improvements, systemic transformation toward circular polymer systems requires integration of technological, regulatory, and socio-economic dimensions. Strengthening collaboration among researchers, industry stakeholders, and policymakers will be critical to transforming sustainable plastics from emerging alternatives into mainstream industrial solutions [38,90,100,102].

## 4. Implications for Stakeholders

The transition toward sustainable polymer systems requires coordinated action across scientific, industrial, regulatory, and societal domains.

### 4.1. Research Community

Researchers play a central role in optimizing mechanical performance, degradation behavior, and processing stability of bio-based polymers. Molecular design, reinforcement, interface engineering, and predictive modeling should be evaluated together with durability and service-life requirements [15,37,42,103,104]. Standardized life cycle methodologies and transparent reporting are equally important for credible comparison, while scalable chemical and enzymatic recycling pathways require independent energy, yield, and economic validation [8,22,105].

### 4.2. Industry Professionals

Industrial stakeholders should evaluate sustainable polymers through a systems perspective encompassing cost, processing compatibility, supply-chain resilience, service-life requirements, and end-of-life integration. Collaboration between material producers, converters, product manufacturers, and waste-management operators is needed to align material design with actual recovery infrastructure. Table 11 summarizes the principal implementation challenges and evidence-informed response strategies discussed in the reviewed literature.

### 4.3. Policymakers

Policymakers influence adoption through harmonized certification standards, extended producer responsibility frameworks, and economic incentives aligned with life cycle evidence [7,94]. Global regulatory alignment would reduce uncertainty and enable cross-border scalability of sustainable polymer systems.

### 4.4. Consumers and Civil Society

Consumer behavior influences the realized environmental performance of recyclable and compostable polymers because disposal errors can contaminate recovery streams. Clear, standardized labeling and collection instructions are, therefore, necessary; however, behavioral measures must be coupled with accessible recovery infrastructure and material-compatible policy instruments [7,16,105].

## 5. Discussion

### 5.1. System Context, Life Cycle Trade-Offs, and Evidence Strength

Across the reviewed evidence, polymer sustainability is conditional rather than material-intrinsic. Production-stage climate results vary with electricity carbon intensity, feedstock and land-use assumptions, allocation rules and biogenic-carbon treatment, while end-of-life performance depends on collection, sorting, recycling/composting capacity and contamination control [7,8,16,17]. The source-linked synthesis, therefore, separates production-stage LCA evidence from application- and infrastructure-specific interpretation rather than repeating a single system-dependence conclusion across multiple subsections.

### 5.2. Recyclability vs. Biodegradability: A Structural Trade-Off

Recyclability and biodegradability are distinct end-of-life attributes. PET, HDPE, and PP can benefit from established mechanical-recycling systems where collection and sorting are effective [7,8,22], whereas compostable and biodegradable polymers require defined environmental conditions and compatible treatment routes [16,28,31]. The relevant comparison is, therefore, pathway compatibility for a specified application and region, not a general preference for one attribute.

### 5.3. Multi-Indicator Reality: Carbon Is Not Enough

Carbon footprint is the most consistently reported indicator, but it does not capture all trade-offs. Agricultural feedstocks may reduce fossil-carbon demand while increasing land, water, eutrophication or acidification burdens; product-level functional differences can change the amount of material required. Multi-indicator and function-based assessment is, therefore, necessary when sufficient data are available [16,17].

### 5.4. Evidence Strength and Methodological Dispersion

Although the bibliometric growth of sustainable polymer research has been exponential since 2017, methodological convergence remains limited. Significant heterogeneity exists in:

- Functional units (kg polymer vs. product-based units);
- System boundaries (cradle-to-gate vs. cradle-to-grave);
- Allocation approaches in bio-refineries;
- Treatment of biogenic carbon;
- End-of-life scenario modeling.

Only a subset of studies provides directly comparable functional units, boundaries, allocation rules and sensitivity analyses. For this reason, no normalized 0–100 framework is used; quantitative values are retained in their original units and categorical evidence is analyzed separately. Confidence is highest when source boundaries and assumptions are explicit and declines as comparisons require additional harmonization or regional extrapolation.

Accordingly, cross-material conclusions are stated only within the boundary and infrastructure conditions supported by the underlying evidence.

### 5.5. Economic Feasibility and Scale Constraints

Environmental performance cannot be decoupled from techno-economic feasibility. Several bio-based polymers remain cost-intensive due to fermentation yields, purification requirements, and limited economies of scale. Agricultural feedstock dependency introduces variability linked to land-use competition and geopolitical risk. Technologies, such as CO_2_-based polymerization and enzymatic depolymerization, show promising laboratory-level performance but remain limited in industrial maturity. Thus, environmental viability without economic scalability does not constitute engineering feasibility.

Future research should prioritize integrated LCA–TEA modeling frameworks, enabling simultaneous evaluation of environmental performance and economic feasibility under realistic deployment scenarios.

Engineered plastic-degrading microorganisms, including P450-driven synthetic bacterial systems, represent a complementary route toward biologically mediated depolymerization and upcycling [106].

### 5.6. Integration with Techno-Economic and Infrastructure Evidence

Environmental advantages are more credible when they remain compatible with manufacturing requirements, end-of-life infrastructure, and economic scale-up. The TEA evidence is thinner than the LCA evidence, particularly for PHA, PBS, and early-scale PEF; thus, cost and maturity conclusions are graded more cautiously. This asymmetry is, in itself, an important evidence gap; material innovation is extensively studied, while deployment conditions are less consistently quantified.

- Polymer chemistry aligns with recycling or composting infrastructure;
- Production is powered by low-carbon energy;
- Collection and sorting systems maintain material purity;
- Economic structures support scale deployment;
- Regulatory frameworks provide harmonized certification and incentives.

Isolated material innovation, without systemic synchronization, risks producing symbolic sustainability rather than structural transformation.

### 5.7. From Material Innovation to System Engineering

Future research should integrate application-specific functional units, equipment-level manufacturing data, service-life performance, LCA–TEA coupling, explicit end-of-life scenarios, and infrastructure-readiness metrics. These elements are treated as a common systems-engineering agenda rather than repeated as separate claims of system dependence.

- Coupled LCA + techno-economic assessment modeling;
- Infrastructure-readiness metrics embedded into material development;
- Design-for-recycling principles at molecular and additive levels;
- Dynamic material flow modeling under realistic circular scenarios;
- Harmonized global sustainability reporting protocols.

The transition toward circular polymer systems is not a chemistry problem alone—it is a systems engineering challenge.

### 5.8. Regional Variability and System-Dependent Performance

Regional variation is handled as a boundary condition rather than as a separate material ranking. Electricity mix, collection efficiency, recycling/composting capacity, and disposal practice can alter comparative results [2,7,8,16]; therefore, conclusions should be recalculated or reinterpreted for the deployment region instead of extrapolated globally.

### 5.9. Evidence Strength Assessment

Despite the exponential growth of sustainable polymer research, the robustness and comparability of the evidence base remain heterogeneous. While greenhouse gas (GHG) performance is the most consistently reported indicator across studies, methodological dispersion significantly affects cross-material comparability.

Approximately one-third of the reviewed studies apply multi-indicator life cycle assessment (LCA) frameworks with transparent system boundaries, allocation procedures, and sensitivity analyses. However, a substantial proportion relies on cradle-to-gate boundaries without realistic end-of-life modeling, limiting conclusions regarding full circular performance. Differences in functional units (mass-based vs. product-based), treatment of biogenic carbon, regional electricity mixes, and land-use change assumptions introduce further variability.

Confidence in comparative carbon footprint results can, therefore, be considered moderate, particularly when system boundaries and energy assumptions are explicitly reported. In contrast, confidence in cross-material comparisons involving multi-impact indicators (eutrophication, water use, land occupation, acidification) remains limited, due to inconsistent reporting and indicator selection.

Techno-economic assessment (TEA) integration is underrepresented relative to environmental modeling. Only a minority of studies combine LCA and economic feasibility analysis within harmonized system boundaries. As a result, confidence in scalability projections and industrial competitiveness assessments remains weak to moderate.

Infrastructure-dependent outcomes, such as compostability and recycling performance, are inherently context-specific. Evidence strength in these domains is, therefore, best described as conditional, requiring regional validation rather than global generalization.

To separate evidence strength from interpretation, conclusions were graded using a four-level confidence scheme: high, moderate, weak-to-moderate, and conditional (Table 12). The grading considered the number of studies, consistency of reported ranges, comparability of system boundaries, and sensitivity to regional infrastructure assumptions.

The relationship between the principal conclusions, their supporting evidence, methodological limitations, and assigned confidence levels is documented in Supplementary Table S5, providing an explicit evidence-to-claim traceability framework for the overall assessment.

This grading reduces the risk of overgeneralization. Conclusions supported primarily by harmonized carbon-footprint data are expressed with moderate confidence, whereas conclusions involving infrastructure behavior, leakage, composting availability, or cost competitiveness are framed as conditional or weak-to-moderate.

Overall, evidence confidence is moderate for well-defined carbon-footprint comparisons, weak-to-moderate for techno-economic scalability, and conditional for infrastructure-dependent outcomes. Table 12 records these boundaries explicitly.

No additional global ranking is inferred beyond the confidence-graded conclusions in Table 12.

The resulting research–implementation gap is defined by strong material-level evidence but comparatively sparse deployment-level quantification.

### 5.10. Integrated Interpretation: From Juxtaposition to Analytical Coupling

The combined evidence indicates that the central claim of system dependence is supported by three linked observations rather than by assertion alone. First, bibliometric mapping shows that material innovation, renewable resources, and mechanical properties dominate the literature, while infrastructure, TEA, and scenario-based end-of-life evidence remain less visible. Second, the LCA synthesis shows that environmental rankings shift with the energy mix, allocation method, land-use assumptions, and end-of-life scenario. Third, the TEA and infrastructure screening shows that polymers with promising environmental profiles may be constrained by cost, capital intensity, or lack of compatible recovery systems.

The analytical pipeline, therefore, links research attention to quantitative environmental evidence, economic feasibility, and infrastructure readiness without collapsing these dimensions into a single score.

The principal limitations are heterogeneity in functional units, inventory databases, allocation rules, regional energy systems, biodegradation protocols, and techno-economic assumptions. Google Scholar was supplementary because its ordering and coverage are less reproducible than curated databases. The synthesis preserves quantitative indicators in their original units and treats categorical evidence separately. TEA remains semi-quantitative because harmonized cost and scale data are sparse.

## 6. Conclusions

The findings are directly supported by the reviewed dataset. Across the 193 included studies, quantitative LCA evidence most consistently supports the conclusion that climate performance varies with energy mix, feedstock assumptions, allocation, and end-of-life treatment. Bibliometric-to-evidence mapping further shows that material innovation and mechanical/processing topics receive substantially greater attention than explicit TEA and scenario-based end-of-life modeling. The confidence analysis, therefore, supports moderate confidence for harmonized carbon-footprint comparisons, weak-to-moderate confidence for economic scalability claims, and conditional confidence for infrastructure-dependent outcomes.

Interpretation is derived from heterogeneous evidence. Taken together, the evidence indicates that polymer sustainability is system-dependent: relative performance can change with formulation, processing energy, service life, collection efficiency, recovery infrastructure, regional electricity, and production maturity. These dimensions are not converted into an aggregate numerical ranking.

The recommendations proposed by the authors are as follows. Future engineering studies should combine application-specific functional units with coupled LCA–TEA analysis, report manufacturing energy and yield at equipment level, include service-life or durability normalization for engineering components, model explicit end-of-life scenarios, and state industrial maturity separately from laboratory feasibility. Harmonized reporting of search protocols, LCA assumptions, infrastructure conditions, and uncertainty would make future polymer comparisons more reproducible and more useful for circular economy decision-making.

## Figures and Tables

**Table 1:** Positioning of the present review against representative recent reviews.

Review	Core Scope	LCA/Quantitative Synthesis	Manufacturing and Functional Performance	TEA/Infrastructure/EoL	Position Relative to Present Review
Lazăr et al. 2023 [15]	Eco-design of polymer-matrix composite parts	Environmental considerations, not a harmonized cross-polymer LCA synthesis	Strong emphasis on design, manufacturing, performance, life span and recycling	Limited explicit TEA and regional infrastructure coupling	Foundation for eco-design; present review adds systematic evidence coupling, LCA/TEA scenarios and confidence grading
Shamsuyeva and Endres 2021 [7]	Circular economy and plastics recycling	Contextual environmental evidence	Processing/recycling focus	Strong recycling-system context; limited cross-material TEA coupling	Present review links recycling evidence to LCA, EoL archetypes and material-specific trade-offs
Mushtaq et al. 2024 [6]	Green-plastics breakthroughs, applications, future pathways	Broad environmental discussion	Broad technology coverage	Barriers and applications discussed qualitatively	Present review adds reproducible screening, evidence-gap quantification and scenario sensitivity
Rosenboom et al. 2022 [16]	Bioplastics for circular economy	life cycle perspective	Material/function perspective	Circularity and EoL emphasized	Present review adds bibliometric mapping, quantitative LCA/TEA comparison and maturity/confidence framework
Ali et al. 2023 [17]	Bioplastic production and LCA	Strong LCA focus	Production covered mainly through LCA inventory perspective	EoL discussed; less emphasis on service life and industrial maturity	Present review broadens from LCA to coupled engineering performance, infrastructure and TEA
Velasquez et al. 2025 [18]	Biobased/biodegradable polymer chemistry, society, environmental implications	Updated LCA and environmental synthesis	Chemistry/function integrated conceptually	EoL, markets, regulation and societal aspects	Present review adds PRISMA-screened cross-polymer evidence mapping, explicit scenarios, scenario-based robustness analysis and confidence grading
Present review	System-dependent sustainable polymer technologies	Stratified quantitative LCA ranges + scenario-based robustness analysis	Manufacturing variables, experimental integrated LCA, service-life framework	TEA clustering, maturity, EoL archetypes, infrastructure and evidence confidence	Analytical coupling of evidence streams to identify when comparative performance and claims are conditional

**Table 2:** Inclusion and exclusion criteria for article selection.

Criterion	Inclusion Criteria	Exclusion Criteria
Timeframe	Articles published between 1998 and 2024, covering three decades of advancements in sustainable plastics and capturing contemporary developments in the field.	Articles published before 1998 or after 2024, as they fall outside the defined scope of the review.
Language	Publications in English, ensuring accessibility and reliability of interpretation. Non-English studies are included only if high-quality translations are available.	Non-English publications without reliable translations, to maintain clarity and consistency in data interpretation.
Publication Type	Peer-reviewed journal articles, books, and full peer-reviewed conference proceedings when they reported transparent methods, data, and sustainability indicators.	Non-peer-reviewed works, opinion pieces, editorials, abstracts, or conference papers lacking sufficient methodological validation or extractable data.
Sustainability Metrics	Research explicitly addressing sustainability metrics, including carbon footprint, recyclability, biodegradability, resource efficiency, or life cycle assessments (LCA).	Studies without reference to sustainability metrics or focused exclusively on conventional plastics without environmental implications.
Focus and Scope	Studies offering substantive insights into material innovations, green manufacturing processes, environmental impacts, life cycle comparisons, techno-economic constraints, or regional infrastructure conditions relevant to sustainable and conventional plastics.	Research lacking relevance to the scope of the review, or highly localized studies without transferable parameters, transparent assumptions, or relevance to system-dependence analysis.
Applications Covered	Articles spanning diverse applications, including packaging, automotive, biomedical devices, construction, agriculture and other industrial sectors to ensure a comprehensive perspective.	Studies limited to niche applications or those with insufficient relevance to broader industry practices or sustainability challenges.
Methodological Transparency	Research with clear and replicable methodologies, detailing experimental setups, data collection, and analysis techniques, ensuring robust and valid conclusions.	Articles that fail to provide adequate methodological transparency or lack reproducibility, compromising the validity of their findings.

**Table 3:** Operational linkage between bibliometric clusters and LCA/TEA/infrastructure evidence.

Bibliometric Theme	Linked Evidence Category	Observed Integration Role in the Synthesis	Evidence Gap Interpretation
Renewable resources/biopolymers	Bio-based feedstocks, LCA carbon and land-use assumptions	Directly linked to GHG ranges for PLA, PHA, PBS, and PEF; also linked to land-use and agricultural-input uncertainty.	Moderate: climate evidence is relatively mature, but multi-indicator and regional land-use evidence remains uneven.
Mechanical properties/composites	Engineering performance and durability	Linked to processing constraints, recycling-cycle degradation, and application suitability for durable goods.	Moderate: strong materials evidence, weaker connection to full life cycle performance.
Recycling/circular economy	EoL scenarios, infrastructure compatibility, contamination risk	Linked to comparative interpretation of PET/HDPE/PP and PEF performance under high collection and sorting rates.	Low-to-moderate: strong conceptual evidence, but inconsistent scenario assumptions.
Biodegradability/compostability	Composting infrastructure, leakage, landfill behavior	Linked to conditional benefits of PLA/PHA/PBS under controlled composting and leakage-prone systems.	High: many claims are material-centric, while infrastructure availability is often under-modeled.
Techno-economic feasibility/scalability	Cost ranges, capital intensity, learning potential	Linked to production-cost multiples and maturity constraints	High: TEA evidence is sparse relative to environmental LCA evidence.
Policy/governance	Regulatory alignment, certification, consumer sorting behavior	Linked to adoption barriers and regional implementation constraints.	Moderate-to-high: policy discussion is common, but quantitative coupling with LCA or TEA is limited.

**Table 4:** Source-linked environmental-performance evidence without cross-criterion normalization.

Material	Carbon Footprint (Production-Stage Evidence)	Biodegradability (Conditions)	Recyclability (Current Systems)	Resource-Efficiency Evidence
PLA (Polylactic Acid)	Production-stage LCA literature reports substantial scenario dependence; electricity mix, agricultural inputs, allocation, and biogenic-carbon treatment materially affect results [16,17,38].	Industrial compostability is achievable under defined controlled conditions; ambient degradation is slower and environment-dependent [28,31].	Technically recyclable, but dedicated collection, sorting, contamination control, and sufficient stream purity remain limiting [7,39].	Renewable agricultural feedstocks can reduce fossil dependence, but fertilizer, land-use, water, and process-energy assumptions must be included [16,17].
PHA (Polyhydroxyalkanoates)	Cradle-to-gate results are highly route-dependent because feedstock, fermentation productivity, aeration, downstream recovery, and allocation differ among studies [17,38,40,41].	Biodegradation has been demonstrated in selected biologically active environments, but rate and completeness depend on polymer composition and test conditions [30,40].	Established mechanical-recycling infrastructure is limited; PHA is more often evaluated through biodegradation or alternative recovery pathways [39].	Bio-based fermentation can use renewable or waste-derived feedstocks, but yield and downstream recovery strongly influence resource demand [40,41].
PBS (Polybutylene Succinate)	Published environmental results differ by precursor route, electricity/heat supply, allocation, and modeled scale; a universal resin-level value is therefore not reported [17,31,38].	Compostable/biodegradable under defined conditions; performance depends on formulation, temperature, moisture, microbial activity, and residence time [31].	Dedicated recycling evidence and infrastructure remain limited compared with PET, PP, or HDPE [39].	Bio-based precursor routes may reduce fossil feedstock use but can introduce fermentation, purification, and separation burdens [17,38].
PEF (Polyethylene Furanoate)	PEF is reported as a potentially lower-impact bio-based polyester in comparative assessments, but results remain sensitive to FDCA route, process scale, allocation, and energy supply [16,38,42].	Non-biodegradable; recovery therefore depends on recycling or controlled waste management.	Potential compatibility with PET-oriented recovery is promising, but sorting, contamination, and scale-specific validation remain important [16,42].	Bio-based FDCA can reduce fossil feedstock dependence; conversion efficiency and energy demand remain important determinants [38,42,43].
Chitosan-based Polymers	Evidence is predominantly application-specific and is not sufficiently harmonized for a single resin-level carbon-footprint value [44,45,46].	Biodegradable in biologically active environments; formulation and crosslinking influence degradation [44,46].	Generally not integrated into conventional mechanical-recycling streams.	Can valorize crustacean-shell biomass; preprocessing, deacetylation, purification, and application formulation affect resource demand [44,47].
Starch-based Bioplastics	Results vary with blend composition, agricultural assumptions, plasticizer/additive system, and product function [16,17,48].	Compostability and soil degradation depend on formulation and environmental conditions [31,48].	Generally not compatible with conventional PET/polyolefin mechanical-recycling streams unless specifically segregated.	Renewable feedstock, but agricultural land, fertilizer, water, and processing requirements must be considered [16,17].
Cellulose-based Bioplastics	Resin, fiber, derivative, coating, and composite systems use heterogeneous boundaries and functional units; no single range is imposed [42,49,50].	Biodegradation depends on chemical modification, coating, additives, and the test environment [46,50].	Compatibility with existing recycling depends on material form, additives, and composite architecture.	Renewable forestry/cellulosic feedstocks can support circularity, but forestry management and conversion burdens remain relevant [49,50].

**Table 5:** Engineering comparison framework for polymer manufacturing routes. Quantitative values should be reported at the equipment and product-system level because cross-study energy and cycle-time data are not harmonized across materials and scales.

Process	Measurable Engineering Parameters	Main Energy/GHG Drivers	Yield, Scrap, Solvent	Scale/Equipment Implications	Industrial Maturity and Evidence Boundary
Extrusion	Barrel temperature, screw speed, torque/SME, residence time, moisture	Heaters, motor load, drying	Continuous; start-up/off-spec scrap; usually solvent-free	High-throughput continuous line; retrofit often feasible	Commercial; strong process evidence, but polymer-specific energy varies
Injection molding	Melt/mold temperature, pressure, cycle time, reject rate	Plasticization, clamping, cooling, drying	Sprues/rejects; typically solvent-free	High-volume tooling and dryers; parameter-sensitive for hydrolysis-prone biopolymers	Commercial; comparison should use kWh/kg or kWh/part plus property retention
Blow molding/thermoforming	Melt/reheat temperature, cooling time, wall-thickness control, cycle time	Reheating, compressed air, cooling	Trim/start-up waste; typically solvent-free	Commercial packaging equipment; material-specific window	Commercial; barrier and thickness reduction must be included in functional unit
Compression/composite consolidation	Platen/autoclave temperature, pressure, cure/consolidation time	Heating, pressure/vacuum, cure duration	Trim waste, prepreg/resin losses; route-dependent solvents	Tooling and consolidation equipment can be capital intensive	Commercial for composites; energy strongly depends on cure route and batch utilization
Reactive extrusion	Temperature profile, torque/SME, conversion, residence time, devolatilization	Mechanical energy, heating, vacuum/devolatilization	Potentially solvent-free; conversion/off-spec losses important	Continuous reactive extruder; chemistry-specific retrofit	Pilot-to-commercial depending on chemistry; compare conversion and purification burden
Biocatalytic synthesis	Temperature, enzyme loading, conversion, reaction time, water/solvent ratio	Agitation, temperature control, enzyme production, downstream recovery	Potentially mild conditions but purification can dominate	Bioreactor and separation train; scale-up mass-transfer constraints	Laboratory-to-pilot for many routes; economic evidence limited
CO_2_-based polymerization	Pressure, temperature, catalyst loading, CO_2_ utilization, conversion	Gas compression/purification, pressure, separation	Catalyst and separation losses; solvent route-dependent	Specialized pressure equipment and CO_2_ supply	Pilot-to-commercial depending on chemistry; climate benefit must be LCA-verified
Additive manufacturing (FFF)	Nozzle/bed temperature, print speed, build time, failed-print rate	Electricity per part and feedstock conditioning	Low tooling waste but failed prints/supports can be material	Flexible, low-volume; poor economics for many high-throughput parts	Commercial for prototyping/specialized production; part-level FU required
Electrospinning	Voltage, flow rate, collector speed, solvent concentration, throughput	HV supply, ventilation, solvent recovery, climate control	Solvent recovery and low throughput are critical	Specialized line; scale-up requires multi-jet/needleless systems	Laboratory-to-industrial niche; cross-study energy data remain sparse

**Table 6:** Methodological stratification of LCA studies included in the review.

Category	Sub-Category	Description/Relevance for Comparison
System Boundaries	Cradle-to-gate	Includes raw material extraction and polymer production; used as primary basis for harmonized comparison
System Boundaries	Cradle-to-grave	Includes full life cycle (production, use, end-of-life); requires adjustment for comparability
Functional Unit (FU)	Mass-based (e.g., kg polymer)	Enables direct material-level comparison across studies
Functional Unit (FU)	Product-based (e.g., packaging unit)	Reflects application context but introduces variability
Allocation Methods	Mass allocation	Based on physical output; commonly used in industrial LCA
Allocation Methods	Economic allocation	Based on market value; sensitive to price fluctuations
Allocation Methods	System expansion	Avoids allocation by expanding system boundaries; increases complexity
Impact Assessment Methods	IPCC (carbon footprint)	Focus on climate change (GHG emissions)
Impact Assessment Methods	ReCiPe/CML	Multi-indicator assessment (e.g., eutrophication, acidification)
End-of-Life (EoL) Modeling	Mechanical recycling	Assumes closed-loop or open-loop recycling scenarios
End-of-Life (EoL) Modeling	Chemical recycling	Includes depolymerization pathways; often energy-intensive
End-of-Life (EoL) Modeling	Composting/biodegradation	Requires controlled conditions; infrastructure-dependent
End-of-Life (EoL) Modeling	Landfill/incineration	Conventional disposal routes with varying environmental impacts
Geographic Context	Low-carbon energy systems	Renewable-dominant electricity mix; favors bio-based polymers
Geographic Context	High-carbon energy systems	Fossil-intensive grids; may offset environmental benefits
Geographic Context	Advanced waste infrastructure	High recycling/composting rates; enables circular performance
Geographic Context	Limited waste infrastructure	Mismanagement risks; reduces effectiveness of sustainable polymers

**Table 7:** Source-linked comparative LCA evidence. Reported ranges are synthesis envelopes from comparable production-stage studies; they are not universal polymer constants.

Polymer	Carbon Footprint (kg CO_2_e/kg)	Recyclability	Biodegradability	Energy Consumption (MJ/kg)
PLA	No single value is imposed. Comparative LCA literature reports scenario-dependent production-stage impacts governed by electricity, feedstock, allocation, and plant assumptions [16,17,38].	Limited/dedicated streams	Industrial composting under certified conditions	Energy demand is process- and boundary-dependent; fermentation, drying, polymerization, and conversion energy should be reported separately [17,38].
PHA	Not pooled into a universal value; results are strongly affected by substrate, fermentation productivity, aeration, recovery, and co-product treatment [17,40,41].	Limited	Context-dependent biodegradation; conditions must be specified	Fermentation and downstream recovery can be energy-intensive; route-specific reporting is required [38,40,41].
PBS	Not pooled. Results depend on bio-/fossil precursor route, modeled scale, allocation, and energy system [17,31,38].	Limited	Compostable/biodegradable under defined conditions	Energy performance is route-specific and should not be generalized across bio-PBS and fossil-PBS pathways [17,38].
PEF	Comparative studies and reviews indicate potential GHG reductions relative to fossil PET under favorable bio-based production assumptions, but the magnitude is route- and scale-dependent [16,38,42].	Potential	No	Energy demand depends on FDCA production, polymerization, scale, and electricity/heat supply [38,42,43].
PET	Use established resin-level LCA/LCI comparisons only within matched system boundaries; no global constant is imposed [2,7,8].	Widely	No	Mature industrial production; energy values remain dataset- and region-specific [2,8].
PP	Use established resin-level LCA/LCI comparisons only within matched boundaries; no global constant is imposed [2,7,8].	Widely	No	Mature industrial production; energy demand varies with feedstock and regional energy supply [2,8].
HDPE	Use established resin-level LCA/LCI comparisons only within matched boundaries; no global constant is imposed [2,7,8].	Widely	No	Mature industrial production; energy demand varies with process and energy-system assumptions [2,8].

**Table 8:** Integrated environmental and techno-economic indicators with direct source mapping. Economic values are retained only when they are traceable to a defined source and basis; heterogeneous cost evidence is not converted into universal polymer-to-polymer multipliers.

Polymer	Source-Linked Climate Evidence	Source-Linked Cost Evidence	Key Economic Constraints	System Compatibility
PLA	LCA evidence supports potentially lower production-stage GHG emissions under favorable energy/feedstock assumptions, but cross-study values are not pooled when boundaries differ [16,17,38].	Commercially established, but cost remains sensitive to feedstock, fermentation, purification, energy price, and plant scale; no universal cost multiple is claimed [38].	Feedstock price, fermentation efficiency, purification, energy use, scale.	Climate performance depends on energy mix and allocation; recovery route must be specified.
PHA	Environmental performance is route-specific because fermentation substrate, productivity, aeration, recovery, and allocation can dominate results [17,40,41].	Commercialization remains cost-constrained by feedstock, fermentation productivity, downstream recovery, product consistency, and scale; the literature does not justify one universal PHA/PET multiple [38,40,41].	Fermentation productivity, feedstock, downstream recovery, product consistency, scale.	Biodegradation is environment-dependent; recycling infrastructure is limited.
PBS	Environmental performance varies with precursor route, modeled scale, process energy, and end-of-life assumptions [17,31,38].	Cost conclusions are route- and scale-specific; the analysis uses a qualitative scenario discussion grounded in the literature [38].	Bio-based precursor cost, scale, energy demand, purification.	Composting/biodegradation benefits depend on treatment conditions and collection.
PEF	Comparative literature indicates potential environmental advantages over PET under favorable bio-based process assumptions, while scale-up remains a key uncertainty [16,38,42].	Cost evidence is still shaped by early-scale production, FDCA supply, capital intensity, and market development; no universal cost multiplier is retained [38,42].	FDCA/MEG supply, capital intensity, first-of-a-kind scale-up, market development.	Potential compatibility with PET-oriented collection/recycling requires sorting and contamination control.
PET	Conventional comparator; environmental values depend on geography, process dataset, recycled content, and system boundary [2,7,8].	Commodity baseline; regional grade, feedstock and energy prices vary, so no single global resin price is assigned.	Fossil feedstock exposure, decarbonization of production, circularity performance.	Mature collection/recycling in many markets, but actual recovery remains region-dependent.
PP	Conventional comparator; interpretation should use matched boundaries and regional data [2,7,8].	Commodity baseline; no single global price or cost multiple is assigned.	Fossil feedstock exposure, sorting and recycling economics.	Established processing base; recycling outcomes depend on collection and contamination.

**Table 9:** End-of-life scenario matrix for system-dependent polymer performance. The scenario archetypes are constructed by the authors from the evidence synthesized in the reviewed literature and are intended as evidence-informed analytical scenarios, not as recommendations reproduced from a single source.

EoL Archetype	Scenario Condition (Context-Dependent)	Key Assumptions	Better-Performing Polymers	Main Limitation
High recycling availability	High collection and sorting effectiveness; functioning secondary-material markets	Clean streams, controlled contamination, credible displacement of virgin resin	PET, HDPE, PP, PEF-compatible streams	Downcycling, additives, quality loss, collection variability
Industrial composting available *	Certified industrial composting is accessible and accepts the relevant products	Correct sorting; controlled temperature/humidity; product certification	PLA, PBS, selected starch blends, selected PHA	Facilities may reject compostable plastics; collection systems differ
Landfill/incineration dominant	Recycling and composting are limited; disposal or energy recovery dominates	Regional disposal practices and energy-recovery conditions control outcomes	No universal winner	Biodegradation may be slow in landfill; incineration loses material circularity
Leakage-prone system	High risk of mismanaged waste and environmental release	Persistence and degradation behavior become relevant beyond conventional LCA indicators	PHA and selected validated biodegradable systems may have relative advantages	Degradation conditions vary; leakage impacts are incompletely represented in many LCAs
Organic co-collection	Compostable items are intentionally collected with compatible organic-waste streams	Clear labeling, consumer sorting, facility acceptance and certified compostability	Selected compostable films and food-contact items	Requires coordinated collection and facility acceptance

* EN 13432 [95] requires disintegration within 12 weeks and biodegradation within six months under industrial composting conditions.

**Table 10:** Semi-quantitative techno-economic clustering of selected polymer systems. Cost ranges are representative contextual indicators retained from the techno-economic evidence summarized in Table 8 and should not be interpreted as a formal meta-analysis or price forecast. Maturity labels distinguish commercial maturity from laboratory, pilot, demonstration, or early commercial evidence and refer to the production routes represented in the reviewed literature rather than to a universal TRL for the polymer chemistry. * PEF estimates carry higher uncertainty because evidence is dominated by pre-commercial and first-of-a-kind scale-up assumptions.

System	Cost Evidence	Class	CapEx	Learning	Sustainability (System-Level)	Maturity Stage
PET/PP/HDPE	Commodity reference systems; prices vary by grade, region, feedstock and year. Mature production and recovery infrastructure provide the economic baseline [2,7].	Commodity	Low	Low	Dependent on decarbonized energy + effective recycling.	Commercial/mature
PLA	No universal multiplier retained; commercial cost is strongly dependent on feedstock, fermentation efficiency, energy price and plant scale [38].	Transition	Medium	Med–High	Climate benefit depends on energy mix; recovery pathway must be specified.	Commercial
PHA	No universal multiplier retained; commercialization remains constrained by fermentation productivity, downstream recovery and scale [38,40,41].	Niche	High	High	Biodegradation potential is conditional; cost and scale remain limiting.	Commercial niche; route-specific
PBS	No universal multiplier retained; economics depend on precursor route, process integration, purification and scale [38].	Specific	Med–High	Medium	Compostability is infrastructure-dependent.	Commercial/limited market
PEF	No harmonized multiplier retained; evidence remains sensitive to early-scale production, capital intensity, FDCA supply and market development [38,42].	Pre-commercial/early scale	High	High	Barrier performance and recycling integration are key system variables.	Demonstration/early commercial; uncertain
Natural polymers	Not harmonized; highly application-, feedstock- and processing-dependent [46,75,76].	Variable	Variable	Medium	Application- and feedstock-dependent.	Lab–commercial; application-specific

* early-stage estimate.

**Table 11:** Challenges and potential solutions for industrial adoption of sustainable plastics. The potential solutions are author-synthesized recommendations derived from the reviewed evidence rather than verbatim proposals from individual source papers.

Key Challenges in Industrial Adoption	Potential Solutions
High production costs	Waste-fed microbial bioreactors, CO_2_-based polymerization
Limited mechanical properties	Nanocomposite reinforcement, AI-driven polymer optimization
Incompatibility with traditional processing	Low-energy twin-screw extrusion, AI-assisted injection molding
Waste management complexities	Enzymatic depolymerization, advanced chemical recycling

**Table 12:** Confidence grading of major conclusions.

Conclusion	Evidence Base	Confidence Level	Interpretive Boundary
PLA can reduce GHG emissions relative to PET under low-carbon production assumptions	Multiple LCA studies with reported kg CO_2_e/kg ranges	Moderate	Depends on electricity mix, land-use change, allocation, and functional unit.
PHA offers strong biodegradation potential in selected environments	Biodegradation and LCA literature, but heterogeneous conditions	Conditional-to-moderate	Benefits require suitable environmental or managed degradation conditions and cost-effective production.
Recyclable polymers often outperform biodegradable alternatives in mature recovery systems	LCA and circularity evidence for PET, HDPE, PP and emerging PEF	Moderate	Requires high collection, sorting quality, and displacement of virgin resin.
Compostable polymers deliver benefits only where composting infrastructure is available	EoL and infrastructure literature; uneven regional reporting	Conditional	Invalid where composting facilities are absent, reject packaging, or sorting is poor.
Economic scalability remains a limiting factor for PHA, PBS, and early-stage PEF	Sparse TEA data, cost multiples, and industrial maturity indicators	Weak-to-moderate	Cost assumptions vary by feedstock, scale, energy price, and policy support.
The overall sustainability ranking is system-dependent rather than material-intrinsic	Convergent evidence across LCA ranges, TEA indicators, bibliometric gaps, and EoL scenarios	Moderate	Relative ranking may change across regions, infrastructure, and modeling assumptions.

## Data Availability

The methodological documentation supporting the literature search, evidence harmonization, and quantitative synthesis is provided in Appendix A (Table A1, Table A2 and Table A3). Additional information regarding bibliographic traceability, source-linked LCA evidence, harmonization procedures, techno-economic constraints, and evidence-confidence assessment is provided in the Supplementary Materials (Tables S1–S5).

## Supplementary Materials

The following supporting information can be downloaded at: https://www.mdpi.com/article/10.3390/polym18202467/s1, Table S1: Manuscript reference inventory and systematic-dataset traceability; Table S2: Source-linked evidence supporting the comparative LCA synthesis; Table S3: Evidence harmonization and methodological comparability criteria; Table S4: Source-linked techno-economic evidence and scalability constraints; Table S5: Evidence-to-claim traceability and confidence assessment.

## Appendix A. Reproducibility Documentation

**Table A1:** Database-specific search protocol and retrieval counts.

Database	Fields/Syntax	Complete Search Logic (1998–2024; English)	Records
Scopus	TITLE-ABS-KEY	TITLE-ABS-KEY((“sustainable plastics” OR “sustainable polymers”) OR ((“biopolymers” OR “green polymers” OR “natural-based plastics”) AND “renewable resources”) OR ((“green manufacturing” OR “sustainable manufacturing”) AND (polymers OR plastics)) OR ((“life cycle assessment” OR LCA) AND (“environmental impact” OR “economic viability”) AND “sustainable polymers”) OR ((scalability OR “adoption barriers”) AND “industrial integration” AND “sustainable plastics”) OR ((challenges OR limitations) AND “sustainable plastics”) OR (“circular economy” AND innovations AND “sustainable polymers”)) AND PUBYEAR > 1997 AND PUBYEAR < 2025	39,672
Web of Science Core Collection	Topic (TS)	TS = ((“sustainable plastics” OR “sustainable polymers”) OR ((biopolymers OR “green polymers” OR “natural-based plastics”) AND “renewable resources”) OR ((“green manufacturing” OR “sustainable manufacturing”) AND (polymers OR plastics)) OR ((“life cycle assessment” OR LCA) AND (“environmental impact” OR “economic viability”) AND “sustainable polymers”) OR ((scalability OR “adoption barriers”) AND “industrial integration” AND “sustainable plastics”) OR ((challenges OR limitations) AND “sustainable plastics”) OR (“circular economy” AND innovations AND “sustainable polymers”)); timespan 1998–2024; English	34,116
PubMed	Title/Abstract	((“sustainable plastics”[Title/Abstract] OR “sustainable polymers”[Title/Abstract]) OR ((biopolymers[Title/Abstract] OR “green polymers”[Title/Abstract] OR “natural-based plastics”[Title/Abstract]) AND “renewable resources”[Title/Abstract]) OR ((“green manufacturing”[Title/Abstract] OR “sustainable manufacturing”[Title/Abstract]) AND (polymers[Title/Abstract] OR plastics[Title/Abstract])) OR ((“life cycle assessment”[Title/Abstract] OR LCA[Title/Abstract]) AND (“environmental impact”[Title/Abstract] OR “economic viability”[Title/Abstract]) AND “sustainable polymers”[Title/Abstract]) OR ((scalability[Title/Abstract] OR “adoption barriers”[Title/Abstract]) AND “industrial integration”[Title/Abstract] AND “sustainable plastics”[Title/Abstract]) OR ((challenges[Title/Abstract] OR limitations[Title/Abstract]) AND “sustainable plastics”[Title/Abstract]) OR (“circular economy”[Title/Abstract] AND innovations[Title/Abstract] AND “sustainable polymers”[Title/Abstract])) AND (1998:2024[pdat]) AND English[lang]	8947
Google Scholar	Supplementary discovery; seven thematic queries	The seven thematic Boolean blocks reported in Section 2.2.2 were run separately because Google Scholar does not provide stable field-tag behavior equivalent to Scopus/WoS. Results were retrieved with the dedicated retrieval tool used in the original search and restricted during screening to 1998–2024, English-language peer-reviewed records.	38,713

Search-date note: the final systematic search was completed in July 2025. The exact day was not retained in the original export log and is therefore not reconstructed. This is reported as a protocol limitation. Deduplication and agreement: exact DOI/PMID/persistent-identifier matches were removed first, followed by normalized title + first-author + year matching. Near-duplicates were manually adjudicated. Two reviewers screened independently and resolved disagreements by consensus; a prospective κ statistic was not recorded.

**Table A2:** Rules for harmonizing LCA studies to the 1 kg polymer-at-factory-gate comparison.

Source-Study Situation	Treatment in the Present Synthesis
Cradle-to-gate, 1 kg resin/polymer at factory gate	Included directly if allocation and biogenic-carbon treatment were reported or interpretable.
Cradle-to-grave with separately reported production stage	Only the reported production-stage contribution was eligible for the mass-based synthesis; EoL/use contributions remained separate.
Cradle-to-grave total without separable stages	Excluded from production-stage pooling; retained for scenario/application interpretation. No back-calculation by subtracting assumed EoL values was performed.
Product-based functional unit with explicit polymer mass and equivalent function	Converted only when the study itself provided sufficient mass/function information; otherwise kept in an application-specific stratum.
Incompatible allocation, unreported biogenic-carbon treatment, or non-separable boundary	Not pooled quantitatively; used qualitatively if methodologically informative.
Energy indicator	Retained in MJ/kg only when the source defined primary/fossil/cumulative energy clearly enough for comparison; otherwise not merged.

**Table A3:** Minimum study-level extraction fields used to audit quantitative LCA/TEA statements.

Field	Recorded Information
Bibliographic source	Author, year, DOI/reference number
Material/grade	PLA, PHA, PBS, PEF, PET, PP, HDPE; grade/formulation when available
Functional unit	Original FU and any justified conversion
System boundary	Cradle-to-gate, cradle-to-grave, or application-specific
Geography/electricity	Region and electricity-mix assumption when stated
Allocation/biogenic carbon	Mass/economic/system expansion; carbon-accounting convention when stated
GHG result	kg CO_2_e per original FU and harmonized value only when eligible
Energy result	MJ per original FU with energy definition
TEA result	Production cost/MSP, currency-year, plant scale, CAPEX/OPEX assumptions when reported
Use in synthesis	Included in quantitative range/scenario interpretation/excluded from pooling, with reason
