# Supplementary material for: Sustainable Polymers as System-Dependent Technologies: A Critical Engineering Assessment of LCA Evidence, Circular Compatibility, and Scalability Constraints
Source: Polymers (Basel). 2026 Oct 9;18(20):2467. doi: 10.3390/polym18202467 (PMC13708781; doi:10.3390/polym18202467)
Supplement: Supplementary file 1 [file polymers-18-02467-s001.zip › polymers-4552561-supplementary.pdf]

Supplementary Information S1–S5

for "Sustainable Polymers as System-Dependent Technologies: A Critical Engineering Assessment of LCA Evidence, Circular Compatibility, and Scalability Constraints"

Scope and transparency note. This supplementary file is synchronized with the current revised manuscript. It does not reconstruct study-level data that are absent from the manuscript or the retained screening/export files. The systematic dataset is reported as n = 193 for the 1998–2024 search window, whereas the manuscript reference list contains 103 cited sources and also includes targeted post-cutoff contextual updates. Accordingly, the tables below distinguish manuscript-reference traceability from systematic-dataset traceability and avoid treating bibliography order as a surrogate for the 193-study screening registry.

Table S1. Manuscript reference inventory and systematic-dataset traceability

Table S1 inventories all 103 references in the current manuscript in citation order. "Systematic-window eligible by year" means only that the publication year falls within 1998–2024; it does not prove that the record belonged to the final n = 193 screened dataset. Post-2024 sources are explicitly marked as contextual updates outside the systematic search window. Membership in the n = 193 dataset must be verified against the original screening/export registry.

Manuscript ref.	Bibliographic record	DOI	Year	Traceability status
1	Geyer, R.; Jambeck, J.R.; Law, K.L. Production, use, and fate of all plastics ever made. Sci. Adv. 2017, 3, e1700782. https://doi.org/10.1126/sciadv.1700782	10.1126/sciadv.1700782	2017	Within systematic search window by publication year; dataset membership requires original screening registry
2	Organisation for Economic Co-operation and Development (OECD). Global Plastics Outlook: Economic Drivers, Environmental Impacts and Policy Options. OECD Publishing: Paris, 2022. https://doi.org/10.1787/de747aef-en	10.1787/de747aef-en	2022	Within systematic search window by publication year; dataset membership requires original screening registry
3	Sarkar, B.; Dissanayake, P.D.; Bolan, N.S.; Dar, J.Y.; Kumar, M.; Haque, M.N.; Mukhopadhyay, R.; Ramanayaka, S.; Biswas, J.K.; Tsang, D.C.W.; Rinklebe, J.; Ok, Y.S. Challenges and opportunities in sustainable management of microplastics and nanoplastics in the environment. Environ. Res. 2022, 207, 112179. https://doi.org/10.1016/j.envres.2021.112179	10.1016/j.envres.2021.112179	2021	Within systematic search window by publication year; dataset membership requires original screening registry
4	Siddiqui, S.A.; Singh, S.; Bahmid, N.A.; Shyu, D.J.H.; Domínguez, R.; Lorenzo, J.M.; Pereira, J.A.M.;	10.1016/j.scitotenv.2023.164531	2023	Within systematic search window by publication

Manuscript ref.	Bibliographic record	DOI	Year	Traceability status
	Câmara, J.S. Polystyrene microplastic particles in the food chain: Characteristics and toxicity—A review. Sci. Total Environ. 2023, 892, 164531. https://doi.org/10.1016/j.scitotenv.2023.164531			year; dataset membership requires original screening registry
5	Babaremu, K.O.; Okoya, S.A.; Hughes, E.; Tijani, B.; Teidi, D.; Akpan, A.; Igwe, J.; Krarer, S.; Oyinlola, M.; Akinlabi, E.T. Sustainable plastic waste management in a circular economy. Heliyon 2022, 8, e09984. https://doi.org/10.1016/j.heliyon.2022.e09984	10.1016/j.heliyon.2022.e09984	2022	Within systematic search window by publication year; dataset membership requires original screening registry
6	Mushtaq, S.; Jamil, F.; Inayat, A.; Ghenai, C.; Shanableh, A. The green revolution in plastics: Unveiling breakthroughs, applications, and the path forward. Curr. Opin. Green Sustain. Chem. 2024, 49, 100950. https://doi.org/10.1016/j.cogsc.2024.100950	10.1016/j.cogsc.2024.100950	2024	Within systematic search window by publication year; dataset membership requires original screening registry
7	Shamsuyeva, M.; Endres, H.-J. Plastics in the context of the circular economy and sustainable plastics recycling: Comprehensive review on research development, standardization and market. Compos. Part C Open Access 2021, 6, 100168. https://doi.org/10.1016/j.jcomc.2021.100168	10.1016/j.jcomc.2021.100168	2021	Within systematic search window by publication year; dataset membership requires original screening registry
8	Jiao, H.; Ali, S.S.; Alsharbaty, M.H.M.; Elsamahy, T.; Abdelkarim, E.; Schagerl, M.; Al-Tohamy, R.; Sun, J. A critical review on plastic waste life cycle assessment and management: Challenges, research gaps, and future perspectives. Ecotoxicol. Environ. Saf. 2024, 271, 115942. https://doi.org/10.1016/j.ecoenv.2024.115942	10.1016/j.ecoenv.2024.115942	2024	Within systematic search window by publication year; dataset membership requires original screening registry
9	Raj, S.A.; Muthukumar, E.; Jayakrishna, K. A case study of 3D printed PLA and its mechanical properties. Mater. Today Proc. 2018, 5, 11219–11226. https://doi.org/10.1016/j.matpr.2018.01.146	10.1016/j.matpr.2018.01.146	2018	Within systematic search window by publication year; dataset membership requires original screening registry

Manuscript ref.	Bibliographic record	DOI	Year	Traceability status
10	George, A.; Sanjay, M.R.; Srisuk, R.; Parameswaranpillai, J.; Siengchin, S. A comprehensive review on chemical properties and applications of biopolymers and their composites. Int. J. Biol. Macromol. 2020, 154, 329–338. https://doi.org/10.1016/j.ijbiomac.2020.03.120	10.1016/j.ijbiomac.2020.03.120	2020	Within systematic search window by publication year; dataset membership requires original screening registry
11	Kolchakova, G.; Kiryakova, D. A comparative evaluation of the properties of eggshell–polyvinyl chloride films obtained by solution casting and co-precipitation techniques. Appl. Eng. Lett. 2025, 10, 136–145. https://doi.org/10.46793/aeletters.2025.10.3.2	10.46793/aeletters.2025.10.3.2	2025	Post-cutoff contextual update (outside 1998–2024 systematic window)
12	Alami, M.; Mokaddem, A.; Belkheir, M.; Rouissat, M.; Doumi, B.; Ziani, D. Improving the performance of hybrid biocomposite materials using epoxy, vinyl ester and polyimide polymer matrix: Comparative study. Appl. Eng. Lett. 2024, 9, 64–75. https://doi.org/10.46793/aeletters.2024.9.2.1	10.46793/aeletters.2024.9.2.1	2024	Within systematic search window by publication year; dataset membership requires original screening registry
13	Lazăr, S.; Dobrotă, D.; Ciofu, F.C.; Stoica, A. Sheep-wool-fiber-reinforced rigid polyurethane composites: Comparative effects of alkaline, oxidative, and APTES treatments. J. Compos. Biodegrad. Polym. 2026, 14, 127–141. https://doi.org/10.12974/2311-8717.2026.14.11	10.12974/2311-8717.2026.14.11	2026	Post-cutoff contextual update (outside 1998–2024 systematic window)
14	Gajević, S.; Ivanović, L.; Skulić, A.; Stojanović, B. A review on mechanical properties of aluminium-based metal matrix nanocomposites. Tribol. Mater. 2023, 2, 114–127. https://doi.org/10.46793/tribomat.2023.014	10.46793/tribomat.2023.014	2023	Within systematic search window by publication year; dataset membership requires original screening registry
15	Lazăr, S.; Dobrotă, D.; Breaz, R.-E.; Racz, S.-G. Eco-design of polymer matrix composite parts: A review. Polymers 2023, 15, 3634. https://doi.org/10.3390/polym15173634	10.3390/polym15173634	2023	Within systematic search window by publication year; dataset membership requires original

Manuscript ref.	Bibliographic record	DOI	Year	Traceability status
				screening registry
16	Rosenboom, J.-G.; Langer, R.; Traverso, G. Bioplastics for a circular economy. Nat. Rev. Mater. 2022, 7, 117–137. https://doi.org/10.1038/s41578-021-00407-8	10.1038/s41578-021-00407-8	2022	Within systematic search window by publication year; dataset membership requires original screening registry
17	Ali, S.S.; Abdelkarim, E.A.; Elsamahy, T.; Al-Tohamy, R.; Li, F.; Kornaros, M.; Zuorro, A.; Zhu, D.; Sun, J. Bioplastic production in terms of life cycle assessment: A state-of-the-art review. Environ. Sci. Ecotechnol. 2023, 15, 100254. https://doi.org/10.1016/j.ese.2023.100254	10.1016/j.ese.2023.100254	2023	Within systematic search window by publication year; dataset membership requires original screening registry
18	Velasquez, S.T.R.; Hu, Q.; Kramm, J.; Santin, V.C.; Völker, C.; Wurm, F.R. Plastics of the Future? An Interdisciplinary Review on Biobased and Biodegradable Polymers: Progress in Chemistry, Societal Views, and Environmental Implications. Angew. Chem. Int. Ed. 2025, 64, e202423406. https://doi.org/10.1002/anie.202423406	10.1002/anie.202423406	2025	Post-cutoff contextual update (outside 1998–2024 systematic window)
19	Koçak Mutlu, G.; Taskin, E.G.; Karahan, M.; Kara, A.; Üstün, G.E. Production, characterization and life cycle analysis of ZnO doped LDPE films. J. Vinyl Addit. Technol. 2025, 31, 667–681. https://doi.org/10.1002/vnl.22199	10.1002/vnl.22199	2025	Post-cutoff contextual update (outside 1998–2024 systematic window)
20	Karahan, M.; Iftekhar, H. Manufacturing Processes. In Textile Composites and Sustainability: A Circular Approach; Nawab, Y.; Shafqat, A.R.; Hussain, M., Eds.; CRC Press: Boca Raton, FL, USA, 2026; pp. 150–170. https://doi.org/10.1201/9781003604853-7	10.1201/9781003604853-7	2026	Post-cutoff contextual update (outside 1998–2024 systematic window)
21	Şchiopu, E.C.; Lazăr, S.; Dobrotă, D.; Cofaru, N.F.; Drăgan, N.-Ş. Interlocking geometric interfaces for functionally-adapted localized reinforcement in polymer matrix composites: Parametric FEA design	10.1016/j.jmrt.2026.06.084	2026	Post-cutoff contextual update (outside 1998–2024 systematic window)

Manuscript ref.	Bibliographic record	DOI	Year	Traceability status
	rules and experimental validation. J. Mater. Res. Technol. 2026, 43, 760–788. https://doi.org/10.1016/j.jmrt.2026.06.084			
22	Chen, S.; Hu, Y.H. Advancements and future directions in waste plastics recycling: From mechanical methods to innovative chemical processes. Chem. Eng. J. 2024, 493, 152727. https://doi.org/10.1016/j.cej.2024.152727	10.1016/j.cej.2024.152727	2024	Within systematic search window by publication year; dataset membership requires original screening registry
23	Deng, Y.; Zhang, Q.; Qu, D.-H.; Tian, H.; Feringa, B.L. A chemically recyclable crosslinked polymer network enabled by orthogonal dynamic covalent chemistry. Angew. Chem. Int. Ed. 2022, 61, e202209100. https://doi.org/10.1002/anie.202209100	10.1002/anie.202209100	2022	Within systematic search window by publication year; dataset membership requires original screening registry
24	Fagnani, D.E.; Tami, J.L.; Copley, G.; Clemons, M.N.; Getzler, Y.D.Y.L.; McNeil, A.J. 100th Anniversary of Macromolecular Science Viewpoint: Redefining Sustainable Polymers. ACS Macro Lett. 2021, 10, 41–53. https://doi.org/10.1021/acsmacrolett.0c00789	10.1021/acsmacrolett.0c00789	2021	Within systematic search window by publication year; dataset membership requires original screening registry
25	Lazăr, S.; Rotaru, I.M.; Dobrotă, D. Thermal stability and degradation behavior of bio-based and synthetic fiber-reinforced composites: A TGA study. Acta Tech. Napoc. Appl. Math. Mech. Eng. 2025, 68, 2–3S. Available online: https://atna-mam.utcluj.ro/index.php/Acta/article/view/2841		2025	Post-cutoff contextual update (outside 1998–2024 systematic window)
26	He, X.; Shi, X.; Chung, C.; Lei, Z.; Zhang, W.; Yu, K. A sustainable manufacturing method of thermoset composites based on covalent adaptable network polymers. Compos. Part B Eng. 2021, 221, 109004. https://doi.org/10.1016/j.compositesb.2021.109004	10.1016/j.compositesb.2021.109004	2021	Within systematic search window by publication year; dataset membership requires original screening registry
27	Emadian, S.M.; Onay, T.T.; Demirel, B. Biodegradation of bioplastics in natural environments. Waste Manag. 2017, 59, 526–536. https://doi.org/10.1016/j.wasman.2016.	10.1016/j.wasman.2016.10.006	2016	Within systematic search window by publication year; dataset membership

Manuscript ref.	Bibliographic record	DOI	Year	Traceability status
	10.006			requires original screening registry
28	Ali, W.; Ali, H.; Gillani, S.; Zinck, P.; Souissi, S. Polylactic acid synthesis, biodegradability, conversion to microplastics and toxicity: A review. Environ. Chem. Lett. 2023, 21, 1761–1786. https://doi.org/10.1007/s10311-023-01564-8	10.1007/s10311-023-01564-8	2023	Within systematic search window by publication year; dataset membership requires original screening registry
29	McQueen, C.M.A.; Tamburini, D.; Łucejko, J.J.; Braovac, S.; Gambineri, F.; Modugno, F.; Colombini, M.P.; Kutzke, H. New insights into the degradation processes and influence of the conservation treatment in alum-treated wood from the Oseberg collection. Microchem. J. 2017, 132, 119–129. https://doi.org/10.1016/j.microc.2017.01.010	10.1016/j.microc.2017.01.010	2017	Within systematic search window by publication year; dataset membership requires original screening registry
30	Kalia, V.C.; Singh Patel, S.K.; Shanmugam, R.; Lee, J.-K. Polyhydroxyalkanoates: Trends and advances toward biotechnological applications. Bioresour. Technol. 2021, 326, 124737. https://doi.org/10.1016/j.biortech.2021.124737	10.1016/j.biortech.2021.124737	2021	Within systematic search window by publication year; dataset membership requires original screening registry
31	Wu, F.; Misra, M.; Mohanty, A.K. Challenges and new opportunities on barrier performance of biodegradable polymers for sustainable packaging. Prog. Polym. Sci. 2021, 117, 101395. https://doi.org/10.1016/j.progpolymsci.2021.101395	10.1016/j.progpolymsci.2021.101395	2021	Within systematic search window by publication year; dataset membership requires original screening registry
32	Ong, C.; Falca, G.; Huang, T.; Liu, J.; Manchanda, P.; Chisca, S.; Nunes, S.P. Green synthesis of thin-film composite membranes for organic solvent nanofiltration. ACS Sustain. Chem. Eng. 2020, 8, 11541–11548. https://doi.org/10.1021/acssuschemeng.0c02320	10.1021/acssuschemeng.0c02320	2020	Within systematic search window by publication year; dataset membership requires original screening registry
33	Fehér, Z.; Kiss, J.; Kisszékelyi, P.; Molnár, J.; Huszthy, P.; Kárpáti, L.; Kupai, J. Optimisation of PET glycolysis by applying recyclable heterogeneous organocatalysts. Green Chem. 2022, 24, 8447–8459.	10.1039/D2GC02860C	2022	Within systematic search window by publication year; dataset membership requires original

Manuscript ref.	Bibliographic record	DOI	Year	Traceability status
	https://doi.org/10.1039/D2GC02860C			screening registry
34	Aversa, C.; Barletta, M.; Cappiello, G.; Gisario, A. Compatibilization strategies and analysis of morphological features of poly(butylene adipate-co-terephthalate) (PBAT)/poly(lactic acid) PLA blends: A state-of-art review. Eur. Polym. J. 2022, 173, 111304. https://doi.org/10.1016/j.eurpolymj.2022.111304	10.1016/j.eurpolymj.2022.111304	2022	Within systematic search window by publication year; dataset membership requires original screening registry
35	Murariu, M.; Dubois, P. PLA composites: From production to properties. Adv. Drug Deliv. Rev. 2016, 107, 17–46. https://doi.org/10.1016/j.addr.2016.04.003	10.1016/j.addr.2016.04.003	2016	Within systematic search window by publication year; dataset membership requires original screening registry
36	Mohanty, A.K.; Wu, F.; Mincheva, R.; Hakkarainen, M.; Raquez, J.-M.; Mielewski, D.F.; Narayan, R.; Netravali, A.N.; Misra, M. Sustainable polymers. Nat. Rev. Methods Primers 2022, 2, 46. https://doi.org/10.1038/s43586-022-00124-8	10.1038/s43586-022-00124-8	2022	Within systematic search window by publication year; dataset membership requires original screening registry
37	Fredi, G.; Dorigato, A. Recycling of bioplastic waste: A review. Adv. Ind. Eng. Polym. Res. 2021, 4, 159–177. https://doi.org/10.1016/j.aiepr.2021.06.006	10.1016/j.aiepr.2021.06.006	2021	Within systematic search window by publication year; dataset membership requires original screening registry
38	Koller, M.; Mukherjee, A. A new wave of industrialization of PHA biopolyesters. Bioengineering 2022, 9, 74. https://doi.org/10.3390/bioengineering9020074	10.3390/bioengineering9020074	2022	Within systematic search window by publication year; dataset membership requires original screening registry
39	Anjum, A.; Zuber, M.; Zia, K.M.; Noreen, A.; Anjum, M.N.; Tabasum, S. Microbial production of polyhydroxyalkanoates (PHAs) and its copolymers: A review of recent advancements. Int. J. Biol. Macromol. 2016, 89, 161–174. https://doi.org/10.1016/j.ijbiomac.2016.	10.1016/j.ijbiomac.2016.04.069	2016	Within systematic search window by publication year; dataset membership requires original screening registry

Manuscript ref.	Bibliographic record	DOI	Year	Traceability status
	04.069			
40	Cywar, R.M.; Rorrer, N.A.; Hoyt, C.B.; Beckham, G.T.; Chen, E.Y.-X. Bio-based polymers with performance-advantaged properties. Nat. Rev. Mater. 2022, 7, 83–103. https://doi.org/10.1038/s41578-021-00363-3	10.1038/s41578-021-00363-3	2022	Within systematic search window by publication year; dataset membership requires original screening registry
41	Iglesias, J.; Martínez-Salazar, I.; Maireles-Torres, P.; Martin Alonso, D.; Mariscal, R.; López Granados, M. Advances in catalytic routes for the production of carboxylic acids from biomass: A step forward for sustainable polymers. Chem. Soc. Rev. 2020, 49, 5704–5771. https://doi.org/10.1039/d0cs00177e	10.1039/d0cs00177e	2020	Within systematic search window by publication year; dataset membership requires original screening registry
42	Ahmad, M.; Manzoor, K.; Ikram, S. Versatile nature of hetero-chitosan based derivatives as biodegradable adsorbent for heavy metal ions: A review. Int. J. Biol. Macromol. 2017, 105, 190–203. https://doi.org/10.1016/j.ijbiomac.2017.07.008	10.1016/j.ijbiomac.2017.07.008	2017	Within systematic search window by publication year; dataset membership requires original screening registry
43	Pires, P.C.; Mascarenhas-Melo, F.; Pedrosa, K.; Lopes, D.; Lopes, J.; Macário-Soares, A.; Peixoto, D.; Giram, P.S.; Veiga, F.; Paiva-Santos, A.C. Polymer-based biomaterials for pharmaceutical and biomedical applications: A focus on topical drug administration. Eur. Polym. J. 2023, 187, 111868. https://doi.org/10.1016/j.eurpolymj.2023.111868	10.1016/j.eurpolymj.2023.111868	2023	Within systematic search window by publication year; dataset membership requires original screening registry
44	Das, A.; Ringu, T.; Ghosh, S.; Pramanik, N. A comprehensive review on recent advances in preparation, physicochemical characterization, and bioengineering applications of biopolymers. Polym. Bull. 2023, 80, 7247–7312. https://doi.org/10.1007/s00289-022-04443-4	10.1007/s00289-022-04443-4	2023	Within systematic search window by publication year; dataset membership requires original screening registry
45	Udayakumar, G.P.; Muthusamy, S.; Selvaganesh, B.; Sivarajasekar, N.; Rambabu, K.; Sivamani, S.; Sivakumar, N.; Maran, J.P.; Hosseini-	10.1016/j.biotechadv.2021.107815	2021	Within systematic search window by publication year; dataset

Manuscript ref.	Bibliographic record	DOI	Year	Traceability status
	Bandegharaei, A. Ecofriendly biopolymers and composites: Preparation and their applications in water treatment. Biotechnol. Adv. 2021, 52, 107815. https://doi.org/10.1016/j.biotechadv.2021.107815			membership requires original screening registry
46	Moshood, T.D.; Nawanir, G.; Mahmud, F.; Mohamad, F.; Ahmad, M.H.; AbdulGhani, A. Biodegradable plastic applications towards sustainability: A recent innovations in the green product. Clean. Eng. Technol. 2022, 6, 100404. https://doi.org/10.1016/j.clet.2022.100404	10.1016/j.clet.2022.100404	2022	Within systematic search window by publication year; dataset membership requires original screening registry
47	Zhu, Y.; Romain, C.; Williams, C.K. Sustainable polymers from renewable resources. Nature 2016, 540, 354–362. https://doi.org/10.1038/nature21001	10.1038/nature21001	2016	Within systematic search window by publication year; dataset membership requires original screening registry
48	Manker, L.P.; Dick, G.R.; Demongeot, A.; Hedou, M.A.; Rayroud, C.; Rambert, T.; Jones, M.J.; Sulaeva, I.; Vieli, M.; Leterrier, Y.; Potthast, A.; Maréchal, F.; Michaud, V.; Klok, H.-A.; Luterbacher, J.S. Sustainable polyesters via direct functionalization of lignocellulosic sugars. Nat. Chem. 2022, 14, 976–984. https://doi.org/10.1038/s41557-022-00974-5	10.1038/s41557-022-00974-5	2022	Within systematic search window by publication year; dataset membership requires original screening registry
49	Cywar, R.M.; Beckham, G.T. Producing performance-advantaged bioplastics. Nat. Chem. 2022, 14, 967–969. https://doi.org/10.1038/s41557-022-01030-y	10.1038/s41557-022-01030-y	2022	Within systematic search window by publication year; dataset membership requires original screening registry
50	Cao, D. Mechanical enhancement of natural-fiber-reinforced composites modified with recycled thermoset composite fillers. J. Reinf. Plast. Compos. 2025, 44, 1776–1788. https://doi.org/10.1177/07316844241247896	10.1177/07316844241247896	2025	Post-cutoff contextual update (outside 1998–2024 systematic window)
51	Saini, P.; Arora, M.; Kumar, M.N.V.R.	10.1016/j.addr.2016.06.0	2016	Within systematic

Manuscript ref.	Bibliographic record	DOI	Year	Traceability status
	Poly(lactic acid) blends in biomedical applications. Adv. Drug Deliv. Rev. 2016, 107, 47–59. https://doi.org/10.1016/j.addr.2016.06.014	14		search window by publication year; dataset membership requires original screening registry
52	Wang, L.; Li, G.; Deng, Y. Diamine biosynthesis: Research progress and application prospects. Appl. Environ. Microbiol. 2020, 86, e01972-20. https://doi.org/10.1128/aem.01972-20	10.1128/aem.01972-20	2020	Within systematic search window by publication year; dataset membership requires original screening registry
53	Banoriya, D.; Purohit, R.; Dwivedi, R.K. Advanced Application of Polymer Based Biomaterials. Mater. Today Proc. 2017, 4, 3534–3541. https://doi.org/10.1016/j.matpr.2017.02.244	10.1016/j.matpr.2017.02.244	2017	Within systematic search window by publication year; dataset membership requires original screening registry
54	Moholkar, D.N.; Sadalage, P.S.; Peixoto, D.; Paiva-Santos, A.C.; Pawar, K.D. Recent advances in biopolymer-based formulations for wound healing applications. Eur. Polym. J. 2021, 160, 110784. https://doi.org/10.1016/j.eurpolymj.2021.110784	10.1016/j.eurpolymj.2021.110784	2021	Within systematic search window by publication year; dataset membership requires original screening registry
55	Nasrollahzadeh, M.; Sajjadi, M.; Iravani, S.; Varma, R.S. Starch, cellulose, pectin, gum, alginate, chitin and chitosan derived (nano)materials for sustainable water treatment: A review. Carbohydr. Polym. 2021, 251, 116986. https://doi.org/10.1016/j.carbpol.2020.116986	10.1016/j.carbpol.2020.116986	2020	Within systematic search window by publication year; dataset membership requires original screening registry
56	Keshvardoostchokami, M.; Majidi, M.; Zamani, A.; Liu, B. A review on the use of chitosan and chitosan derivatives as the bio-adsorbents for the water treatment: Removal of nitrogen-containing pollutants. Carbohydr. Polym. 2021, 273, 118625. https://doi.org/10.1016/j.carbpol.2021.118625	10.1016/j.carbpol.2021.118625	2021	Within systematic search window by publication year; dataset membership requires original screening registry
57	Kazmierczak-Razna, J.; Nowicki, P.; Pietrzak, R. Characterization and application of bio-activated carbons	10.1016/j.powtec.2017.06.062	2017	Within systematic search window by publication

Manuscript ref.	Bibliographic record	DOI	Year	Traceability status
	prepared by direct activation of hay with the use of microwave radiation. Powder Technol. 2017, 319, 302–312. https://doi.org/10.1016/j.powtec.2017.06.062			year; dataset membership requires original screening registry
58	Li, M.; Nguyen, L.; Subramaniyan, B.; Bio, M.; Peer, C.J.; Kindrick, J.; Figg, W.D.; Woo, S.; You, Y. PBPK modeling-based optimization of site-specific chemo-photodynamic therapy with far-red light-activatable paclitaxel prodrug. J. Control. Release 2019, 308, 86–97. https://doi.org/10.1016/j.jconrel.2019.07.010	10.1016/j.jconrel.2019.07.010	2019	Within systematic search window by publication year; dataset membership requires original screening registry
59	Unni, A.B.; Joseph, T.M. Enhancing polymer sustainability: Eco-conscious strategies. Polymers 2024, 16, 1769. https://doi.org/10.3390/polym16131769	10.3390/polym16131769	2024	Within systematic search window by publication year; dataset membership requires original screening registry
60	Yang, H.-S.; Cho, S.; Lee, M.; Eom, Y.; Chae, H.G.; Park, S.-A.; Jang, M.; Oh, D.X.; Hwang, S.Y.; Park, J. Preparation of sustainable fibers from isosorbide: Merits over bisphenol-A based polysulfone. Mater. Des. 2021, 198, 109284. https://doi.org/10.1016/j.matdes.2020.109284	10.1016/j.matdes.2020.109284	2020	Within systematic search window by publication year; dataset membership requires original screening registry
61	Hayes, G.; Laurel, M.; MacKinnon, D.; Zhao, T.; Houck, H.A.; Becer, C.R. Polymers without petrochemicals: Sustainable routes to conventional monomers. Chem. Rev. 2023, 123, 2609–2734. https://doi.org/10.1021/acs.chemrev.2c00354	10.1021/acs.chemrev.2c00354	2023	Within systematic search window by publication year; dataset membership requires original screening registry
62	Li, C.; Zhang, Z.; Yang, Z.; Fang, W.; An, H.; Li, T; Xu, F. Synthesis of bio-based poly(oligoethylene glycols-co-isosorbide carbonate)s with high molecular weight and enhanced mechanical properties via ionic liquid catalyst. React. Funct. Polym. 2020, 155, 104689. https://doi.org/10.1016/j.reactfunctpolym.2020.104689	10.1016/j.reactfunctpolym.2020.104689	2020	Within systematic search window by publication year; dataset membership requires original screening registry

Manuscript ref.	Bibliographic record	DOI	Year	Traceability status
63	Wei, G.; Xu, H.; Chen, L.; Li, Z.; Liu, R. Isosorbide-based high performance UV-curable reactive diluents. Prog. Org. Coat. 2019, 126, 162–167. https://doi.org/10.1016/j.porgcoat.2018.10.028	10.1016/j.porgcoat.2018.10.028	2018	Within systematic search window by publication year; dataset membership requires original screening registry
64	Wang, Z.; Ganewatta, M.S.; Tang, C. Sustainable polymers from biomass: Bridging chemistry with materials and processing. Prog. Polym. Sci. 2020, 101, 101197. https://doi.org/10.1016/j.progpolymsci.2019.101197	10.1016/j.progpolymsci.2019.101197	2019	Within systematic search window by publication year; dataset membership requires original screening registry
65	Shojaei, B.; Najafi, M.; Yazdanbakhsh, A.; Abtahi, M.; Zhang, C. A review on the applications of polyurea in the construction industry. Polym. Adv. Technol. 2021, 32, 2797–2812. https://doi.org/10.1002/pat.5277	10.1002/pat.5277	2021	Within systematic search window by publication year; dataset membership requires original screening registry
66	Chen, K.; Wu, D.; Xia, L.; Cai, Q.; Zhang, Z. Geopolymer concrete durability subjected to aggressive environments – A review of influence factors and comparison with ordinary Portland cement. Constr. Build. Mater. 2021, 279, 122496. https://doi.org/10.1016/j.conbuildmat.2021.122496	10.1016/j.conbuildmat.2021.122496	2021	Within systematic search window by publication year; dataset membership requires original screening registry
67	De Hoe, G.X.; Şucu, T.; Shaver, M.P. Sustainability and polyesters: Beyond metals and monomers to function and fate. Acc. Chem. Res. 2022, 55, 1514–1523. https://doi.org/10.1021/acs.accounts.2c00134	10.1021/acs.accounts.2c00134	2022	Within systematic search window by publication year; dataset membership requires original screening registry
68	Lyu, B.; Wang, Y.; Gao, D.; Guo, S.; Zhou, Y.; Ma, J. Ultra-low reflection electromagnetic interference shielding nanofiber film with effective solar harvesting and self-cleaning. Collagen Leather 2024, 6, 2. https://doi.org/10.1186/s42825-023-00143-5	10.1186/s42825-023-00143-5	2024	Within systematic search window by publication year; dataset membership requires original screening registry
69	Lyu, B.; Guo, Z.; Gao, D.; Zhou, Y.; Guo, S.; Zhu, J.; Ma, J. Ultralight flexible collagen fiber based aerogels derived from leather solid waste for	10.1021/acs.langmuir.4c00611	2024	Within systematic search window by publication year; dataset

Manuscript ref.	Bibliographic record	DOI	Year	Traceability status
	high electromagnetic interference shielding. Langmuir 2024, 40, 9215–9223. https://doi.org/10.1021/acs.langmuir.4c00611			membership requires original screening registry
70	Lazăr, S.; Rotaru, I.M.; Dobrotă, D. Advancing green composites: A comparative analysis of sisal, coir, and glass fiber-reinforced polymers. Acta Tech. Napoc. Appl. Math. Mech. Eng. 2025, 68, 2–3S. Available online: https://atna-mam.utcluj.ro/index.php/Acta/article/view/2840		2025	Post-cutoff contextual update (outside 1998–2024 systematic window)
71	Getme, A.S.; Patel, B. A review: Bio-fiber's as reinforcement in composites of polylactic acid (PLA). Mater. Today Proc. 2020, 26, 2116–2122. https://doi.org/10.1016/j.matpr.2020.02.457	10.1016/j.matpr.2020.02.457	2020	Within systematic search window by publication year; dataset membership requires original screening registry
72	Dobrotă, D.; Lazăr, S.V. Redesign of the geometry of parts produced from PBT composite to improve their operational behavior. Polymers 2021, 13, 2536. https://doi.org/10.3390/polym13152536	10.3390/polym13152536	2021	Within systematic search window by publication year; dataset membership requires original screening registry
73	Khodaei, D.; Álvarez, C.; Mullen, A.M. Biodegradable packaging materials from animal processing co-products and wastes: An overview. Polymers 2021, 13, 2561. https://doi.org/10.3390/polym13152561	10.3390/polym13152561	2021	Within systematic search window by publication year; dataset membership requires original screening registry
74	Chan, W.Y. Proteins in the design of sustainable plastics alternatives. MRS Commun. 2023, 13, 1009–1024. https://doi.org/10.1557/s43579-023-00481-9	10.1557/s43579-023-00481-9	2023	Within systematic search window by publication year; dataset membership requires original screening registry
75	Maraveas, C.; Bayer, I.S.; Bartzanas, T. 4D printing: Perspectives for the production of sustainable plastics for agriculture. Biotechnol. Adv. 2022, 54, 107785. https://doi.org/10.1016/j.biotechadv.2021.107785	10.1016/j.biotechadv.2021.107785	2021	Within systematic search window by publication year; dataset membership requires original screening registry

Manuscript ref.	Bibliographic record	DOI	Year	Traceability status
76	Heinrich, L.A. Future opportunities for bio-based adhesives – advantages beyond renewability. Green Chem. 2019, 21, 1866–1888. https://doi.org/10.1039/c8gc03746a	10.1039/c8gc03746a	2019	Within systematic search window by publication year; dataset membership requires original screening registry
77	Ahmed Ali, B.A.; Sapuan, S.M.; Zainudin, E.S.; Othman, M. Implementation of the expert decision system for environmental assessment in composite materials selection for automotive components. J. Clean. Prod. 2015, 107, 557–567. https://doi.org/10.1016/j.jclepro.2015.05.084	10.1016/j.jclepro.2015.05.084	2015	Within systematic search window by publication year; dataset membership requires original screening registry
78	Ahmed, W.; Alnajjar, F.; Zaneldin, E.; Al-Marzouqi, A.H.; Gochoo, M.; Khalid, S. Implementing FDM 3D printing strategies using natural fibers to produce biomass composite. Materials 2020, 13, 4065. https://doi.org/10.3390/ma13184065	10.3390/ma13184065	2020	Within systematic search window by publication year; dataset membership requires original screening registry
79	Cao, D. Strengthening the interphase of thermoplastic sandwich composites by interleaving carbon nanotube yarns. Mater. Today Commun. 2023, 36, 106655. https://doi.org/10.1016/j.mtcomm.2023.106655	10.1016/j.mtcomm.2023.106655	2023	Within systematic search window by publication year; dataset membership requires original screening registry
80	Ge, J.; Catalanotti, G.; Falzon, B.G.; Higgins, C.; McClory, C.; Thiebot, J.-A.; Zhang, L.; He, M.; Jin, Y.; Sun, D. Process characteristics, damage mechanisms and challenges in machining of fibre reinforced thermoplastic polymer (FRTP) composites: A review. Compos. Part B Eng. 2024, 273, 111247. https://doi.org/10.1016/j.compositesb.2024.111247	10.1016/j.compositesb.2024.111247	2024	Within systematic search window by publication year; dataset membership requires original screening registry
81	Azaza, Y.B.; Van Der Lee, A.; Li, S.; Nasri, M.; Nasri, R. Chitosan/collagen-based hydrogels for sustainable development: Phycocyanin controlled release. Sustain. Chem. Pharm. 2023, 31, 100905. https://doi.org/10.1016/j.scp.2022.1009	10.1016/j.scp.2022.100905	2022	Within systematic search window by publication year; dataset membership requires original screening registry

Manuscript ref.	Bibliographic record	DOI	Year	Traceability status
	05			
82	Dobrotă, R.D.; Ferechide, D.; Pogărășteanu, M.; Moga, M.; Paraschiv, R. Comparative Biomechanical Evaluation of Hook-Plate Versus Bicortical Screw Fixation for Fifth Metatarsal Avulsion Fractures. J. Clin. Med. 2025, 14, 8300. https://doi.org/10.3390/jcm14238300	10.3390/jcm14238300	2025	Post-cutoff contextual update (outside 1998–2024 systematic window)
83	Dobrotă, R.D.; Pogărășteanu, M.; Ferechide, D.; Lebada, I.-C.; Moga, M. Comparative Biomechanical Evaluation of Bicortical Screw Versus Plate Fixation in Jones Fractures of the Fifth Metatarsal Using 3D-Printed Models. J. Clin. Med. 2025, 14, 7449. https://doi.org/10.3390/jcm14207449	10.3390/jcm14207449	2025	Post-cutoff contextual update (outside 1998–2024 systematic window)
84	Dong, X.; Lu, D.; Harris, T.A.L.; Escobar, I.C. Polymers and solvents used in membrane fabrication: A review focusing on sustainable membrane development. Membranes 2021, 11, 309. https://doi.org/10.3390/membranes11050309	10.3390/membranes11050309	2021	Within systematic search window by publication year; dataset membership requires original screening registry
85	Yadav, P.; Ismail, N.; Essalhi, M.; Tysklind, M.; Athanassiadis, D.; Tavajohi, N. Assessment of the environmental impact of polymeric membrane production. J. Membr. Sci. 2021, 622, 118987. https://doi.org/10.1016/j.memsci.2020.118987	10.1016/j.memsci.2020.118987	2020	Within systematic search window by publication year; dataset membership requires original screening registry
86	Keirouz, A.; Wang, Z.; Reddy, V.S.; Nagy, Z.K.; Vass, P.; Buzgo, M.; Ramakrishna, S.; Radacsi, N. The history of electrospinning: Past, present, and future developments. Adv. Mater. Technol. 2023, 8, 2201723. https://doi.org/10.1002/admt.202201723	10.1002/admt.202201723	2023	Within systematic search window by publication year; dataset membership requires original screening registry
87	Atmakuri, A.; Palevicius, A.; Vilkauskas, A.; Janusas, G. Review of hybrid fiber based composites with nano particles—Material properties and applications. Polymers 2020, 12, 2088. https://doi.org/10.3390/polym12092088	10.3390/polym12092088	2088	Post-cutoff contextual update (outside 1998–2024 systematic window)

Manuscript ref.	Bibliographic record	DOI	Year	Traceability status
88	Manivannan, R.K.; Sharma, N.; Kumar, V.; Jayaraj, I.; Vimal, S.; Umesh, M. A comprehensive review on natural macromolecular biopolymers for biomedical applications: Recent advancements, current challenges, and future outlooks. Carbohydr. Polym. Technol. Appl. 2024, 8, 100536. https://doi.org/10.1016/j.carpta.2024.100536	10.1016/j.carpta.2024.100536	2024	Within systematic search window by publication year; dataset membership requires original screening registry
89	Law, K.L.; Sobkowicz, M.J.; Shaver, M.P.; Hahn, M.E. Untangling the chemical complexity of plastics to improve life cycle outcomes. Nat. Rev. Mater. 2024, 9, 657–667. https://doi.org/10.1038/s41578-024-00705-x	10.1038/s41578-024-00705-x	2024	Within systematic search window by publication year; dataset membership requires original screening registry
90	Putra, P.H.M.; Rozali, S.; Abdul Patah, M.F.; Idris, A. A review of microwave pyrolysis as a sustainable plastic waste management technique. J. Environ. Manag. 2022, 303, 114240. https://doi.org/10.1016/j.jenvman.2021.114240	10.1016/j.jenvman.2021.114240	2021	Within systematic search window by publication year; dataset membership requires original screening registry
91	Kulkarni, P.; Ravekar, V.; Rama Rao, P.; Waigokar, S.; Hingankar, S. Recycling of waste HDPE and PP plastic in preparation of plastic brick and its mechanical properties. Clean. Mater. 2022, 5, 100113. https://doi.org/10.1016/j.clema.2022.100113	10.1016/j.clema.2022.100113	2022	Within systematic search window by publication year; dataset membership requires original screening registry
92	Jambeck, J.R.; Geyer, R.; Wilcox, C.; Siegler, T.R.; Perryman, M.; Andrady, A.; Narayan, R.; Law, K.L. Plastic waste inputs from land into the ocean. Science 2015, 347, 768–771. https://doi.org/10.1126/science.1260352	10.1126/science.1260352	2015	Within systematic search window by publication year; dataset membership requires original screening registry
93	Saxon, D.J.; Luke, A.M.; Sajjad, H.; Tolman, W.B.; Reineke, T.M. Next-generation polymers: Isosorbide as a renewable alternative. Prog. Polym. Sci. 2020, 101, 101196. https://doi.org/10.1016/j.progpolymsci.2019.101196	10.1016/j.progpolymsci.2019.101196	2019	Within systematic search window by publication year; dataset membership requires original screening registry
94	Dobrotă, D.; Lazăr, S.V. Ecodesign enhancement of polymeric resins:	10.3390/polym16243458	2024	Within systematic search window

Manuscript ref.	Bibliographic record	DOI	Year	Traceability status
	Reinforcing with synthetic and natural fibers using theory of inventive problem solving–algorithm of inventive problem solving for sustainable composite design. Polymers 2024, 16, 3458. https://doi.org/10.3390/polym16243458			by publication year; dataset membership requires original screening registry
95	Guarda, C.; Caseiro, J.; Pires, A. Machine learning to enhance sustainable plastics: A review. J. Clean. Prod. 2024, 474, 143602. https://doi.org/10.1016/j.jclepro.2024.143602	10.1016/j.jclepro.2024.143602	2024	Within systematic search window by publication year; dataset membership requires original screening registry
96	Dobrotă, D.; Lazăr, S.V. Ultrasonic welding of PBT-GF30 (70% polybutylene terephthalate + 30% fiber glass) and expanded polytetrafluoroethylene (e-PTFE). Polymers 2021, 13, 298. https://doi.org/10.3390/polym13020298	10.3390/polym13020298	2021	Within systematic search window by publication year; dataset membership requires original screening registry
97	Voss, G.T.; Gularte, M.S.; De Oliveira, R.L.; Luchese, C.; Fajardo, A.R.; Wilhelm, E.A. Biopolymeric films as delivery vehicles for controlled release of hydrocortisone: Promising devices to treat chronic skin diseases. Mater. Sci. Eng. C 2020, 114, 111074. https://doi.org/10.1016/j.msec.2020.111074	10.1016/j.msec.2020.111074	2020	Within systematic search window by publication year; dataset membership requires original screening registry
98	Verschoor, J.-A.; Kusumawardhani, H.; Ram, A.F.J.; De Winde, J.H. Toward microbial recycling and upcycling of plastics: Prospects and challenges. Front. Microbiol. 2022, 13, 821629. https://doi.org/10.3389/fmicb.2022.821629	10.3389/fmicb.2022.821629	2022	Within systematic search window by publication year; dataset membership requires original screening registry
99	Yang, Y.; Xiong, Z.; Zhang, L.; Tang, Z.; Zhang, R.; Zhu, J. Isosorbide dioctoate as a "green" plasticizer for poly(lactic acid). Mater. Des. 2016, 91, 262–268. https://doi.org/10.1016/j.matdes.2015.11.065	10.1016/j.matdes.2015.11.065	2015	Within systematic search window by publication year; dataset membership requires original screening registry
100	Baley, C.; Davies, P.; Troalen, W.; Chamley, A.; Dinham-Price, I.;	10.1016/j.pmatsci.2024.101307	2024	Within systematic search window

Manuscript ref.	Bibliographic record	DOI	Year	Traceability status
	Marchandise, A.; Keryvin, V. Sustainable polymer composite marine structures: Developments and challenges. Prog. Mater. Sci. 2024, 145, 101307. https://doi.org/10.1016/j.pmatsci.2024.101307			by publication year; dataset membership requires original screening registry
101	Goanță, V.; Hadăr, A.; Leițoiu, B. Experimental procedure designed to determine the elastic characteristics of fiber-reinforced polymeric composite materials. Mater. Plast. 2010, 47, 450–456.		2010	Within systematic search window by publication year; dataset membership requires original screening registry
102	Jehanno, C.; Alty, J.W.; Roosen, M.; De Meester, S.; Dove, A.P.; Chen, E.Y.-X.; Leibfarth, F.A.; Sardon, H. Critical advances and future opportunities in upcycling commodity polymers. Nature 2022, 603, 803–814. https://doi.org/10.1038/s41586-021-04350-0	10.1038/s41586-021-04350-0	2022	Within systematic search window by publication year; dataset membership requires original screening registry
103	Yeom, S.-J.; Le, T.-K.; Yun, C.-H. P450-driven plastic-degrading synthetic bacteria. Trends Biotechnol. 2022, 40, 166–179. https://doi.org/10.1016/j.tibtech.2021.06.003	10.1016/j.tibtech.2021.06.003	2021	Within systematic search window by publication year; dataset membership requires original screening registry

Table S2. Source-linked LCA evidence extraction matrix supporting Tables 4 and 7

This table follows the revised manuscript's evidence-handling rule: quantitative values are retained only when a comparable production-stage basis is available; heterogeneous studies are not forced into a universal polymer-level value. The entries below therefore reproduce the manuscript-level evidence statements and identify the source groups used for each polymer.

Polymer	Revised climate-evidence statement	Supporting refs.	Harmonization basis	Key sensitivity variables	Use in synthesis
PLA	Production-stage GHG can be lower under favorable energy/feedstock assumptions, but no universal	[16,17,36]	1 kg polymer resin at factory gate when defensibly harmonizable	Electricity mix; agricultural inputs; allocation; biogenic carbon; plant assumptions	Quantitative only for comparable production-stage studies; otherwise scenario

Polymer	Revised climate-evidence statement	Supporting refs.	Harmonization basis	Key sensitivity variables	Use in synthesis
	value is imposed.				interpretation
PHA	Not pooled into a universal value; strongly route-dependent.	[17,38,39]	Production-stage basis only when substrate/fermentation/recovery boundaries are comparable	Substrate; fermentation productivity; aeration; downstream recovery; co-product treatment	Primarily route-specific; non-comparable studies retained qualitatively
PBS	Not pooled; depends on precursor route, modeled scale, allocation and energy system.	[17,31,36]	Production-stage basis where bio-/fossil-precursor boundary is explicit	Precursor route; process energy; allocation; scale	No single resin-level value
PEF	Potential GHG reductions relative to PET under favorable bio-based assumptions; magnitude is route- and scale-dependent.	[16,36,40]	Matched production-stage comparison where FDCA route and energy basis are explicit	FDCA route; process scale; allocation; electricity/heat	Comparative interpretation; no universal constant
PET	Conventional comparator; only matched-boundary LCA/LCI comparisons are used.	[2,7,8]	Matched resin-level production boundary	Geography; recycled content; process dataset; system boundary	Comparator; no global constant
PP	Conventional comparator; interpretation restricted to matched boundaries and regional data.	[2,7,8]	Matched resin-level production boundary	Feedstock; geography; energy supply; recycling assumptions	Comparator; no global constant
HDPE	Conventional comparator; interpretation restricted to matched boundaries and regional data.	[2,7,8]	Matched resin-level production boundary	Process dataset; energy system; recycled content; geography	Comparator; no global constant

Table S3. Evidence-handling and harmonization rules corresponding to Figure 11 and Appendix A

The former 0–100 normalized scoring table is intentionally removed because the revised manuscript no longer uses cross-criterion normalization. Table S5 documents the decision rules that determine whether a study contributes to quantitative production-stage synthesis or remains in categorical/scenario interpretation.

Source-study situation	Decision rule	Destination in synthesis
Cradle-to-gate; 1 kg resin/polymer at factory gate	Include directly if allocation and biogenic-carbon treatment are reported or interpretable	Quantitative production-stage synthesis
Cradle-to-grave with separately reported production stage	Use only the separately reported production/resin contribution	Quantitative production-stage synthesis; EoL/use kept separate
Cradle-to-grave total without separable stages	Do not back-calculate by subtracting assumed EoL/use burdens	Scenario/application interpretation only
Product-based FU with explicit polymer mass and equivalent function	Convert only when the source provides sufficient mass/function information without new assumptions	Quantitative stratum if defensible; otherwise application-specific
Incompatible allocation, unreported biogenic-carbon treatment, or non-separable boundary	Do not pool quantitatively	Qualitative/scenario interpretation
Energy indicator	Retain MJ/kg only if primary/fossil/cumulative-energy definition is explicit	Separate energy synthesis; no merging of unlike energy metrics
Categorical recyclability/biodegradability/resource-efficiency evidence	Do not transform into synthetic 0–100 scores	Report criterion-level evidence with source and conditions
Outlier or extreme value	Retain only when methodological transparency supports it; otherwise exclude from aggregation	Document inclusion/exclusion reason in extraction sheet

Minimum extraction fields for auditability: bibliographic source; material/grade; original functional unit; system boundary; geography/electricity mix; allocation/biogenic-carbon convention; GHG result in original units; harmonized value only when eligible; energy result with metric definition; TEA result with currency-year/plant scale; use in synthesis and reason for exclusion from pooling where applicable.

Table S4. Techno-economic evidence matrix supporting Tables 8 and 10

The revised manuscript does not construct a de novo cost model and does not retain universal polymer-to-polymer cost multipliers. The table below therefore records only the source-linked economic constraints and semi-quantitative maturity descriptors used in the manuscript.

System	Source-linked economic evidence	Supporting refs.	Class	CapEx	Learning	Maturity	Pooling rule
PET/PP/HDPE	Commodity reference systems; prices vary by grade, region, feedstock and year; mature production/recover	[2,7]	Commodity	Low	Low	Commercial / mature	No universal resin price or multiplier

System	Source-linked economic evidence	Supporting refs.	Class	CapEx	Learning	Maturity	Pooling rule
	y infrastructure provides baseline.						
PLA	Commercial cost depends on feedstock, fermentation efficiency, purification, energy price and plant scale.	[36]	Transition	Medium	Med–High	Commercial	No universal multiplier retained
PHA	Commercialization constrained by fermentation productivity, feedstock, downstream recovery, product consistency and scale.	[36,38,39]	Niche	High	High	Commercial niche; route-specific	No universal multiplier retained
PBS	Economics depend on precursor route, process integration, purification, energy demand and scale.	[36]	Specific	Med–High	Medium	Commercial / limited market	No universal multiplier retained
PEF	Evidence shaped by early-scale production, FDCA supply, capital intensity and market development.	[36,40]	Pre-commercial / early scale	High	High	Demonstration / early commercial; uncertain	No universal multiplier retained; higher uncertainty
Natural polymers	Highly application-, feedstock- and processing-dependent; cost evidence is not harmonized.	[44,73,74]	Variable	Variable	Medium	Lab–commercial; application-specific	No pooled TEA estimate

Table S5. Evidence-to-claim and confidence matrix for the principal conclusions

Confidence labels mirror the revised manuscript and are conditional on the stated analytical boundary. They are not numerical quality scores.

Principal conclusion	Evidence basis	Supporting refs.	Confidence	Interpretive boundary
PLA can reduce GHG emissions relative to PET under low-carbon	Source-linked production-stage LCA synthesis	[16,17,36]	Moderate	Electricity mix, land-use change, allocation, biogenic carbon and FU can change the

Principal conclusion	Evidence basis	Supporting refs.	Confidence	Interpretive boundary
production assumptions.				comparison.
PHA offers strong biodegradation potential in selected environments.	Biodegradation and LCA literature with heterogeneous conditions	[17,28,30,38,39]	Conditional-to-moderate	Polymer composition, geometry, temperature, microbial activity and managed vs natural conditions must be specified.
Recyclable polymers often perform favorably in mature recovery systems.	Recycling/circularity evidence and EoL scenario matrix	[7,8,22]	Moderate	Requires effective collection, sorting, contamination control and displacement of virgin resin.
Compostable polymers provide intended EoL benefits only where compatible collection and industrial composting exist.	Bioplastics/biodegradation evidence and EoL scenarios	[16,28,31]	Conditional	Facility acceptance, certification, correct sorting and controlled composting conditions are required.
PEF recycling integration is potential rather than universally established.	PEF material/function and system-compatibility evidence	[16,36,40]	Conditional	Requires sorting, contamination control and industrial-scale validation.
Economic scalability remains a limiting factor for PHA, PBS and early-scale PEF.	Source-linked TEA constraints and maturity clustering	[36,38,39,40]	Weak-to-moderate	Feedstock, scale, energy price, utilization, capital intensity and policy assumptions vary.
A universal polymer ranking is not supported by the heterogeneous evidence base.	LCA boundary analysis, EoL scenarios, TEA and infrastructure synthesis	[7,8,16,17,36]	Moderate	Relative performance changes with region, energy system, recovery infrastructure, function and maturity.
Carbon footprint alone is insufficient for sustainability assessment.	Multi-indicator LCA methodological discussion	[16,17]	Moderate	Land, water, eutrophication, acidification and functional performance may alter conclusions.
